# Multisite assessment of reproducibility in high‐content cell migration imaging data

**DOI:** 10.15252/msb.202211490

**Published:** 2023-04-17

**Authors:** Jianjiang Hu, Xavier Serra‐Picamal, Gert‐Jan Bakker, Marleen Van Troys, Sabina Winograd‐Katz, Nil Ege, Xiaowei Gong, Yuliia Didan, Inna Grosheva, Omer Polansky, Karima Bakkali, Evelien Van Hamme, Merijn van Erp, Manon Vullings, Felix Weiss, Jarama Clucas, Anna M Dowbaj, Erik Sahai, Christophe Ampe, Benjamin Geiger, Peter Friedl, Matteo Bottai, Staffan Strömblad

**Affiliations:** ^1^ Department of Biosciences and Nutrition Karolinska Institutet Stockholm Sweden; ^2^ Department of Medical BioSciences Radboud University Medical Center Nijmegen The Netherlands; ^3^ Department of Biomolecular Medicine Ghent University Ghent Belgium; ^4^ Department of Immunology and Regenerative Biology Weizmann Institute of Science Rehovot Israel; ^5^ The Francis Crick Institute London UK; ^6^ Bio Imaging Core, VIB Center for Inflammation Research Ghent Belgium; ^7^ Division of Biostatistics, Institute of Environmental Medicine Karolinska Institutet Stockholm Sweden

**Keywords:** batch effect removal, cell migration, high‐content imaging, reproducibility, variability, Methods & Resources

## Abstract

High‐content image‐based cell phenotyping provides fundamental insights into a broad variety of life science disciplines. Striving for accurate conclusions and meaningful impact demands high reproducibility standards, with particular relevance for high‐quality open‐access data sharing and meta‐analysis. However, the sources and degree of biological and technical variability, and thus the reproducibility and usefulness of meta‐analysis of results from live‐cell microscopy, have not been systematically investigated. Here, using high‐content data describing features of cell migration and morphology, we determine the sources of variability across different scales, including between laboratories, persons, experiments, technical repeats, cells, and time points. Significant technical variability occurred between laboratories and, to lesser extent, between persons, providing low value to direct meta‐analysis on the data from different laboratories. However, batch effect removal markedly improved the possibility to combine image‐based datasets of perturbation experiments. Thus, reproducible quantitative high‐content cell image analysis of perturbation effects and meta‐analysis depend on standardized procedures combined with batch correction.

## Graphical abstract

Analyses of the sources of variability of cell migration data obtained by live cell imaging produced by independent labs with identical protocol and key reagents show that the highest variability occurs between labs and the variance can be substantially reduced by batch effect removal.

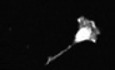

## Introduction

High‐content cell imaging enables great advances in many life sciences fields, such as cell biology, biomedicine, and drug development. Modern microscope setups can generate vast amounts of high‐resolution data, rich across multiple dimensions, including high spatial and temporal resolution, to differentiate cell structures in a multiplex manner and to spatially resolve and quantify gene or protein expression, as well as the effects of drug perturbation (Boutros *et al*, 2015; Bray *et al*, 2016). Accompanying these technological advances, initiatives have emerged to host and make image‐based datasets publicly available to the research community, including but not limited to the Image Data Resource (IDR; Williams *et al*, 2017), BioImage Archive (Hartley *et al*, 2022), OpenCell (Cho *et al*, 2022), OpenOrganelle (Xu *et al*, 2021), Allen Cell Explorer (Viana *et al*, 2023), the JUMP Cell Painting consortium (Chandrasekaran *et al*, 2021), and the Human Protein Atlas (Uhlen *et al*, 2015). These platforms have improved the standards for data reporting, with more transparent datasets made available in a sustainable manner (Williams *et al*, 2017; Swedlow *et al*, 2021). However, to further consolidate reproducible microscopy research, retrieving and cross‐correlating image data accessible from different laboratories is required to reuse the data for secondary purposes and to perform meta‐analysis studies. An obstacle to this is that we so far lack guidelines and rules for implementation and reuse of high‐content imaging data from different sources and, arguably, variability in procedures. Consequently, the data variability between laboratories typically lack standardization and are not suitable for high‐quality meta‐analysis studies (Zaritsky, 2018).

Other types of complex data in the life sciences have for long been shared and extensively reused. As examples, multiple studies have addressed the reproducibility of data produced by different laboratories, for instance of mass spectrometry and RNA‐seq based data (Addona *et al*, 2009; 't Hoen *et al*, 2013; Collins *et al*, 2017; Giraldez *et al*, 2018).

We recently made efforts to standardize cell migration research (Masuzzo *et al*, 2015a, 2015b; Gonzalez‐Beltran *et al*, 2020). Here, we present a study by five laboratories, in which we quantified the sources of variability at different scales in high‐content imaging data of migrating cancer cells in 2D (three independent laboratories) and 3D (two independent laboratories) environments. Cell migration includes critical dynamic features that change over time, and thus entails a higher complexity as compared to imaging data of fixed features. Importantly, the highest technical variability occurred between laboratories, and to lower extent between persons, preventing direct high‐quality meta‐analysis of the primary data. However, in perturbation experiments, the variability could be overcome by a batch effect removal approach to achieve reliable meta‐analyses of image‐based datasets from different sources.

## Results

### 2D live cell imaging design and performance

**Figure 1:**
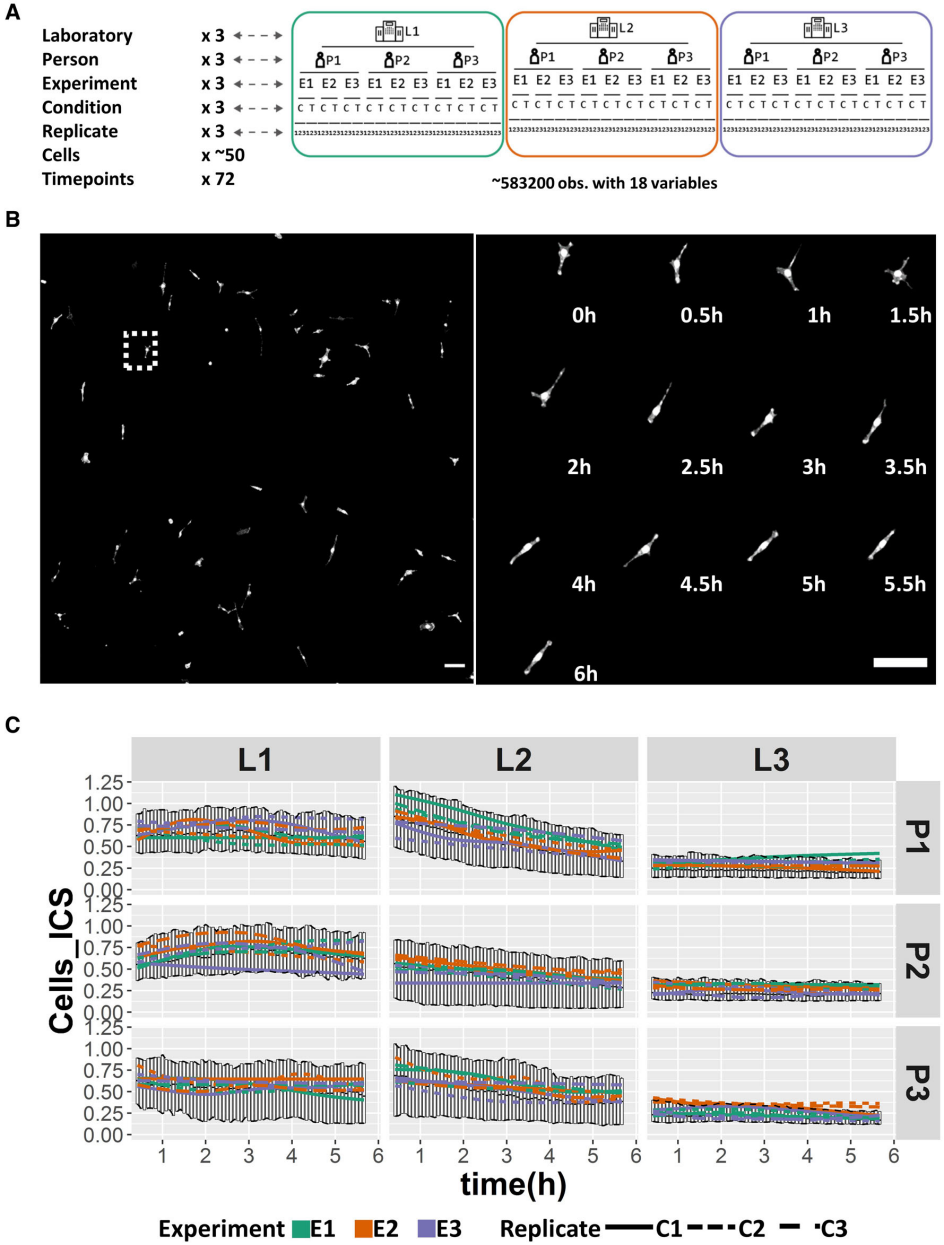
Study design and initial results Schematic of the study design. The study involved three independent laboratories, three persons in each laboratory, three independent experiments by each person, two conditions (control or ROCK inhibitor) in each experiment, and three replicates in each condition. For each replicate, around 50 cells were imaged for 6 h in 5‐min time intervals. Eighteen variables were quantified from each image series.Example of acquired time lapse images. Left: stitched large image; right: cropped images of one cell at different time points. Scale bar: 100 μm.Quantification results of Instantaneous Cell Speed (ICS) over time for each laboratory (L1‐3), person (P1–3), experiment (E1–3), and technical replicate (C1–3) in the control condition. The different colors of the lines represent the data from three different experiments. Different style of the lines with the same color represent the mean value of the data from three different technical replicates within one experiment. The error bar indicates the first and third quartiles of the data from all the three experiments at each time point.

To quantify the sources of variability, a live cell imaging design of cell migration on a 2D surface was replicated in a multilevel, nested structure. Migration behavior of HT1080 fibrosarcoma cells, stably expressing LifeAct‐mCherry and H2B‐EGFP, on a collagen‐coated glass surface was recorded using automated fluorescent light microscopes equipped with an environmental chamber. A detailed common protocol (Appendix Protocol S1, and Movies EV1 and EV2) was designed and distributed to all participating laboratories as well as the cell line and all key reagents, aiming at minimizing the biological and technical variance. The design involved three independent laboratories, three persons at each laboratory, three independent experiments by each person, two conditions (control and ROCK inhibitor) in each experiment, and three technical replicates in each condition (Fig 1A). In each technical replicate, around 50 cells were imaged in 5‐min intervals for 6 h (Fig 1B). Experiments were carried out independently by the three participating laboratories, and deviations from the original protocol were kept for the record, including independent microscopy platforms, objective specifications, control hardware for climatization of the cell cultures during microscopy, reagent differences, as well as how strictly the protocol was followed (Dataset EV1). All microscope‐derived images were transferred to the Strömblad laboratory for processing, quantification, data processing, and statistical analysis. This uniform data analysis secures identical post‐experiment data processing and allows to uncover sources of variability in the experimental procedures.

### Data description

For all image time sequences, cellular and nuclear variables were automatically extracted using CellProfiler by the same cell segmentation and tracking strategy, followed by Matlab processing to define protrusion, retraction, and short‐lived cell regions (Kowalewski *et al*, 2015) based on the CellProfiler‐derived cell masks (see Materials and Methods section for details). The raw images, CellProfiler pipeline, and Matlab scripts have been shared in the SciLifeLab Data Depository. The CellProfiler pipelines for each laboratory and the Matlab scripts are also available in GitHub (https://github.com/hujianjiang/Variability) and the raw images are also available in the BioImage Archive (https://www.ebi.ac.uk/bioimage-archive) under accession number S‐BIAD657. As a result, a total of 18 variables describing either morphological or dynamic features of the cell or the nucleus were obtained and further analyzed. Results accounted for the evolution of each variable over time, for each laboratory, person, experiment, and technical replicate (Fig 1C and Appendix Fig S1A–Q), were displayed to identify differences in the magnitude or trends of the described variables at these different levels.

**Figure 2:**
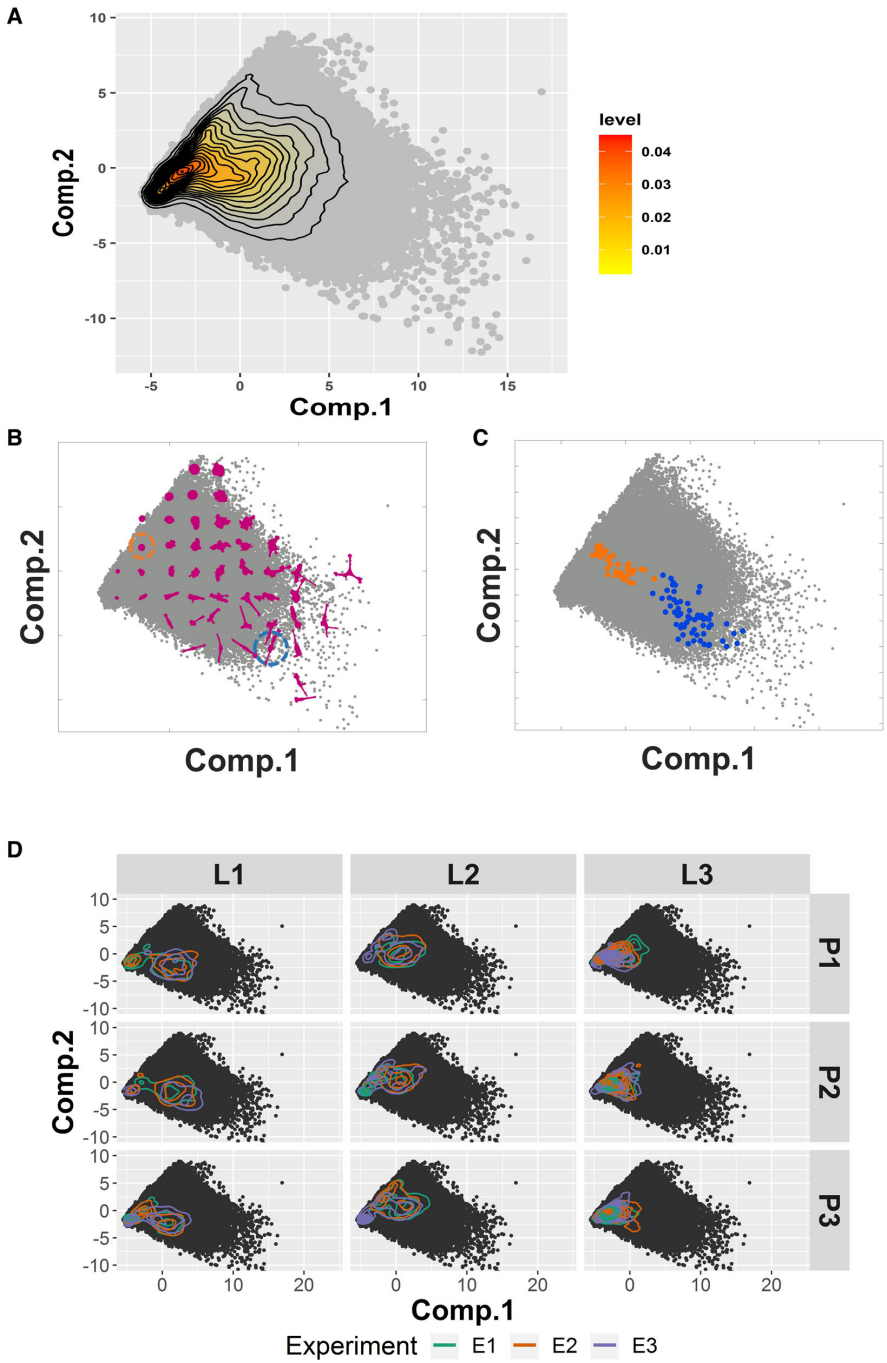
Principal component analysis of the initial results Principal component analysis results of all variables extracted from the entire data. Gray dots show the position of the first and second principal components for all of the observations from the control condition (untreated cells). Each observation is the status of one cell at one time point. Inset marks the density of the observation dots.Visualization of cell shapes at different locations of the PCA space. Gray dots show the position of the first and second principal components for each observation. Representative cell shapes at specific locations in the PCA plot are shown in magenta.The locations of the same cell at different time points within the PCA plot. Gray dots show the position of the first and second principal components for all of the observations from the control condition (untreated cells). Orange and blue dots show the locations of two different cells (dash circled in b) in the PCA space at different time points.Principal component analysis results shown for each person (P) in each laboratory (L). Black dots show the position of the first and second principal components for all of the observations from the control condition (untreated cells). Each observation is the status of one cell at one time point. Colored lines show the 2D density plots of the technical replicates, where lines with different colors in the same plot represent different experiments. The principal component space is identical in all the plots.

Z‐score standardization was applied to all features, and subsequent principal component analysis (PCA) was performed in order to maximize and visualize the variability. The first two principal components represent > 60% of the variability in the observations (Appendix Fig S2A and B). By combining all observations, we found that the data concentrate around the mean value and dissipate progressively from there, without apparent differentiated clustering of observations in the PCA space (Fig 2A; Gordonov *et al*, 2016). Observations with different cell shape or the same cell at different time points locate at different places of the PCA space (Fig 2B and C). Differences in data localization, variability, and clustering were detectable by 2D principal component analysis representing variations among technical repeats, experiments, persons, or laboratories (Fig 2D and Appendix Fig S3A–C).

### Variability sources

We have used a Linear Mixed Effects (LME) model to quantify the variability within each hierarchical level of the experiment structure and to compare the variability across these levels. Linear Mixed Effects is a linear regression‐based statistical method to analyze data by simultaneously modeling both fixed effects (in this case hierarchical levels) and random effects (in this case variation within each level) (Goldstein, 1986; Bates *et al*, 2015; Hox *et al*, 2017).

**Figure 3:**
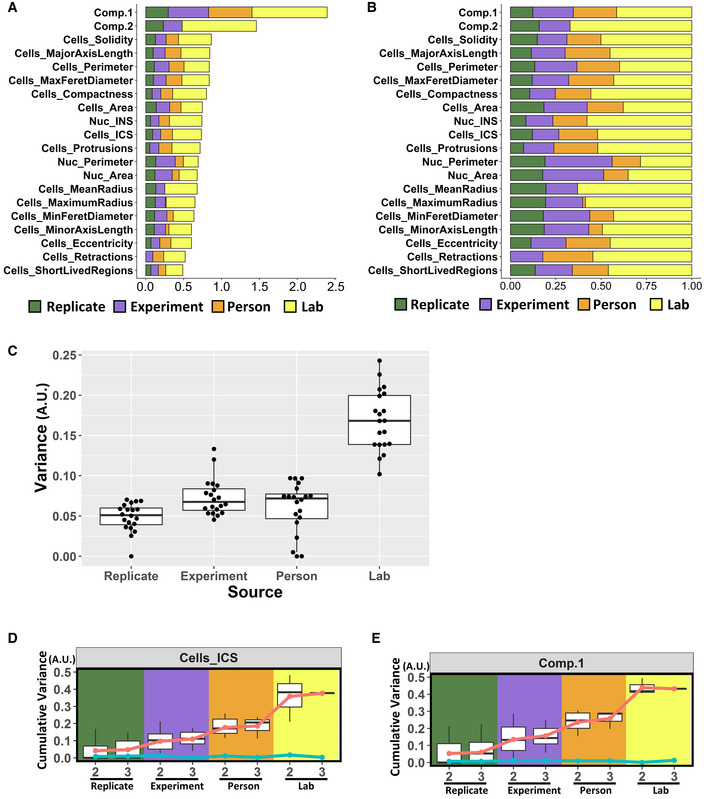
Lab‐to‐lab variance contributes the most to the technical variance A, BVariance components of each variable from all technical levels based on the Linear Mixed Effect (LME) model analysis. (A) absolute value; (B) relative value.CBoxplot of the absolute variance components of all the variables from technical replicate, experiment, person, and laboratory levels based on the LME model analysis. Each dot represents one variable within the corresponding variance level. All of the 18 variables are plotted at each level.D, ECumulative variability of Instantaneous Cell Speed (ICS) (D) and first principal component (E) at the levels of technical replicate, experiment, person, and laboratory. Boxplots show variances with two or three replicates, experiments, persons, or laboratories, calculated at each level. Red dots show the mean value of the cumulative variance that are linked with red lines. As a control, cyan dots and lines show the cumulative variance of the same data after randomization. Data information: For the boxplots in (C–E), in each box, the central mark indicates the median, and the bottom and top edges of the box indicate the first quartile and third quartile, respectively. The whiskers extend to the most extreme data points not considered outliers. The data between the first quartile −1.5*interquartile range and third quartile +1.5*interquartile range are considered not outliers.

Our LME modeling included a fixed intercept parameter and no independent variables. It also contained a hierarchical structure of nested levels of random intercepts. The random intercepts were assumed to follow a normal distribution. The estimation of all the parameters in the model was based on the maximization of the likelihood function. We applied the model to the 18 considered variables and to the first and second principal components. From the model, we obtained the variance components at each of the levels (temporal, cell, technical replicate, experiment, person, and laboratory) for each variable (Appendix Fig S4A and B) and categorized the sources of variability as biological or technical variability. Biological variability originated from the cell identity (cells in a population display variability for a given variable) and temporal variation (the same cell displays variability for a given variable when studied at different time points). Technical variability originated from the technical replicate, experiment, person, and laboratory. There was substantial biological variability within the cell population and for each cell over time (Appendix Fig S4A–C), in part likely due to the existence of distinct cell migration modes within a cell population (Shafqat‐Abbasi *et al*, 2016). By aggregating the variabilities, we identified technical sources to contribute 32% (median value) of the total variance across all variables (Appendix Fig S4D). While proper study design in terms of the sample size (number of cells, etc.) should take the inherent biological variability into account to facilitate the detection of statistically discernable differences, the reproducibility of the data is defined by their technical variability. Importantly, among the technical variability, lab‐to‐lab variability was found to be the major source, followed by person, experiment and replicate, but with different relative contributions among different variables (Fig 3A–C).

We then determined the source of technical variability in more detail at each level. We computed the cumulative variability deriving from technical sources when adding additional levels to a hypothetical experimental design with increasing complexity (Materials and Methods and Appendix Table S1). For this, based on all the possible subdatasets that fulfilled the specified criteria ensuring dataset integrity, we observed a relatively smooth increase in variability due to technical sources that progressed with increased number of technical replicates, experiments, and persons. However, importantly, the cumulative variability was almost doubled when data from two laboratories were combined. Adding a third laboratory to the dataset did not substantially increase the cumulative variability (Fig 3D and E, and Appendix Fig S5).

### Batch effect removal

Inspired by the extensive research in RNA‐seq and image‐based drug screen experimental designs to measure and correct for batch effects ('t Hoen *et al*, 2013; Giraldez *et al*, 2018; Chandrasekaran *et al*, 2021), we applied a similar approach to our study to curate the variability. For this, the LME model was computed using the complete dataset (both control and ROCK inhibition conditions), keeping the same random effects as previously used and including the control or ROCK inhibition as fixed effect.

**Figure 4:**
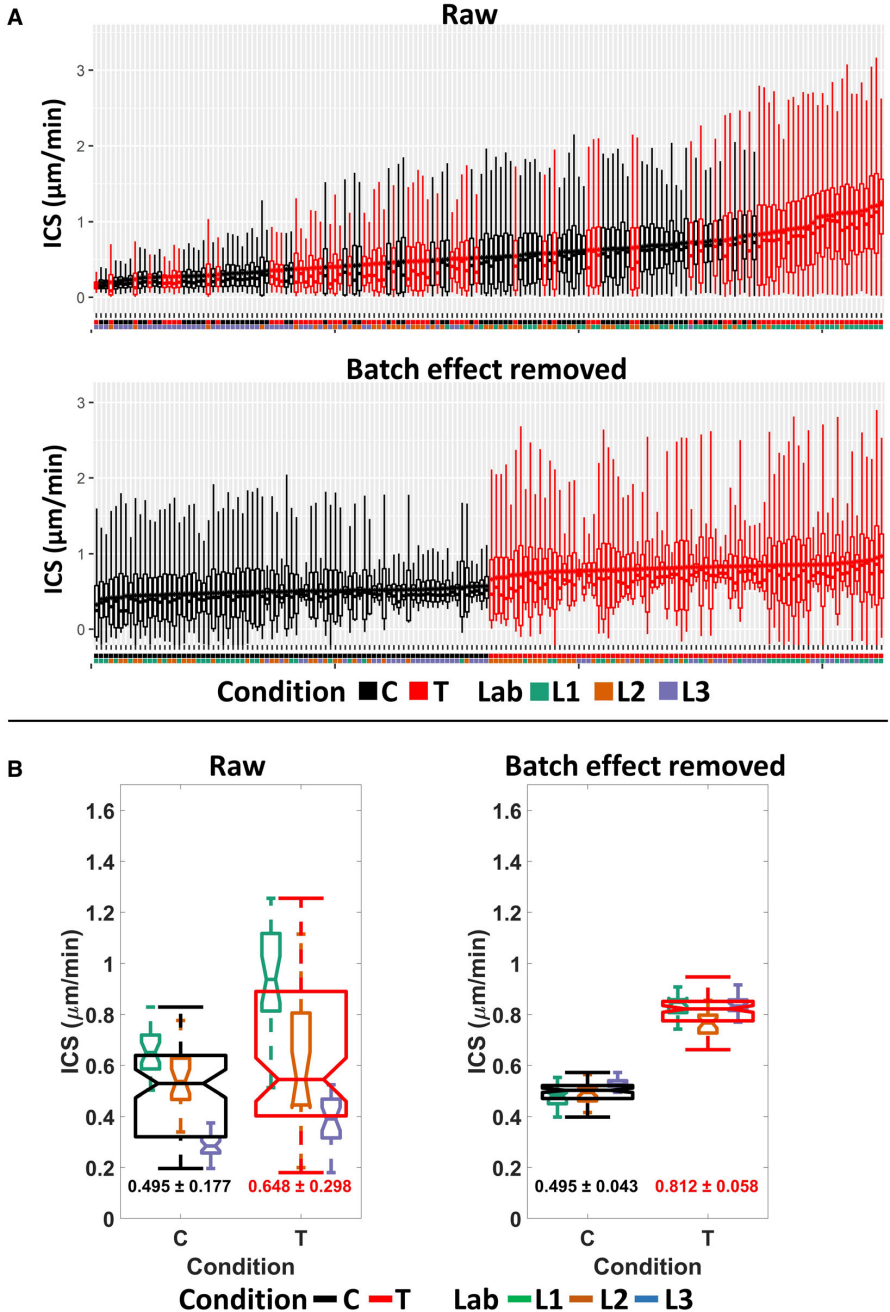
Batch effect removal dramatically reduces the variance of Instantaneous Cell Speed (ICS) ICS distribution before (top) and after (bottom) batch effect removal on control (C – black) and perturbed (ROCK inhibition; T – red). Boxplots display ICS observations for each replicate, sorted by increasing value of the mean. Control and perturbation conditions are shown in black and red respectively. Laboratories in which each replicate was performed are color coded below the boxplots. Each replicate includes results from ~50 cells, and each cell has results from 72 time points.ICS values and variance before (left) and after (right) batch effect removal. Boxplots are based on mean ICS of each technical replicate from control and perturbed conditions in different laboratories. Laboratories are color coded, while the aggregate results from all labs are shown in black (control) and red (perturbed). The numbers below the corresponding boxplot show mean ± standard deviation of the aggregated control/treated results from all labs. The experiments were repeated in three labs, three persons in each lab, three experiments by each person, and three technical replicates in each experiment. Data information: For the boxplots in (A and B), in each box, the central mark indicates the median, and the bottom and top edges of the box indicate the first quartile and third quartile, respectively. The whiskers extend to the most extreme data points not considered outliers. The data between the first quartile −1.5*interquartile range and third quartile +1.5*interquartile range are considered not outliers.

**Figure 5:**
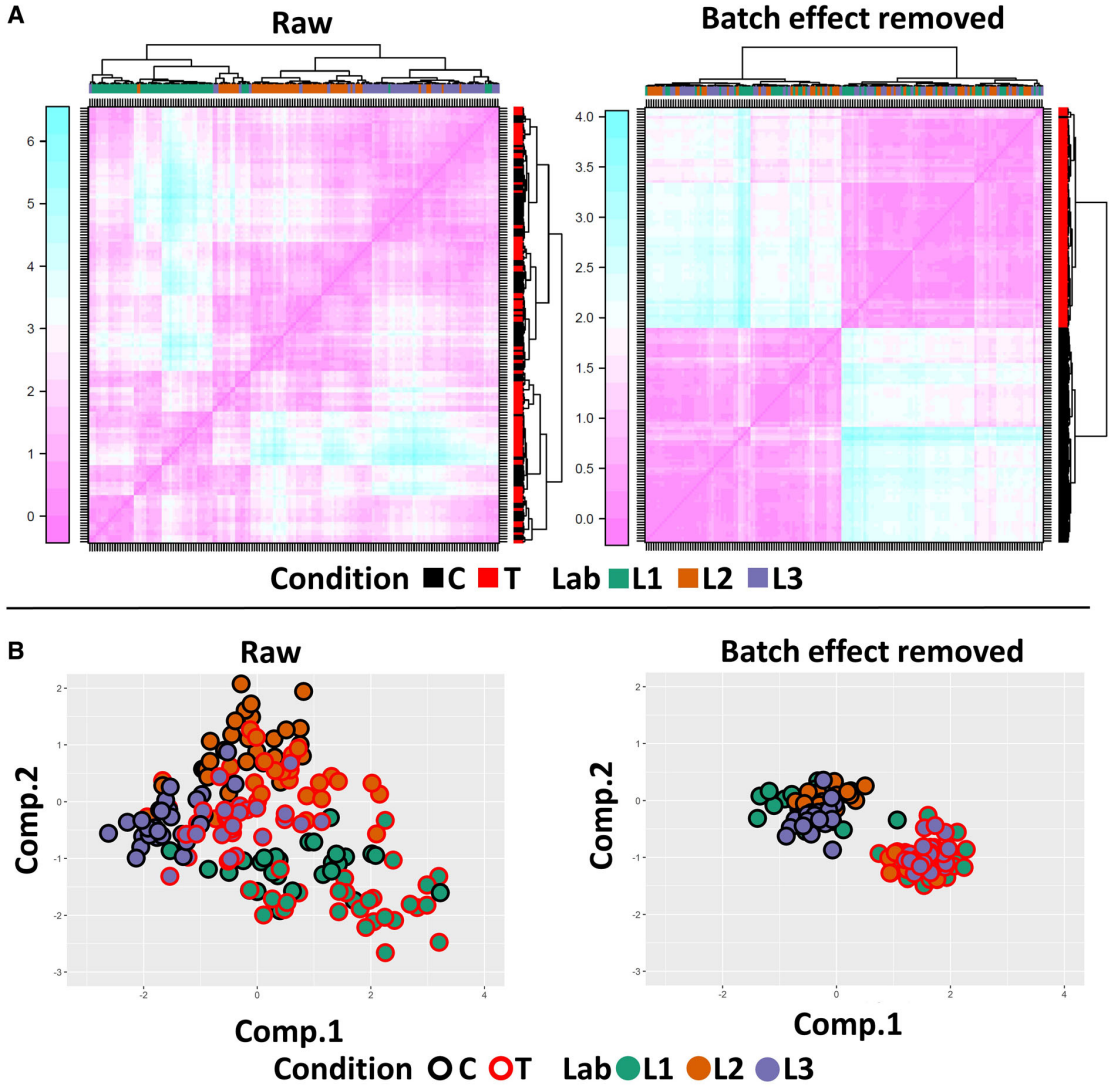
Batch effect removal dramatically reduces the variance of principal component data of 2D cell migration data Heatmap of the distance matrix before and after batch effect removal at technical replicate level. The heatmaps show average values of the distance matrix between 1^st^ and 2^nd^ Principal Components per lab, person, experiment, condition, and technical replicate before (left) and after (right) batch effect removal. Each row/column corresponds to one technical replicate. Sorting based on hierarchical clustering.Batch effect removal in principal component data of 2D cell migration data at the technical replicate level. Technical replicate of first and second Principal Component average values before (left) and after (right) batch effect removal are shown in the same PCA space. Each dot represents one technical replicate. Results from different laboratories/conditions are color coded as indicated.

We conducted this approach to the Instantaneous Cell Speed (ICS, Fig 4A) and to the first and second Principal Components of all variables (Fig 5A). For each observation, we computed and discriminated the effects derived either from random effects (derived from the laboratory, person, experiment, or technical replicate) or from the fixed effect (ROCK inhibition; Shafqat‐Abbasi *et al*, 2016). The results clearly show that this approach allows for an unambiguous discrimination between the control and treatment conditions, therefore showing that the experimental variability in cell migration experiments can be addressed in order to better discriminate the effect of a given perturbation (Figs 4A and B, and 5A and B, Appendix Fig S6A–D). The batch‐effect‐removed data showed a robust increase in ICS as a result of the perturbation in the data from all three laboratories. In comparison, only laboratory #1 produced a similar‐sized increase as without batch effect removal, while the other laboratories displayed small differences. Thus, the direct comparison of data from cell migration experiments among our laboratories, each highly experienced in cell migration designs and experiments, could lead to discordant conclusions on the perturbation effect. The variance of the data generated in the same laboratory by different persons could also be reduced with the batch effect removal strategy (Appendix Figs S7A–C, S8A–C and S9A–C). This highlights the importance for image‐based quantitative studies of statistical methods for batch effect removal, which have recently been implemented in image‐based screening (Chandrasekaran *et al*, 2021).

### Validation of batch effect removal in 3D cell migration

**Figure 6:**
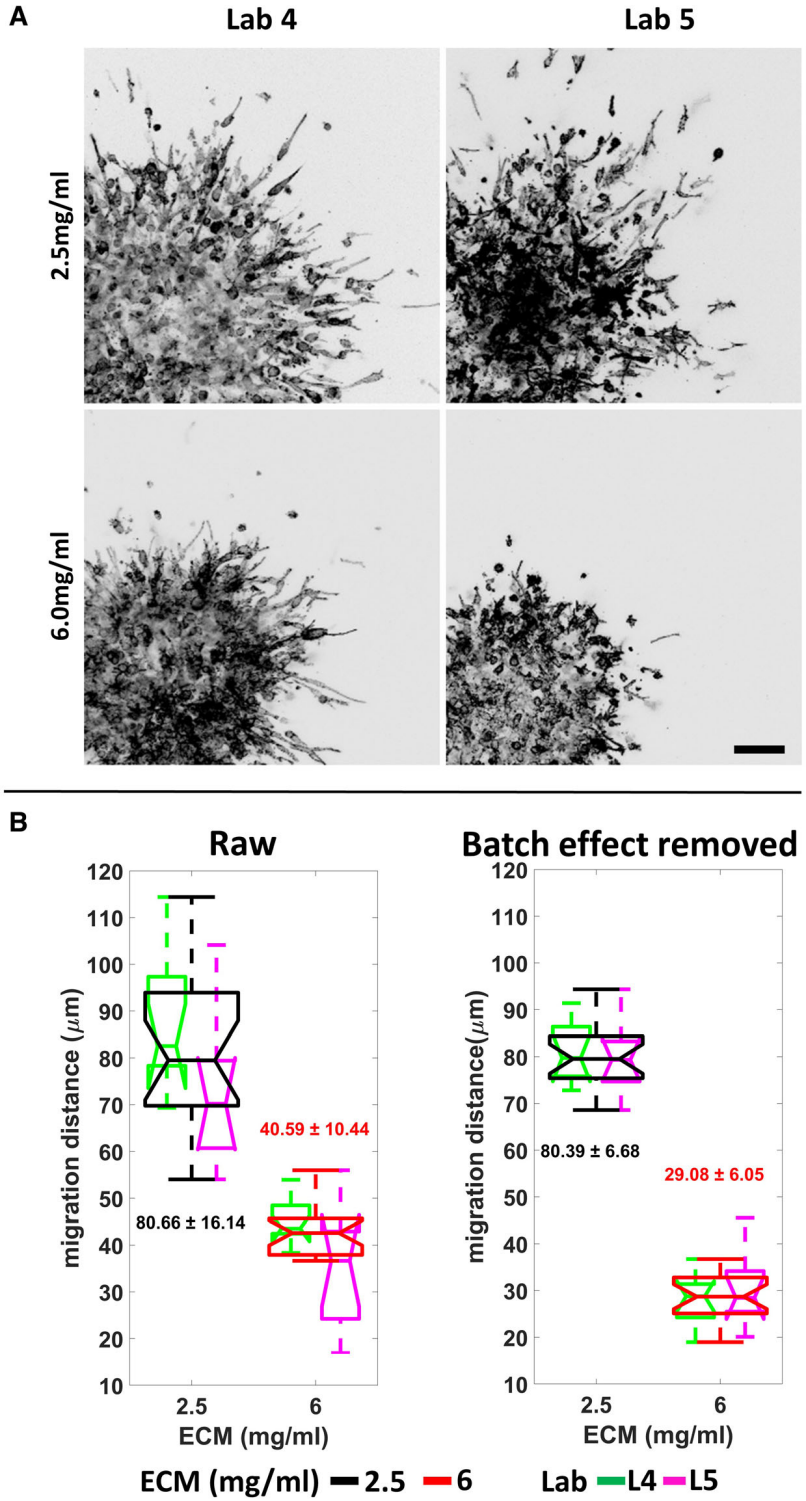
Batch effect removal dramatically reduces the variance of the 3D cell migration data Sample images of the produced 3D cell migration datasets by Lab 4 and Lab 5. HT1080 cells were seeded in the collagen condition 2.5 vs. 6.0 mg/ml. Bar: 100 μm.Batch effect removal in 3D cell migration (3D spheroid invasion) data. Boxplots are based on the mean 3D cell migration distance of the technical replicates of the HT1080 cells embedded in different concentrations of collagen before (left) and after (right) batch effect removal. Different ECM concentrations are shown in black (2.5 mg/ml) or red (6 mg/ml) and data from different laboratories are indicated with green (Laboratory #4) and magenta (Laboratory #5). The aggregated 2.5 or 6 mg/ml results from both laboratories are shown with the corresponding boxplots. In each laboratory, the experiments were repeated three times with three technical replicates in each experiment. Each technical replicate contains at least three different spheroids. For the boxplots, in each box, the central mark indicates the median, and the bottom and top edges of the box indicate the first quartile and third quartile, respectively. The whiskers extend to the most extreme data points not considered outliers. Data between the first quartile −1.5*interquartile range and third quartile +1.5*interquartile range are considered not outliers.

We also applied the batch effect removal approach to a 3D cell migration dataset generated from two independent laboratories with a similar strategy as for the 2D cell migration experiment (Appendix Fig S10A–C and Fig 6A). The difference of migration distance of the cells in response to low‐ (2.5 mg/ml) or high‐density (6 mg/ml) concentration of polymerized 3D fibrillar collagen was already reliably discriminated comparing the raw data (Appendix Fig S11A), as previously described (Wolf *et al*, 2013). However, significant lab‐to‐lab variance of results within each test group was still observed (Fig 6B left). Also in this case, the batch effect removal processing significantly reduced the variance and provided a more robust detection of increased migration distance in lower‐density 3D collagen (Fig 6B right and Appendix Fig S11B).

## Discussion

The emerging increase in high‐content imaging data sharing provides opportunities for data reuse and meta‐analysis. For example, the BioImage Archive stores and distributes biological images from any imaging modality (Hartley *et al*, 2022) and the IDR combines data from multiple resources and integrates them into a single resource for reanalysis in a scalable form (Williams *et al*, 2017). However, the usefulness of these opportunities remains largely untested, and the sources of variance within this type of data need to be characterized. In this study, we found that variation between laboratories is the major source of technical variance in high‐content imaging data of cell morphology and migration features. This outcome suggests that, although the experimental design was idealized including sharing of a detailed protocol, cells and reagents, standardizing details such as cell passaging prior to the experiment, cell density prior to seeding for migration, the type of fetal bovine serum, and cumulative passage number of cells, the lab‐to‐lab variance currently limits the value of meta‐analysis of the basic high‐content cell image data. This lab‐to‐lab variance may at least in part be explained by observed local variations in equipment and practices, including use of different microscopes and their differences in what imaging plates could be harbored, and lab‐to‐lab differences of cell density apparent in the images, despite that the same standard method was used for cell quantification.

Importantly, however, we show that application of a batch effect removal approach significantly reduced the technical variance at all levels and provided useful meta‐analysis of perturbation effects in both 2D and 3D spheroid culture models performed in different laboratories, at least under our highly standardized conditions. Similar batch effect removal approaches have been important for meta‐analysis in other fields and data types, such as from RNA‐sequencing and peptide‐centered proteomics via mass spectrometry (Leek *et al*, 2010; Gregori *et al*, 2012; Tran *et al*, 2020). The batch effect removal strategy has also started to be applied in image‐based screening (Chandrasekaran *et al*, 2021; Walton *et al*, 2022). However, no standard batch effect method has yet been widely established for image analysis. The LME‐model‐based batch effect removal performed well in this study, but was not compared with alternative statistical methods. Further studies would be useful that focus on the applicability of different batch effect removal methods on different types of real‐world imaging‐based data.

The present study, although it entailed a high degree of standardization, indicates that the usefulness of high‐content image data meta‐analysis is currently limited to the study of perturbation effects, and for which batch effect removal is necessary. Further studies are therefore needed to define the usefulness of meta‐analysis using different cell models and perturbations and in particular upon use of more typical high‐content image datasets that are more loosely standardized than ours and that often differ not only in their precise design, but also in study purpose and aim.

## Materials and Methods

### Reagents and Tools table

**Table:** 

Reagent/Resource	Reference or Source	Identifier or Catalog Number
**Experimental Models**		
HT1080 cells (*H. sapiens*)	DSMZ Braunschweig	ACC315
HT1080 cells stably expressing Lifeact‐mCherry & H2B‐EGFP (*H. sapiens*)	Friedl Lab	N/A
**Antibodies**		
Primary antibody YAP, rabbit mAb IgG (D8H1X)	Cell Signaling Technology	#14074
Isotype control, rabbit mAb IgG (DA1E)	Cell Signaling Technology	#3900
Goat‐anti‐rabbit IgG1 AlexaFluor488	Thermo Fischer Scientific	A11034
**Chemicals, Enzymes and other reagents**		
High glucose DMEM	Gibco	41965‐039
FBS	Gibco	10270‐106
Sodium pyruvate	Gibco	11360070
Penicillin/streptomycin	Gibco	15140‐122
DMSO	Sigma	D2640
Trypsin (10×)	Life Technology	15400‐054
T‐75 flask	SARSTEDT	83.3911.002
T‐25 flask	SARSTEDT	83.3910.002
18G syringe needle	KDM	900444
6‐well plate	FALCON	353046
96‐Imaging Plate CG (Cover Glass)	Mo Bi Tec	5241‐20
Collagen I	Corning	354249
Heat denatured 0.5% BSA	Sigma	A2153
Y27632 (ROCK inhibitor)	BD	562822
DAPI	Sigma	D9542
AlexaFluor633‐Phalloidin	Molecular Probes	A22284
Bovine Serum Albumin (BSA), Fraction V	biomol	1400
Purecol Bovine Collagen Solution (Type I)	Cell Systems	5005‐100ML
Methyl cellulose	Sigma	M6385
DMEM (Dulbecco Modified Eagle Medium, High Glucose)	Gibco/Thermo Fischer Scientific	10938‐025
Fetal calf serum (FCS)	Sigma	F7524
EDTA	Invitrogen/Thermo Fischer Scientific	15575020
15‐cm culture dish	Greiner Bio‐one	639160
96‐well imaging plates (μ‐Plate 96 Well, cell culture surface coating, sterile)	Thermo Scientific NUNC	#165305
**Software**		
MATLAB	Mathworks	
CellProfiler (v2.2.0)	https://cellprofiler.org/	
ImageJ	https://imagej.nih.gov/ij/index.html	
Nucleus Annotation 3D (NA) plugin	https://github.com/Mverp/Nucleus‐Annotation‐3D	
Cell Migration Analyser 3D (CMA) plugin	https://github.com/Mverp/Cell3DMeasurements	
R (v4.1.2)	https://www.r‐project.org/	
**Other**		
Nikon A1 plus confocal microscope	Nikon	
Wide field Delta Vision microscope	Delta Vision	
Zeiss LSM780 confocal microscope	Zeiss	
Zeiss LSM880	Zeiss	

### Methods and Protocols

#### Cell culture and imaging

##### 2D cell migration

We developed highly detailed protocols for cell culture and seeding for live cell imaging that was shared and used for all experiments (see Appendix Protocol S1). Briefly, the Friedl laboratory provided HT1080 cells stably expressing LifeAct‐mCherry and H2B‐EGFP (before sharing, the Sahai laboratory performed the standard cell authentication procedure on this cell line by comparing its STR profile to the public database). Mycoplasma infection was excluded prior to the experiments. Cells were cultured with high glucose DMEM supplemented with FBS (10%), sodium pyruvate (1 mM), and penicillin/streptomycin (100 U/ml). Cells were passaged at ~80–90% confluence, up to passage number 20. One day before imaging, 2 × 10^5^ cells were seeded onto one well of the six‐well plates and left overnight in the incubator. On the experimental day, the assay wells were prepared as follows:

1. 100 μl of 20 μg/ml collagen I was added to each of six wells in a 96‐well imaging plate or chambered coverslip and incubated at 37°C for 2 h.
2. The supernatant was discarded by flipping the plate upside down and subsequently, each well was incubated at 37°C for 20 min with 100 μl of heat denatured 0.5% BSA for blocking.
3. 500 cells in 100 μl of serum‐free culture medium were seeded in each of the six individual wells of the 96‐well imaging plate or chambered coverslip, ensuring homogeneous cell distribution by tapping the plate or chambered coverslips in perpendicular directions.
4. After 10 min, during which cells attached to the well bottom, the imaging plate was incubated at 37°C and 5% CO_2_ for 2.5 h.

For the live cell imaging, we used multidimensional automatized microscopes with an environmental chamber to keep temperature, humidity, and CO_2_ constant. Prewarmed media with or without ROCK inhibitor (Y27632, final concentration at 15 μM) was added before the start of imaging. A 20× 0.75 NA objective was used and tiled images (5 × 5) were generated to capture a large area in each well. The images were acquired in 5‐min interval for 6 h.

The detailed protocol is attached in the Appendix Protocol S1 and Movies EV1 and EV2. Any deviations from the distributed procedure were recorded and summarized (Dataset EV1).

##### 3D spheroid invasion assay (3D cell migration experiment)

We developed a detailed workflow for a 3D spheroid invasion assay that was shared and used for all experiments performed at two different locations. For detailed protocols for 3D spheroid culture and labeling, imaging, and image analysis, see Appendix Protocols S2–S4, respectively.

###### 3D spheroid culture and labeling

Briefly, the Friedl laboratory provided HT1080 cells. Before sharing among the two groups, the Sahai laboratory validated this cell line by comparing its STR profile to the published ones. Mycoplasma infection was excluded prior to the experiments. Cells were cultured in T75 flask with 10% CO_2_ at 37°C. 3D spheroid culture and labeling were performed as follows:

1. Multicellular spheroids containing 1,000 HT1080 cells were generated using hanging‐drop culture method (Del Duca *et al*, 2004).
2. The spheroids were embedded in rat‐tail collagen I (Corning, Cat no. 354249), in up to 18 wells of 96‐well imaging plates per collagen concentration, using 1 spheroid per gel and a final collagen concentration of 2.5 or 6 mg/ml. Former protocols for spheroid embedding (Wolf *et al*, 2013; Haeger *et al*, 2014) were adapted to have control over the number of spheroids per well, spheroid height with respect to imaging window and the onset of collagen polymerization, to minimize variation between technical repeats per plate.
3. Plates were incubated for 24 h at 37°C to establish cancer cell invasion in three dimensions, prior to fixation in 4% PFA.
4. The 3D cell cultures were fluorescently stained with DAPI (Sigma, D9542, 2 μg/ml) and AlexaFluor633‐Phalloidin (Molecular Probes, A22284, 1:200 dilution) and stored (preferably for < 48 h) at 4°C prior to imaging.

###### 3D spheroid microscopy imaging

1. The lower left corner of the spheroid was positioned in the scan field, with the border of the spheroid core touching the image border (see details in Appendix Protocol S3).
2. A z‐range of up to 120 μm was used to image from z = 1/2 to z = 4/5 of spheroid dimensions.
3. Transmission, reflection and fluorescence channels were recorded sequentially at 8‐bit resolution. The laser power was set close to the saturation limit of the dye. The detector amplification (high voltage) was set in such a manner, that the brightest cells in migration zones made use of the full digital detection range.
4. In both laboratories, imaging was performed using a Zeiss LSM880 equipped with a 20× 0.8 NA objective. The following microscope parameters were used: scan field 708.5 μm^2^, pixel size 1.2 μm, pixel dwell time 1.3 μs, z‐step size 2 μm and line averaging 3.

The metadata of the 2D and 3D images were also recorded and saved based on the Minimum Information About Cell Migration Experiments (MIACME) reporting guideline, which is being developed by the Cell Migration Standardization Organization (CMSO; Gonzalez‐Beltran *et al*, 2020). The metadata are available together with the raw images at the SciLifeLab Data Repository (https://doi.org/10.17044/scilifelab.21407402) and the BioImage Archive (https://www.ebi.ac.uk/bioimage-archive) under accession number S‐BIAD657.

#### Image processing

##### 2D live cell imaging data

1. Images were converted from the original format to .tif format.
2. To generate large image composites, stitching was performed either automatically during acquisition or via a custom‐made MATLAB script.
3. Images from different laboratories were resized to the same resolution (0.8260 μm/pixel), to allow proper further comparison.
4. A CellProfiler (v 2.2.0) pipeline was used to automatically segment and track cells and nuclei, and to extract 15 morphological and dynamic variables from the raw images (shown in Fig 3A). Because images from different laboratories were acquired with different types of microscopes, Gaussian noise with a mean of zero and a standard deviation of 0.00001 was added to the wide field images from Laboratory 2 before the cytoplasm segmentation to equalize the segmentation across laboratories as much as possible. All the other CellProfiler pipelines and parameters were the same across all the three laboratories. The nuclei were identified as primary objects using a global background thresholding method, while the corresponding cytoplasm was identified as the secondary object with an adaptive robust background thresholding strategy and a watershed method to separate touching cells. The identified cells were tracked based on the distances of nuclei between time points. All the parameter details could be found in the shared CellProfiler pipelines in the SciLifeLab Data Repository and in GitHub (see specifics below).
5. In order to identify protrusions, retractions, and short‐lived cell regions, we compared consecutive, segmented cell images from the CellProfiler analysis results using tailored Matlab scripts. Protrusions were identified as regions present in a cell at a certain time point but absent in the previous. Retractions were defined as regions present at one time point but absent in the next time point. Short‐lived regions are those regions that are present at only one time point but not in the ones directly before or after, corresponding to a lifetime of < 10 min (Kowalewski *et al*, 2015).

The CellProfiler pipelines for each laboratory and the Matlab scripts are available together with the raw images at the SciLifeLab Data Repository (https://doi.org/10.17044/scilifelab.21407402). The CellProfiler pipelines for each laboratory and the Matlab scripts are also available in GitHub (https://github.com/hujianjiang/Variability) and the raw images are also available in the BioImage Archive (https://www.ebi.ac.uk/bioimage-archive) under accession number S‐BIAD657.

##### 3D spheroid invasion data

This workflow was implemented in Fiji as the Nucleus Annotation 3D (NA) and the Cell Migration Analyser 3D (CMA) plugin sets (https://github.com/Mverp/Nucleus‐Annotation‐3D and https://github.com/Mverp/Cell3DMeasurements) and was distributed to two independent laboratories (RUMC and CRICK) for standardized analysis of independent datasets from spheroid culture performed in each laboratory independently (Appendix Protocol S4).

1. First, the outline of the spheroid core was defined by manually setting four points far away from each other, in the 3D image stack to be analyzed, at the spheroid border.
2. Based on the four points, the annotation program defined a sphere in the dataset, which was used as a reference for migration distance from the spheroid core.
3. Then, the DAPI channel of the 3D image datasets was used for nuclear segmentation, segments at the border were removed and the distance of the center of each nucleus to the defined spheroid core was quantified and recorded for subsequent analysis. To optimize and validate the plugin, segmentation outputs were compared with manually annotated “gold standard” images.
4. After segmentation, the nuclei occurring in both annotation and segmentation output, the true positives, were automatically calculated.
5. Next, the performance of the segmentation was quantified by calculating the precision (# true positives / # nuclei in segmentation) and recall (# true positives/# nuclei in the annotation).

The analysis was performed on the image data of both laboratories using the optimized settings (MigrationAnalysisParameters.txt, included in the SciLifeLab Data Repository https://doi.org/10.17044/scilifelab.21407402 and GitHub https://github.com/hujianjiang/Variability).

#### Data processing and statistical modeling of the 2D cell migration data

Preprocessing: Based on the original tracking data, the static and rounded cells were excluded based on visual assessment. Then, the duplicated and merged cell/nuclear trajectories were identified and removed. Excessively large (cell area > third quantile +1.5*interquantile range of the areas from all cells) or small (nuclear area < 100 μm^2^) cells were excluded based on the measurements of cellular and nuclear area, in order to remove noise from cell debris and cell aggregations. The remaining trajectories were smoothed with the rolling window method with window size of 9. The Instantaneous Cell Speed (ICS) was calculated based on the smoothed trajectories.

We used a linear mixed effects model to estimate the mean value of the input (instantaneous cell speed, first principal component, second principal component, migration distance, and all the other 16 parameters) across levels of laboratories, persons, experiments, technical replicates, and cells. The numeric independent variable in the model was the input. The model included a fixed intercept parameter and no independent variables. It also contained a hierarchical structure of five nested levels of random intercepts. The nested levels corresponded to, in order, laboratories, persons, experiments, technical replicates, and cells. The random intercepts were assumed to follow a normal distribution. We used the variance of the inner‐level residual as measure of the temporal variability. The estimation of all the parameters in the model was based on the maximization of the likelihood function. The linear mixed effect modeling was performed based on the R package *lme4* (Goldstein, 1986; Bates *et al*, 2015; Hox *et al*, 2017).

For the cumulative variability calculation, we designed a hypothetical experimental design with increasing levels of complexity: two or three replicates, two or three experiments with three replicates each, two or three persons performing three experiments with three replicates each, or two or three laboratories where three persons perform three experiments with three replicates. For this, we generated all the possible subdatasets that fulfilled the specified criteria ensuring dataset consistency (this is, we avoided the combination of data that did not have the same origin in the higher level of the hierarchical structure), and computed the cumulative variability for each level. Appendix Table S1 shows how the datasets were generated. As a control, we first randomized the original data and then generated similar subdatasets as the original ones and calculated the cumulative variability in the same way.

#### Batch effect removal

After fitting the original data with linear mixed effect model to extract the fixed and random effects, each single observation was modified by subtracting the intercept from all levels (laboratory, person, experiment, technical replicate, and observation) and adding the fixed effect between two conditions (control vs. perturbation in 2D migration; 2.5 mg/ml vs. 6 mg/ml collagen in 3D invasion).

## Author contributions

**Jianjiang Hu:** Conceptualization; software; formal analysis; validation; investigation; visualization; methodology; writing – original draft; project administration; writing – review and editing. **Xavier Serra‐Picamal:** Conceptualization; data curation; software; formal analysis; investigation; visualization; methodology; writing – original draft; project administration; writing – review and editing. **Gert‐Jan Bakker:** Software; investigation; visualization; methodology; writing – review and editing. **Marleen Van Troys:** Software; investigation; methodology; writing – review and editing. **Sabina Winograd‐Katz:** Investigation; writing – review and editing. **Nil Ege:** Investigation; writing – review and editing. **Xiaowei Gong:** Investigation; writing – review and editing. **Yuliia Didan:** Investigation; writing – review and editing. **Inna Grosheva:** Investigation; writing – review and editing. **Omer Polansky:** Investigation; writing – review and editing. **Karima Bakkali:** Investigation; writing – review and editing. **Evelien Van Hamme:** Investigation; writing – review and editing. **Merijn van Erp:** Software; methodology; writing – review and editing. **Manon Vullings:** Investigation; writing – review and editing. **Felix Weiss:** Software; methodology; writing – review and editing. **Jarama Clucas:** Investigation; writing – review and editing. **Anna M Dowbaj:** Investigation; writing – review and editing. **Erik Sahai:** Resources; project administration; writing – review and editing. **Christophe Ampe:** Resources; project administration; writing – review and editing. **Benjamin Geiger:** Resources; project administration; writing – review and editing. **Peter Friedl:** Resources; methodology; writing – original draft; writing – review and editing. **Matteo Bottai:** Software; supervision; writing – review and editing. **Staffan Strömblad:** Conceptualization; Resources; supervision; writing – original draft; project administration; writing – review and editing.

## Disclosure and competing interests statement

The authors declare that they have no conflict of interest.

## Supporting information



Appendix

Movie EV1

Movie EV2

Dataset EV1

## Data Availability

The image and metadata are available in the BioImage Archive (http://www.ebi.ac.uk/bioimage‐archive) under accession number S‐BIAD657. The computer codes and pipelines used for image and data analysis are available in GitHub (https://github.com/hujianjiang/Variability). The datasets and computer code produced in this study are also available in the SciLifeLab Data Repository (https://doi.org/10.17044/scilifelab.21407402).
