# Supplementary material for: Multisite assessment of reproducibility in high‐content cell migration imaging data
Source: Mol Syst Biol. 2023 Apr 17;19(6):e11490. doi: 10.15252/msb.202211490 (PMC10258559; doi:10.15252/msb.202211490)
Supplement: Supplementary file 1 — Appendix [file MSB-19-e11490-s001.pdf]

Appendix Figures, Table, and Protocols

Multi-site assessment of reproducibility in high-content cell migration imaging data

Jianjiang Hu[1,8], Xavier Serra-Picamal[1,8], Gert-Jan Bakker[2], Marleen Van Troys[3], Sabina Winograd-katz[4], Nil Ege[5], Xiaowei Gong[1], Yuliia Didan[1], Inna Grosheva[4], Omer Polansky[4], Karima Bakkali[3], Evelien Van Hamme[6], Merijn van Erp[2], Manon Vullings[2], Felix Weiss[2], Jarama Clucas[5], Anna M. Dowbaj[5], Erik Sahai[5], Christophe Ampe[3], Benjamin Geiger[4], Peter Friedl[2], Matteo Bottai[7], Staffan Strömblad[1,9]

1. Department of Biosciences and Nutrition, Karolinska Institutet, Stockholm, Sweden

2. Department of Medical BioSciences, Radboud University Medical Center, Nijmegen, The Netherlands

3. Department of Biomolecular Medicine, Ghent University, Ghent, Belgium

4. Department of Immunology and Regenerative Biology, Weizmann Institute of Science, Rehovot, Israel

5. The Francis Crick Institute, London, United Kingdom

6. Bio Imaging Core, VIB Center for Inflammation Research, Ghent, Belgium

7. Division of Biostatistics, Institute of Environmental Medicine, Karolinska Institutet, Stockholm, Sweden

8. Equal contribution

9. Corresponding author

Table of contents

Appendix Figure S1 Quantification of variables over time at different levels of data hierarchy.... 3

Appendix Figure S2 Overview of the Principal Component Analysis (PCA) results from the 18 variables used in the study 9

Appendix Figure S3 Principal component analysis results shown for each experiment from each person in three laboratories 10

Appendix Figure S4 Variance components of each variable from all data from the control condition hierarchical levels based on the Linear Mixed Effect (LME) model analysis 12

Appendix Figure S5 Cumulative variance of the different hierarchical data levels 14

Appendix Figure S6 Heatmap of the distance matrix before and after batch effect removal...... 14

Appendix Figure S7 Batch effect removal dramatically reduces the variance of the 2D cell migration data from Lab 1 16

Appendix Figure S8 Batch effect removal dramatically reduces the variance of the 2D cell migration data from Lab 2 18

Appendix Figure S9 Batch effect removal dramatically reduces the variance of the 2D cell migration data from Lab 3 20

Appendix Figure S10 Study design and protocols of the 3D cell invasion experiment................. 22

Appendix Figure S11 3D cell migration distance before and after batch effect removal............ 23

Appendix Table S1 Cumulative variability definitions 24

Appendix Protocol S1 Protocol for HT1080 2-D Migration Live Cell Imaging.............................. 25

Appendix Protocol S2 Protocol for standardized 3D spheroid culture.. 36

Appendix Protocol S3 Protocol for standardized microscopy of 3D spheroid............................. 43

Appendix Protocol S4 Protocol for standardized image analysis of 3D spheroid 45

Appendix Figure S1

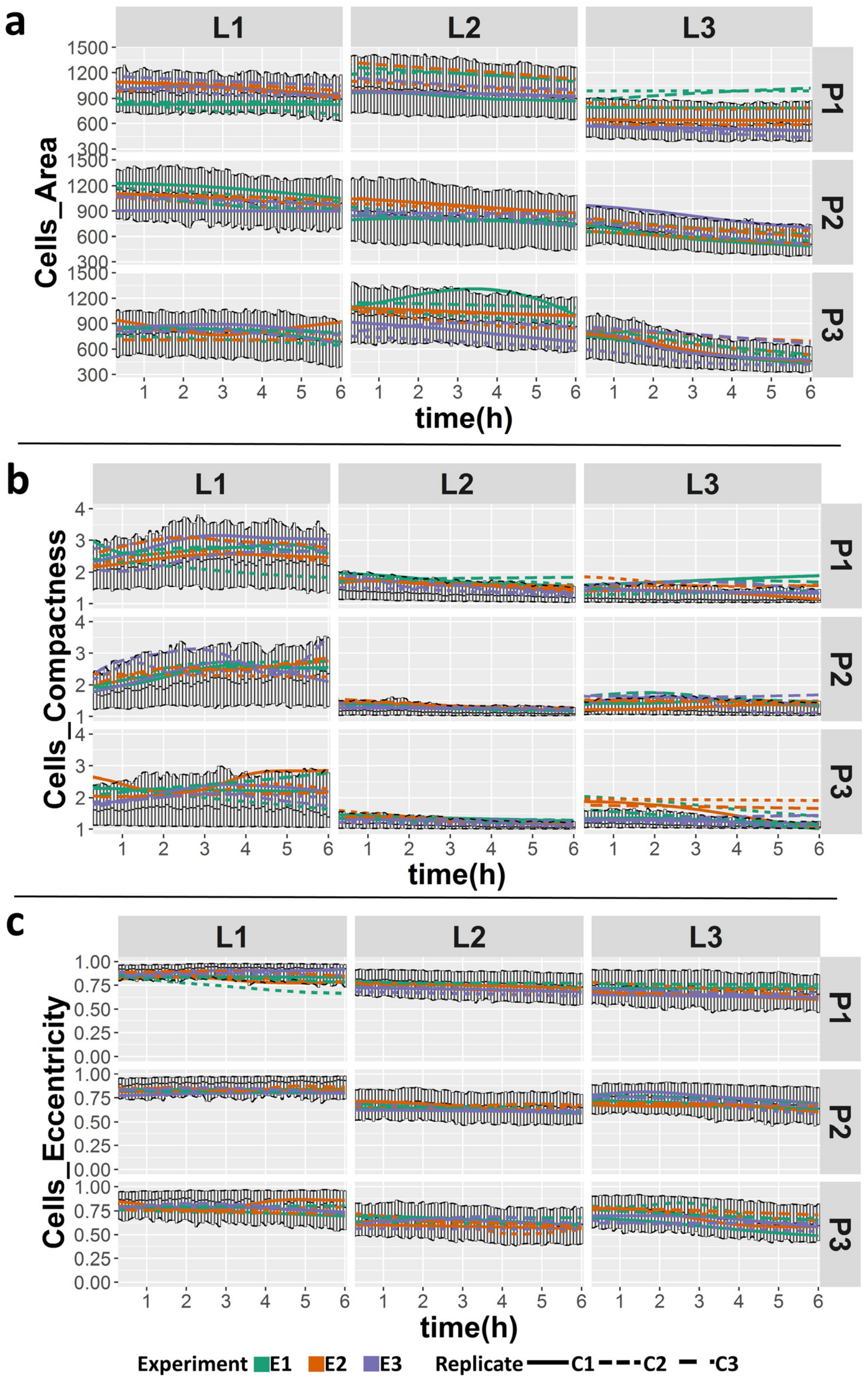

d

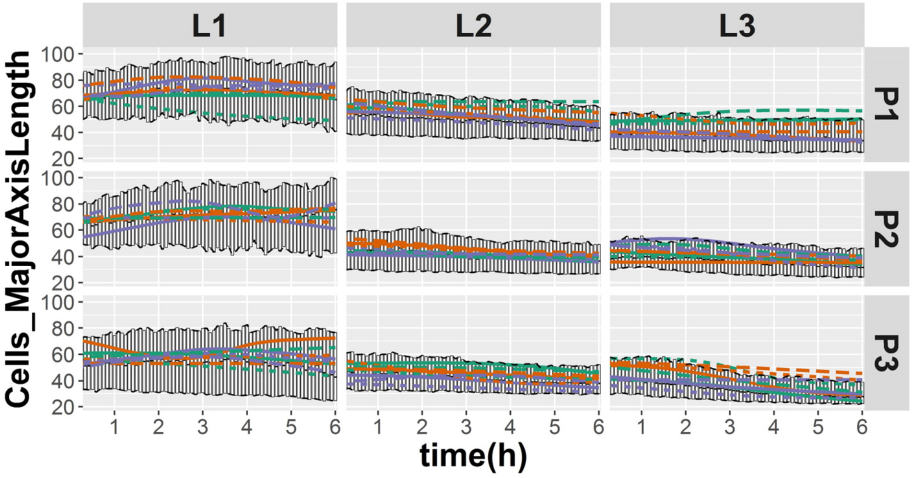

L1
L2
L3
P1
P2
P3
Cells_MajorAxisLength
100
80
60
40
20
1 2 3 4 5 6
time(h)

e

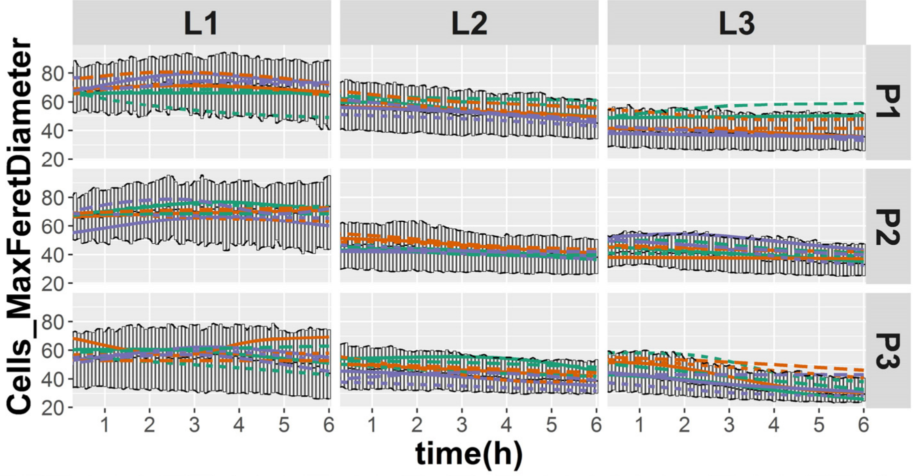

L1
L2
L3
P1
P2
P3
Cells_MaxFeretDiameter
80
60
40
20
1 2 3 4 5 6
time(h)

f

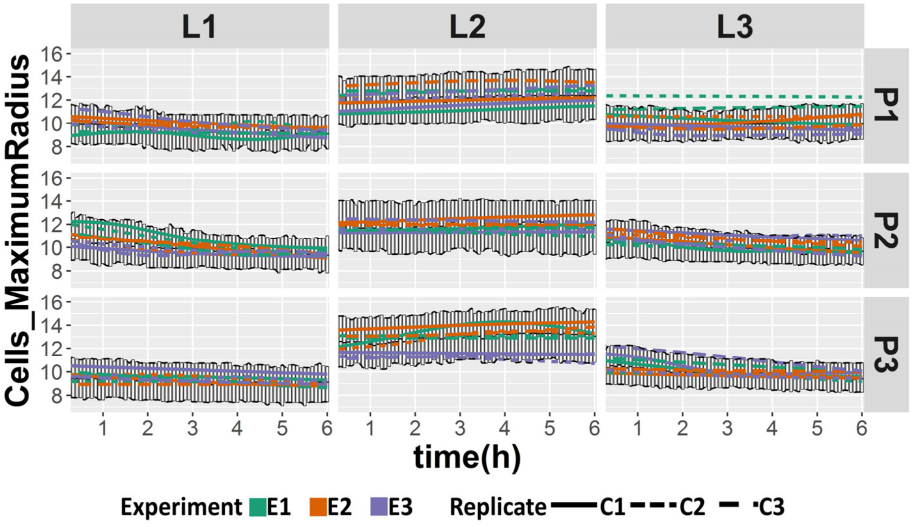

L1
L2
L3
P1
P2
P3
Cells_MaximumRadius
16
14
12
10
8
1 2 3 4 5 6
time(h)
Experiment E1 E2 E3
Replicate C1 C2 C3

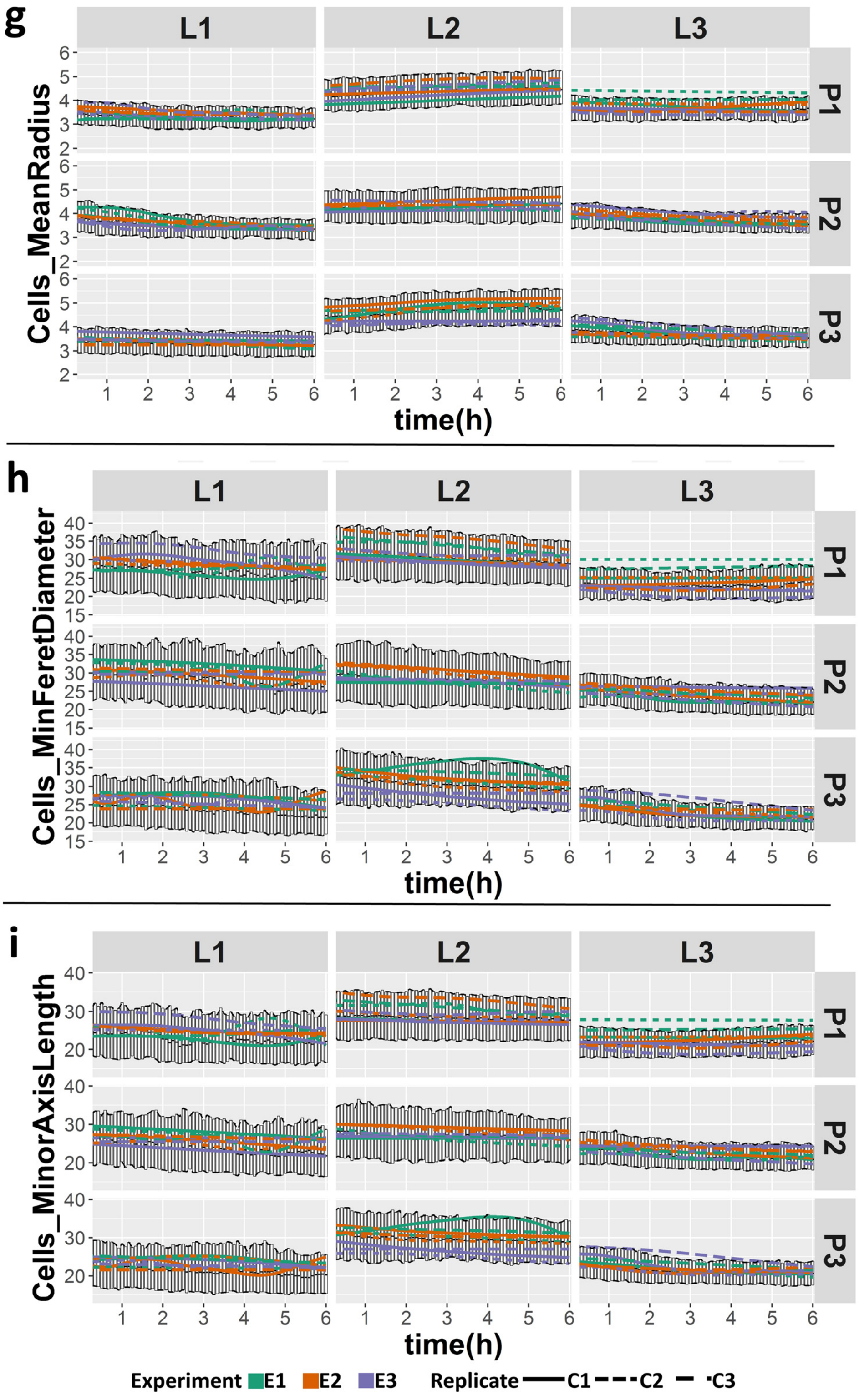
g
h
i
L1
L2
L3
P1
P2
P3
Cells_MeanRadius
Cells_MinFeretDiameter
Cells_MinorAxisLength
time(h)
Experiment E1 E2 E3 Replicate C1 C2 C3

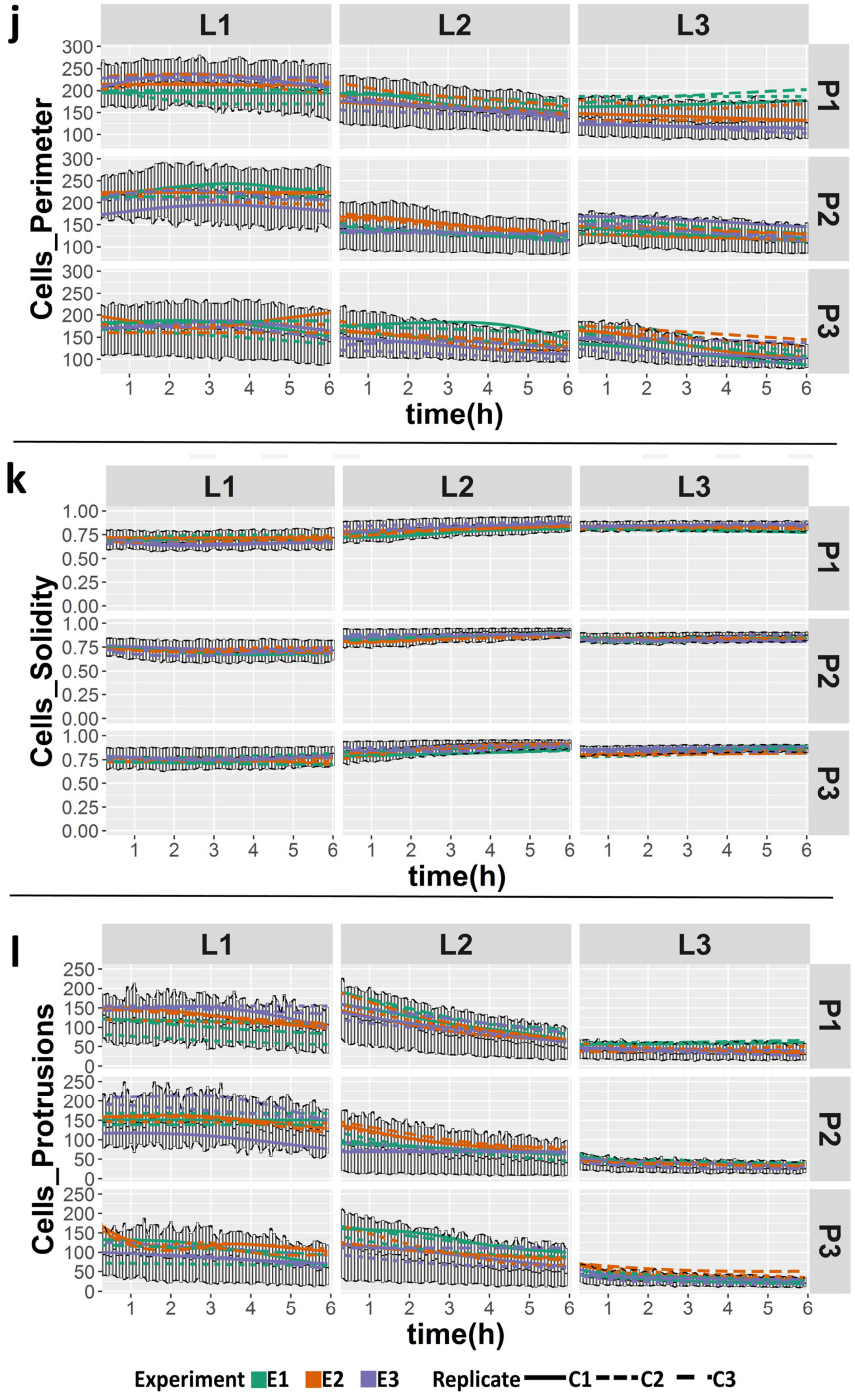

j
L1
L2
L3
P1
P2
P3
Cells_Perimeter
time(h)
k
Cells_Solidity
l
Cells_Protrusions
Experiment E1 E2 E3
Replicate C1 C2 C3

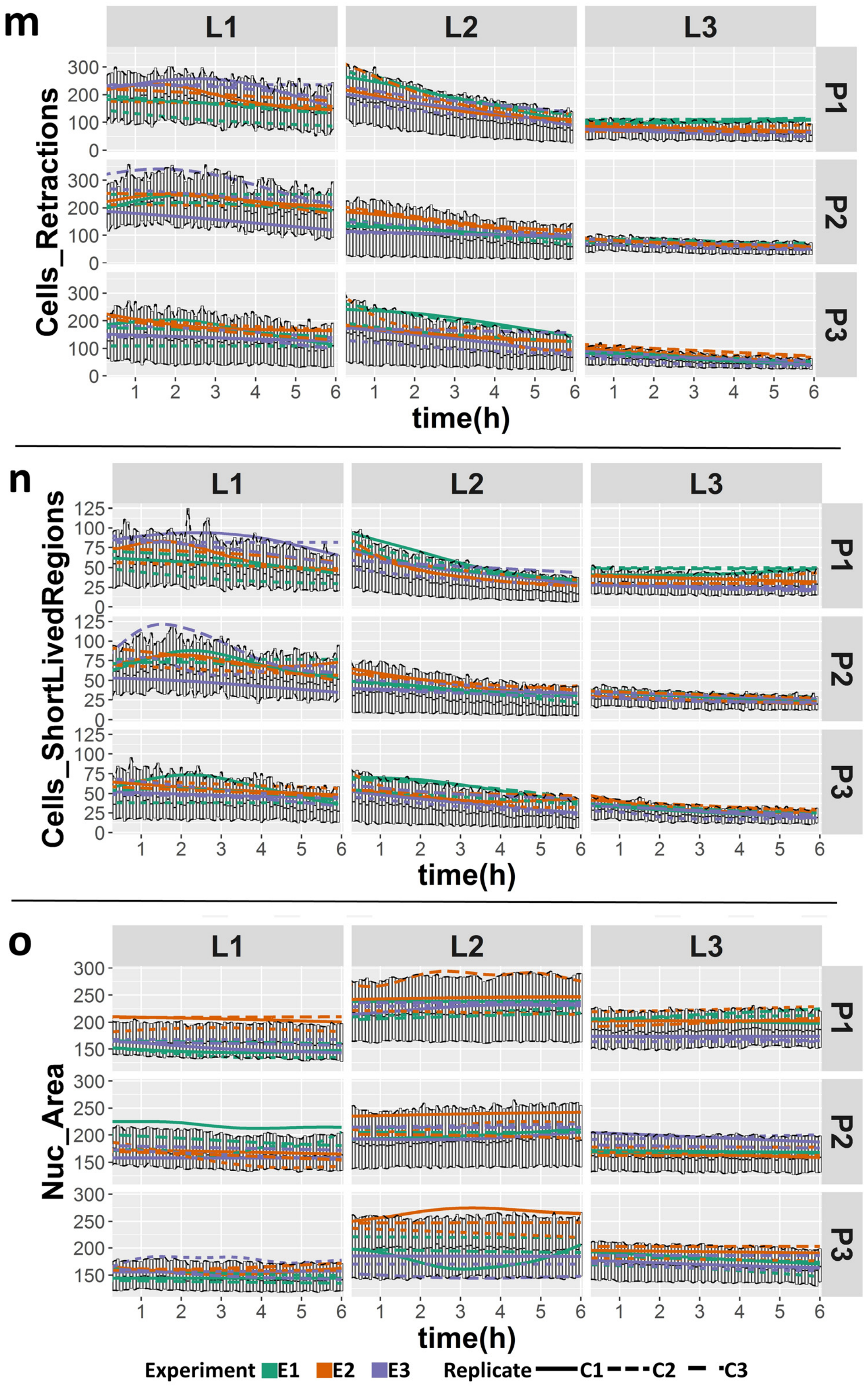
m
n
o
L1
L2
L3
P1
P2
P3
Cells_Retractions
Cells_ShortLivedRegions
Nuc_Area
time(h)
Experiment E1 E2 E3 Replicate C1 C2 C3

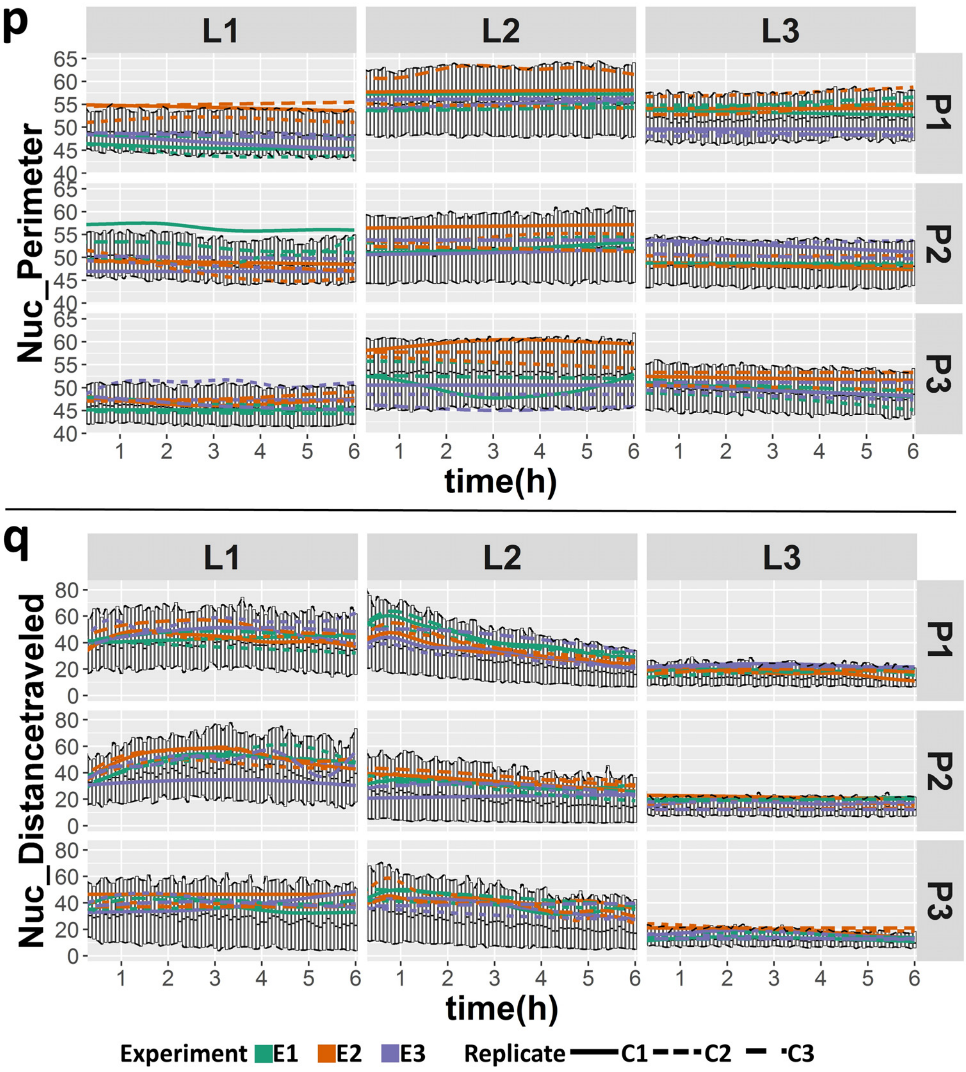

Appendix Figure S1. Quantification of variables over time at different levels of data hierarchy. The graphs display quantifications over time for each laboratory (L1-3), person (P1-3), experiment (E1-3), and technical replicate (C1-3), of the control condition. Results include Cell Area(**a**), Cell Compactness(**b**), Cell Eccentricity(**c**), Cell Major Axis Length(**d**), Cell Maximal Feret Diameter(**e**), Cell Maximum Radius(**f**), Cell Mean Radius(**g**), Cell Minimal Feret Diameter(**h**), Cell Minor Axis Length(**i**), Cell Perimeter(**j**), Cell Solidity(**k**), Cell Protrusions(**l**), Cell Retractions(**m**), Cell Short Lived Regions(**n**), Nuclear Area(**o**), Nuclear Instantaneous Nuclear Speed (INS) (**p**), and Nuclear Perimeter(**q**). Lines in different colours represent three different experiments. Different style of the lines with the same colour represent three different technical replicates from the control condition within one experiment. The error bar indicates the first and third quartiles of the data at each time point.

Appendix Figure S2

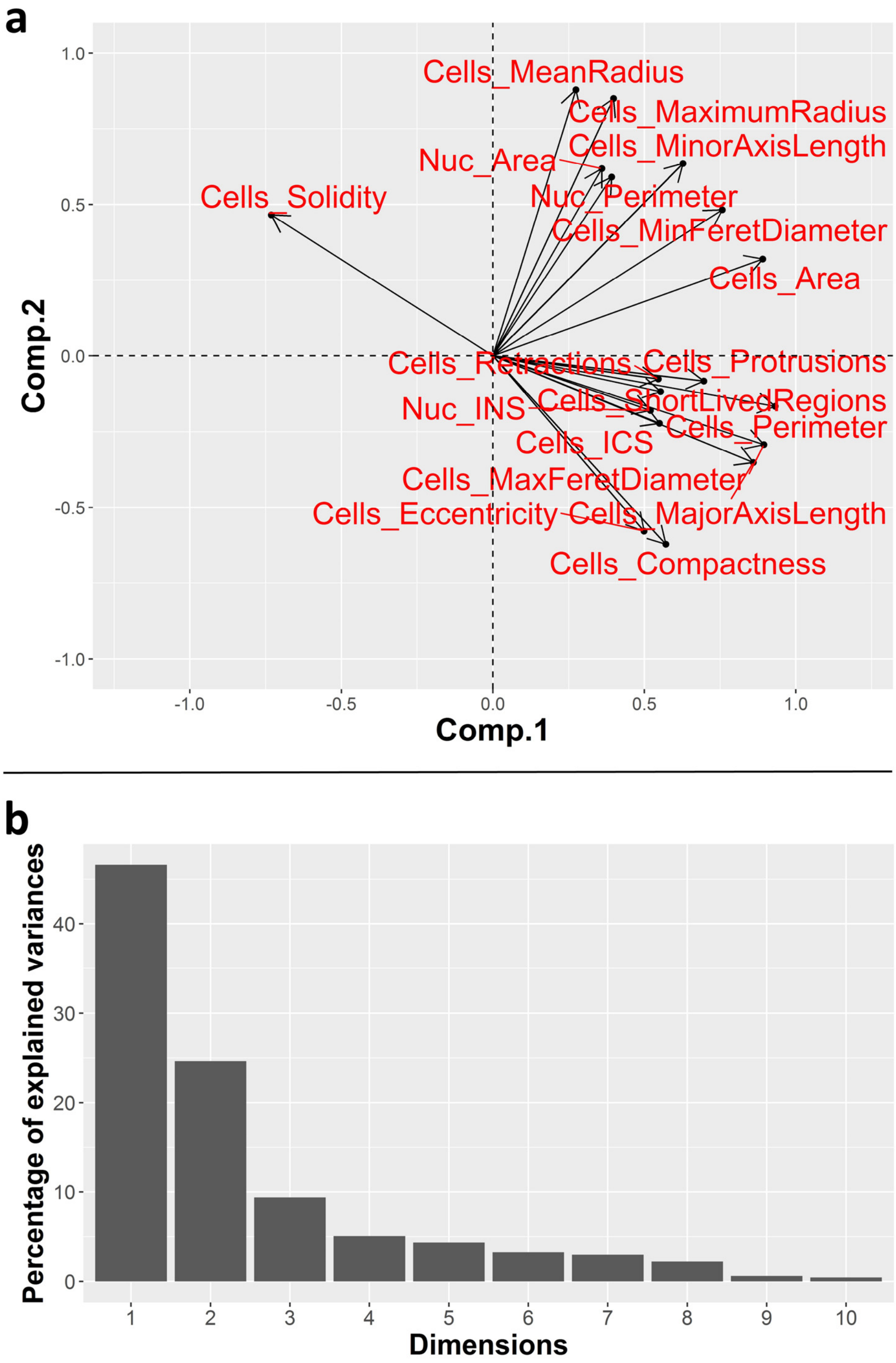

Appendix Figure S2. Overview of the Principal Component Analysis (PCA) results from the 18 variables used in the study. a. Variance map of the PCA analysis. Each arrow shows the relative location of the corresponding variable within the principal component space. **b. Percentage of explained variance by the top 10 principal components**. X axis shows the dimension of the principal component. Y axis shows the percentage of the explained variances by the corresponding principal component.

Appendix Figure S3

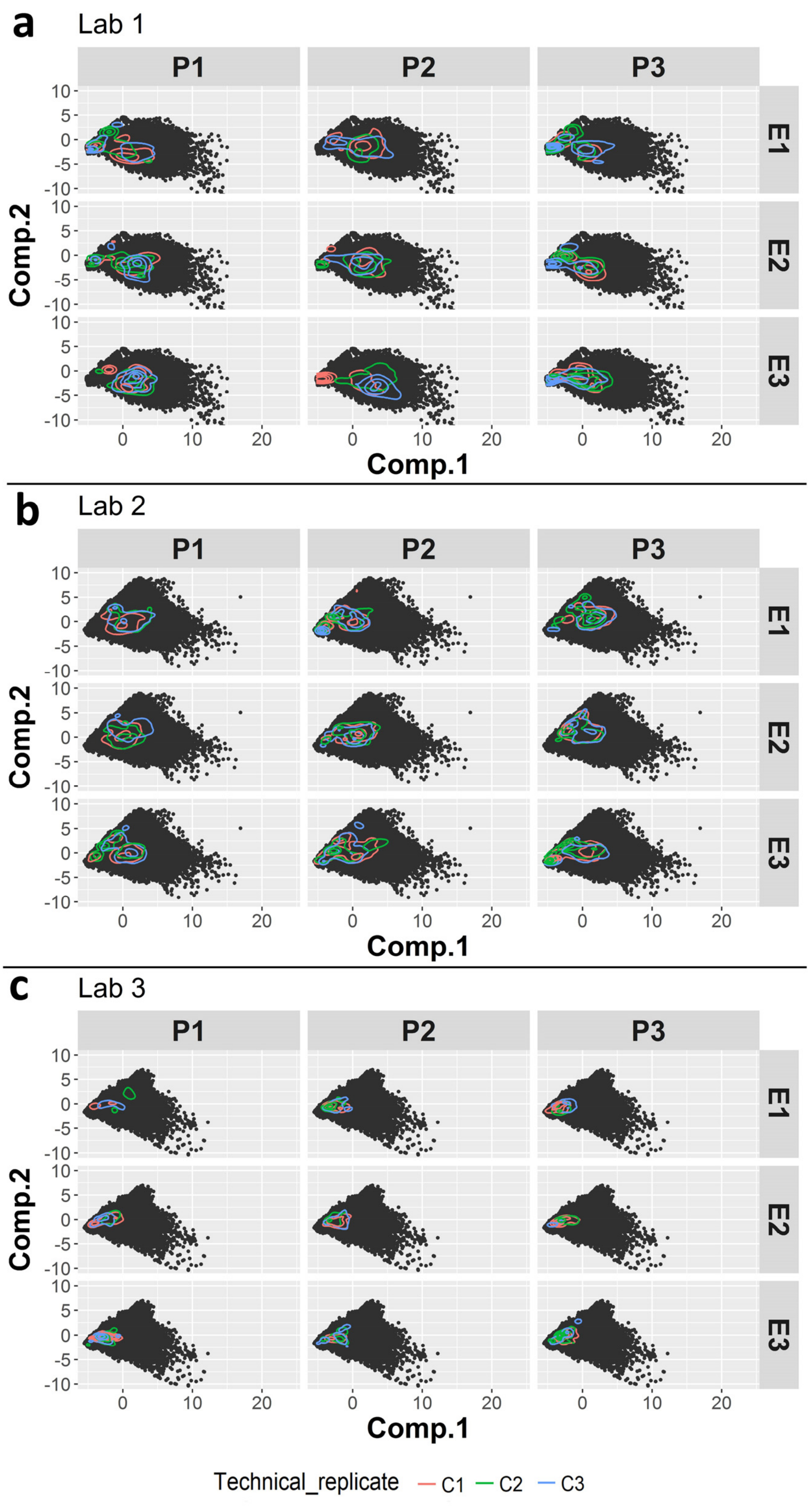

Appendix Figure S3. Principal component analysis results shown for each experiment from each person in three laboratories. Principal component analysis in individual experiments of each person in Laboratory #1(**a**), Laboratory #2(**b**), and Laboratory #3(**c**). Black dots show the position of the first and second principle components for all the observations from each lab as indicated. Each observation is the status of one cell at one time point. Coloured

lines show the 2D density plots of the technical replicates. Each technical replicate within the same experiment is shown in different colours. The principal component space is identical for all the plots.

Appendix Figure S4

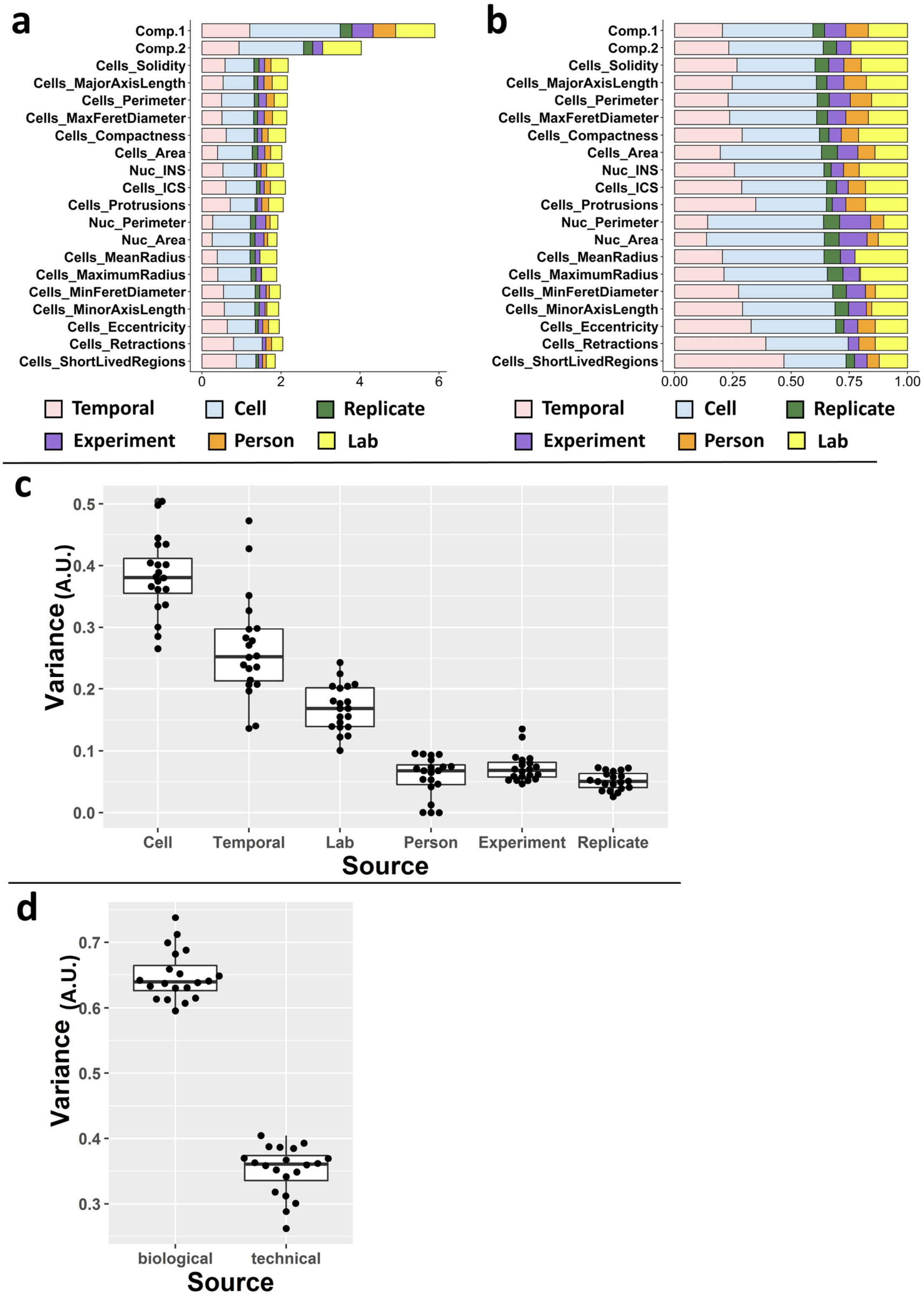

Appendix Figure S4. Variance components of each variable from all data from the control condition hierarchical levels based on the Linear Mixed Effect (LME) model analysis. a-b. Absolute (**a**) or relative (**b**) variance components of each variable from biological and technical sources, including temporal, cell, technical replicate, experiment, person, and laboratory levels based on the LME model analysis. **c**. Boxplot of the absolute variance

components of all the variables from temporal, cell, technical replicate, experiment, person, and laboratory levels based on the LME model analysis. Each dot represents one variable within the corresponding variance level. The data for Lab, person, experiment and technical replicate are identical to Fig. 3c, and are used here for comparison. **d**. Boxplot of the biological (cell and temporal) and technical (technical replicate, experiment, person, and lab) variance components of all the variables based on the LME model analysis. Each dot represents one variable within the corresponding variance category. For the boxplots in **c** and **d**, on each box, the central mark indicates the median, and the bottom and top edges of the box indicate the 1st quartile and 3rd quartile, respectively. The whiskers extend to the most extreme data points not considered outliers. Data between the 1st quartile-1.5* interquartile range and 3rd quartile+ 1.5* interquartile range are considered not outliers.

Appendix Figure S5

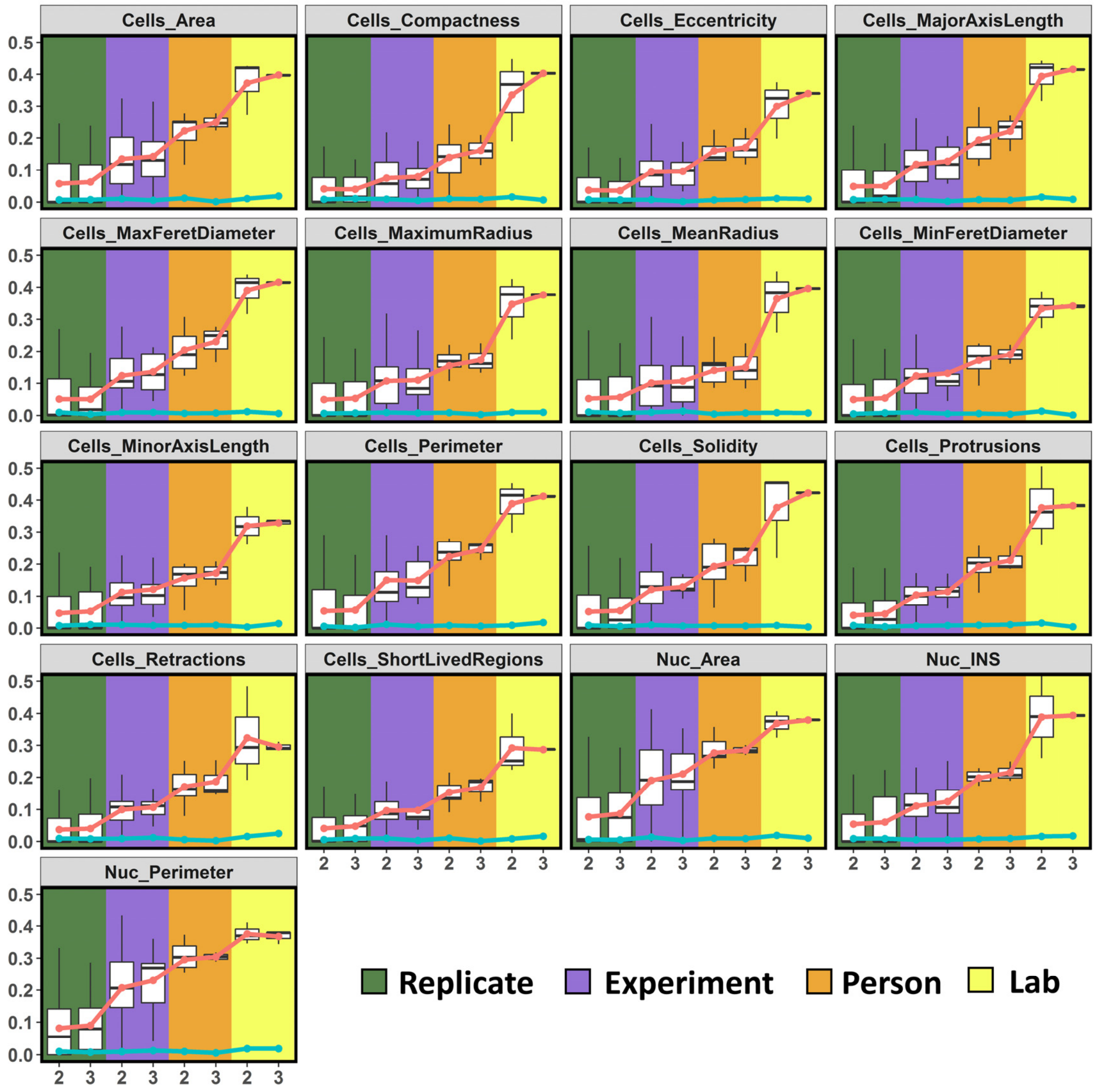

Appendix Figure S5. Cumulative variance of the different hierarchical data levels. Each graph displays the cumulative variance of increasing data hierarchy, including technical replicate (R, green), experiment (E, purple), person (P, orange), and laboratory (L, yellow) levels. Results include Cell Area, Cell Compactness, Cell Eccentricity, Cell Major Axis Length, Cell Maximal Feret Diameter, Cell Maximum Radius, Cell Mean Radius, Cell Minimal Feret Diameter, Cell Minor Axis Length, Cell Perimeter, Cell Solidity, Cell Protrusions, Cell Retractions, Cell Short Lived Regions, Nuclear Area, Instantaneous Nuclear Speed (INS), and Nuclear Perimeter at technical replicate, experiment, person, and laboratory levels. Boxplots show variances with 2 or 3 replicates, experiments, persons, or laboratories, calculated at each level. Red dots show the mean value of the cumulative variance that are linked with red lines. As a control, cyan dots and lines show the cumulative variance of the same data after randomization. For the boxplots in each subfigure, on each box, the central mark indicates the median, and the bottom and top edges of the box indicate the 1st quartile and 3rd quartile respectively. The whiskers extend to the most extreme data points not considered outliers. Data between the 1st quartile-1.5* interquartile range and 3rd quartile+ 1.5* interquartile range are considered not outliers.

Appendix Figure S6

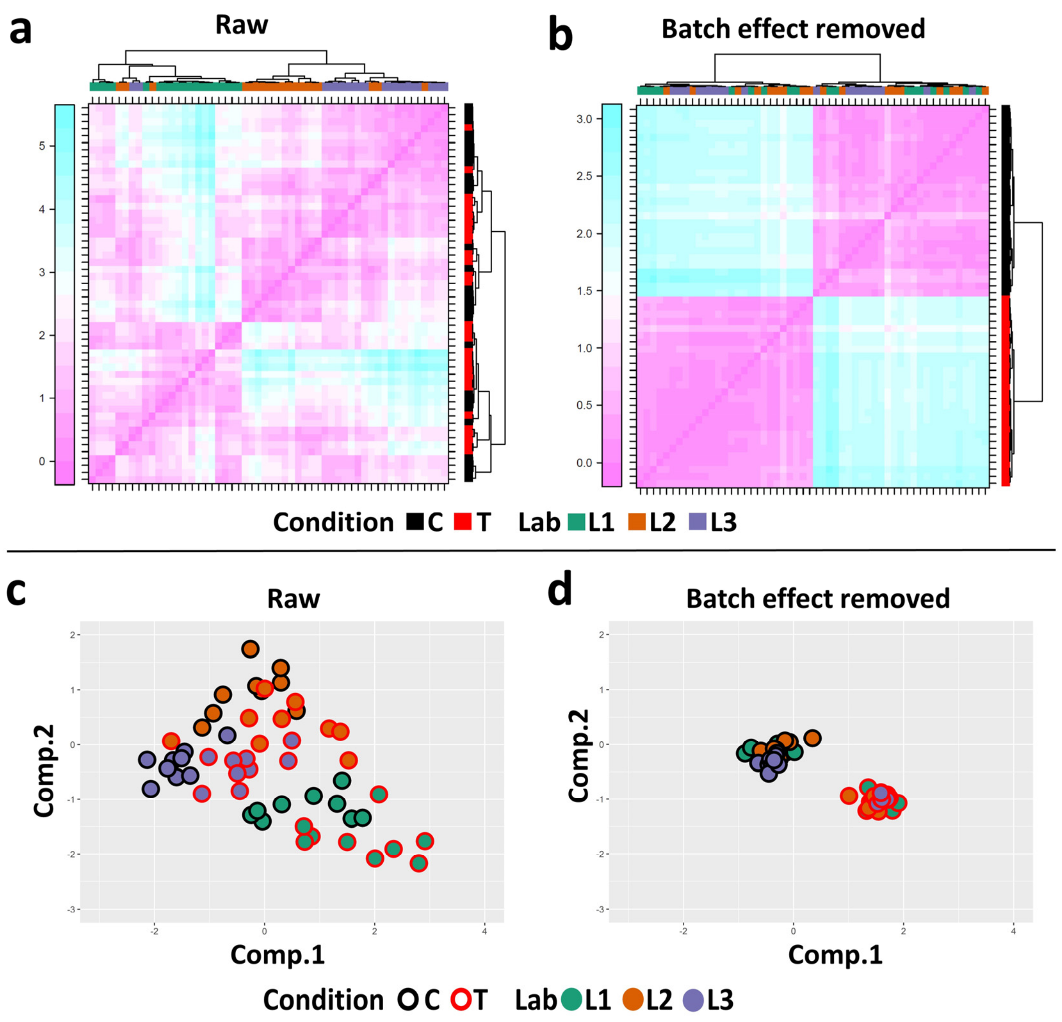

Appendix Figure S6. Heatmap of the distance matrix before and after batch effect removal at the experiment level. a-b Average values of the distance matrix between 1st and 2nd Principal Components per lab, person, experiment, and condition before (**a**) and after (**b**) batch effect removal are shown in the heatmaps. Each row/column corresponds to one experiment. Sorting based on hierarchical clustering. **c-d**. Average values of 1st and 2nd Principal Components at the experiment level before (**c**) and after (**d**) batch effect removal. Each dot represents one experiment. Results from different labs/conditions are coded with different colors as indicated.

Appendix Figure S7

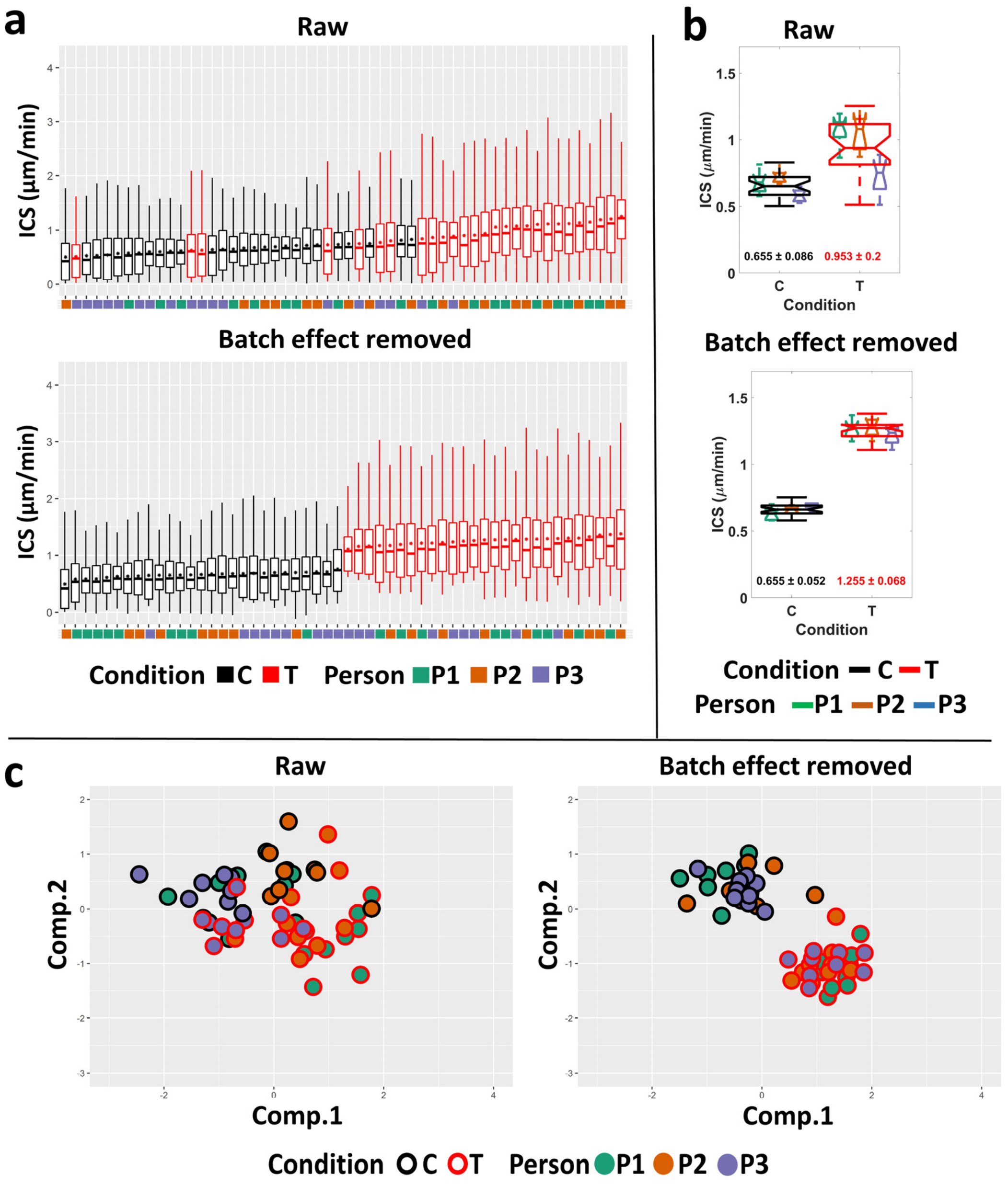

Appendix Figure S7. Batch effect removal dramatically reduces the variance of the 2D cell migration data from Lab 1. a. ICS distribution before (top) and after (bottom) batch effect removal on control (C - black) and perturbed (ROCK inhibition) (T - red). Boxplots display ICS observations for each replicate, sorted by increasing value of the mean. Control and perturbation conditions are shown in black and red respectively. Persons from Lab 1 in which each replicate was performed are color coded below the boxplots. **b. Mean ICS values and variance before (top) and after (bottom) batch effect removal.** Boxplot of mean ICS of each technical replicate from control and perturbed conditions from different persons. Persons are colour coded, while the aggregate results from all the persons in Lab 1 are shown in black (control) and red (perturbed). The numbers below the corresponding boxplot show mean ± standard division of the aggregated control/treated results from all persons in Lab 1. **c. Batch effect removal in principal component data of 2D cell migration data.** Technical replicate of 1st and 2nd Principal Component average values before (left) and after (right) batch effect removal are shown in the same PCA space. Each dot represents one technical replicate.

Results from different persons/conditions in Lab 1 are colour coded as indicated. For the boxplots in **a** and **b**, in each box, the central mark indicates the median, and the bottom and top edges of the box indicate the 1st quartile and 3rd quartile respectively. The whiskers extend to the most extreme data points not considered outliers. Data between the 1st quartile-1.5* interquartile range and 3rd quartile+ 1.5* interquartile range are considered not outliers.

Appendix Figure S8

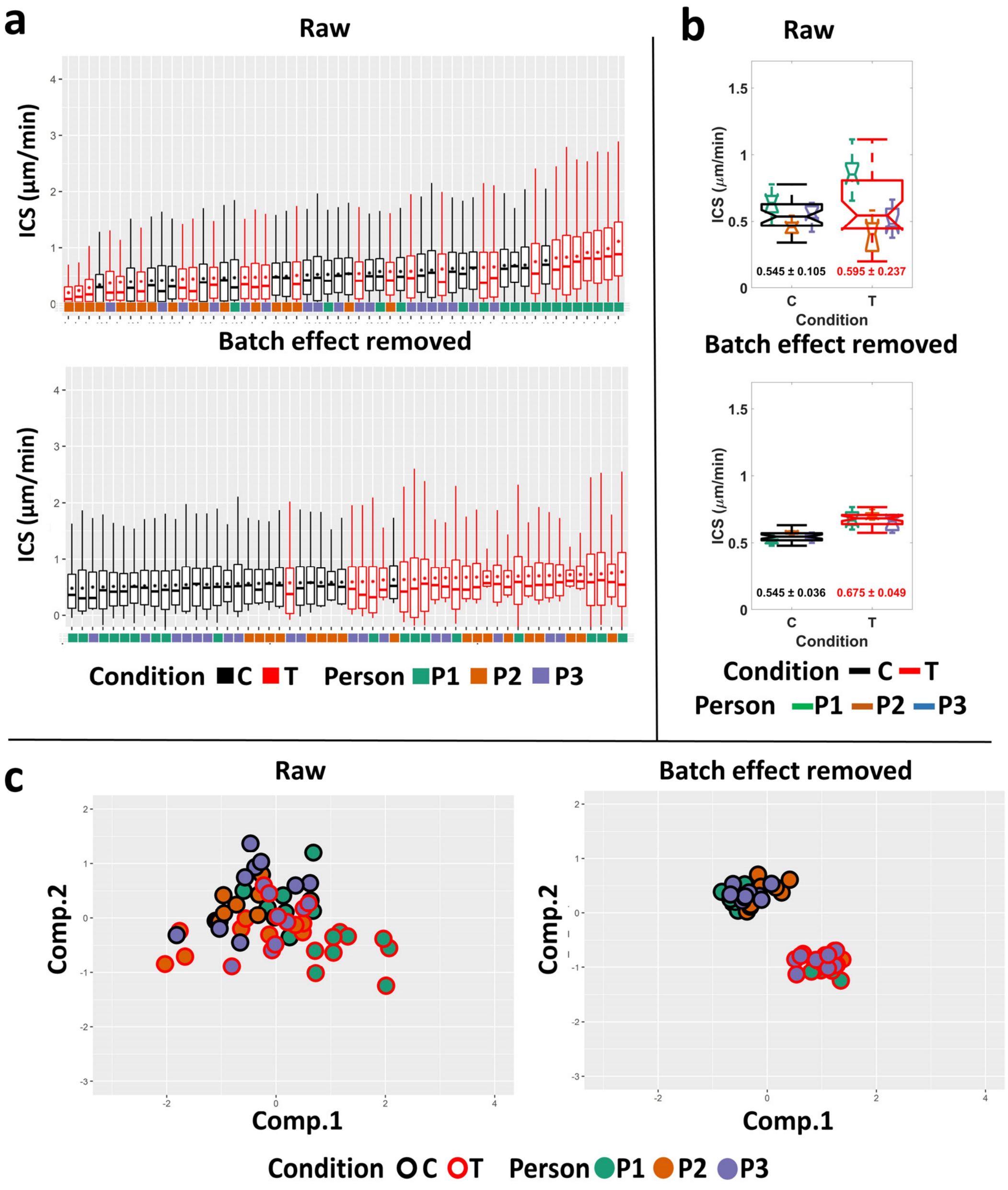

Appendix Figure S8. Batch effect removal dramatically reduces the variance of the 2D cell migration data from Lab 2. a. ICS distribution before (top) and after (bottom) batch effect removal on control (C - black) and perturbed (ROCK inhibition) (T - red). Boxplots display ICS observations for each replicate, sorted by increasing value of the mean. Control and perturbation conditions are shown in black and red respectively. Persons from Lab 2 in which each replicate was performed are color coded below the boxplots. **b. Mean ICS values and variance before (top) and after (bottom) batch effect removal.** Boxplot of mean ICS of each technical replicate from control and perturbed conditions from different persons. Persons are colour coded, while the aggregate results from all the persons in Lab 2 are shown in black (control) and red (perturbed). The numbers below the corresponding boxplot show mean ± standard division of the aggregated control/treated results from all persons in Lab 2. **c. Batch effect removal in principal component data of 2D cell migration data.** Technical replicate of 1st and 2nd Principal Component average values before (left) and after (right) batch

effect removal are shown in the same PCA space. Each dot represents one technical replicate. Results from different persons/conditions in Lab 2 are colour coded as indicated. For the boxplots in **a** and **b**, in each box, the central mark indicates the median, and the bottom and top edges of the box indicate the 1st quartile and 3rd quartile respectively. The whiskers extend to the most extreme data points not considered outliers. Data between the 1st quartile-1.5* interquartile range and 3rd quartile+ 1.5* interquartile range are considered not outliers.

Appendix Figure S9

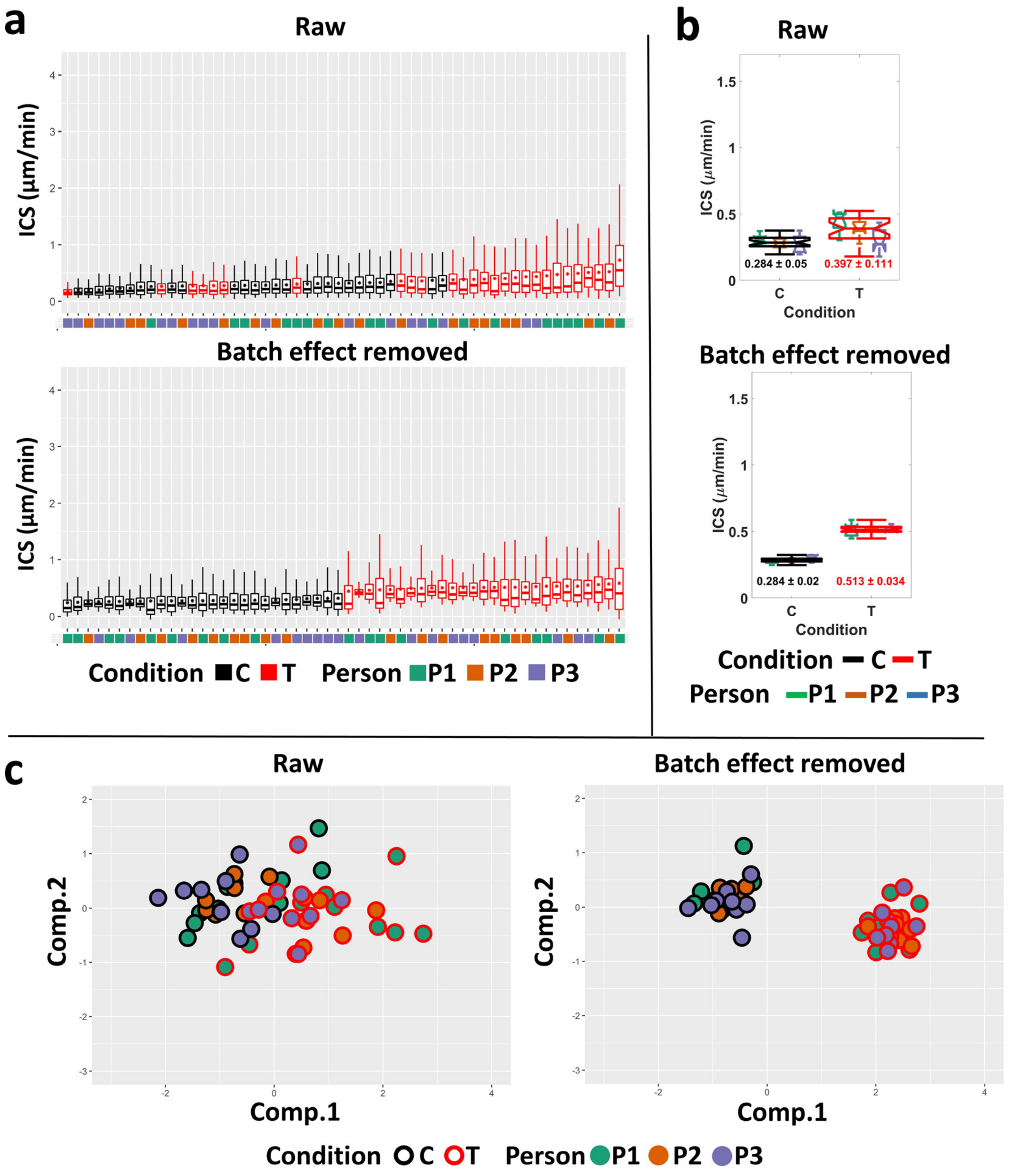

Appendix Figure S9. Batch effect removal dramatically reduces the variance of the 2D cell migration data from Lab 3. a. ICS distribution before (top) and after (bottom) batch effect removal on control (C - black) and perturbed (ROCK inhibition) (T - red). Boxplots display ICS observations for each replicate, sorted by increasing value of the mean. Control and perturbation conditions are shown in black and red respectively. Persons from Lab 3 in which each replicate was performed are color coded below the boxplots. **b. Mean ICS values and variance before (top) and after (bottom) batch effect removal.** Boxplot of mean ICS of each technical replicate from control and perturbed conditions from different persons. Persons are colour coded, while the aggregate results from all the persons in Lab 3 are shown in black (control) and red (perturbed). The numbers below the corresponding boxplot show mean ± standard division of the aggregated control/treated results from all persons in Lab 3. **c. Batch effect removal in principal component data of 2D cell migration data.** Technical replicate of 1st and 2nd Principal Component average values before (left) and after (right) batch

effect removal are shown in the same PCA space. Each dot represents one technical replicate. Results from different persons/conditions in Lab 3 are colour coded as indicated. For the boxplots in **a** and **b**, in each box, the central mark indicates the median, and the bottom and top edges of the box indicate the 1st quartile and 3rd quartile respectively. The whiskers extend to the most extreme data points not considered outliers. Data between the 1st quartile-1.5* interquartile range and 3rd quartile+ 1.5* interquartile range are considered not outliers.

Appendix Figure S10

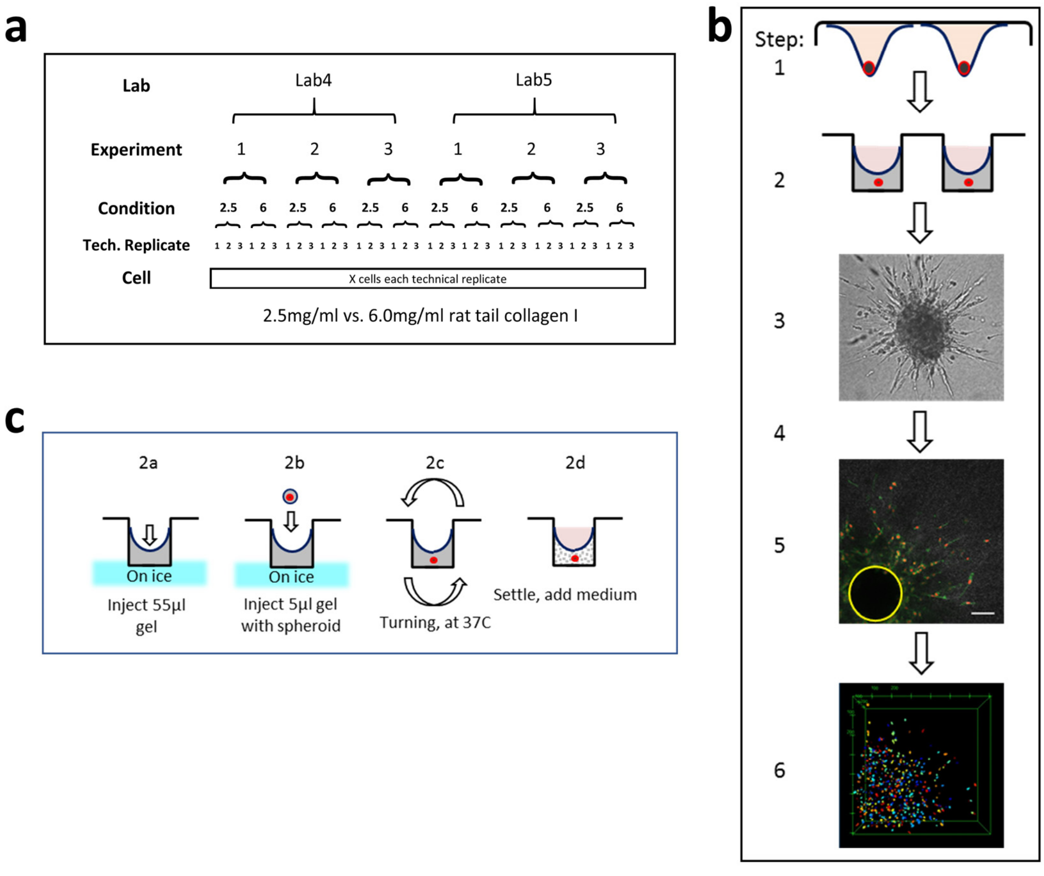

Appendix Figure S10. Study design and protocols of the 3D cell invasion experiment. a. Schematic of the study design, resulting in collagen density dependent 3D invasion**.** The study involved two independent laboratories, three independent experiments in each laboratory, two conditions (2.5 mg/mL or 6 mg/mL collagen) in each experiment, with three technical replicates in each condition. **b. Overview of the pipeline**. Step 1, creation of spheroids (hanging drop assay), 1000 cells/drop, HT1080 cells. Step 2, embedding in rat tail collagen, concentration 2.5 or 6.0 mg/ml, in 96-well imaging plate, 1 spheroid/60µl gel. Step 3, incubation for 24h, 37°C, 10% CO_2. Step 4, 4% PFA fixation and immunofluorescent staining. Step 5, confocal imaging, objective 20x/0.8NA, stack size 708x708x120um, voxel size1.2x1.2x2µm, channels: nuclear DNA (red), F-actin (green) and reflection (grey). Bar: 100µm. Example data represent HT1080 cells, N=3, 2.5 mg/ml. Step 6, 3D nuclear segmentation and quantification of nuclear migration distances from manually annotated spheroid core (yellow circle in step 5). Segmentation precision = 88.05% and recall = 91.00% (see materials and methods). **c. Individual procedures performed in step 2 of the assay (described in b)**.).

Appendix Figure S11

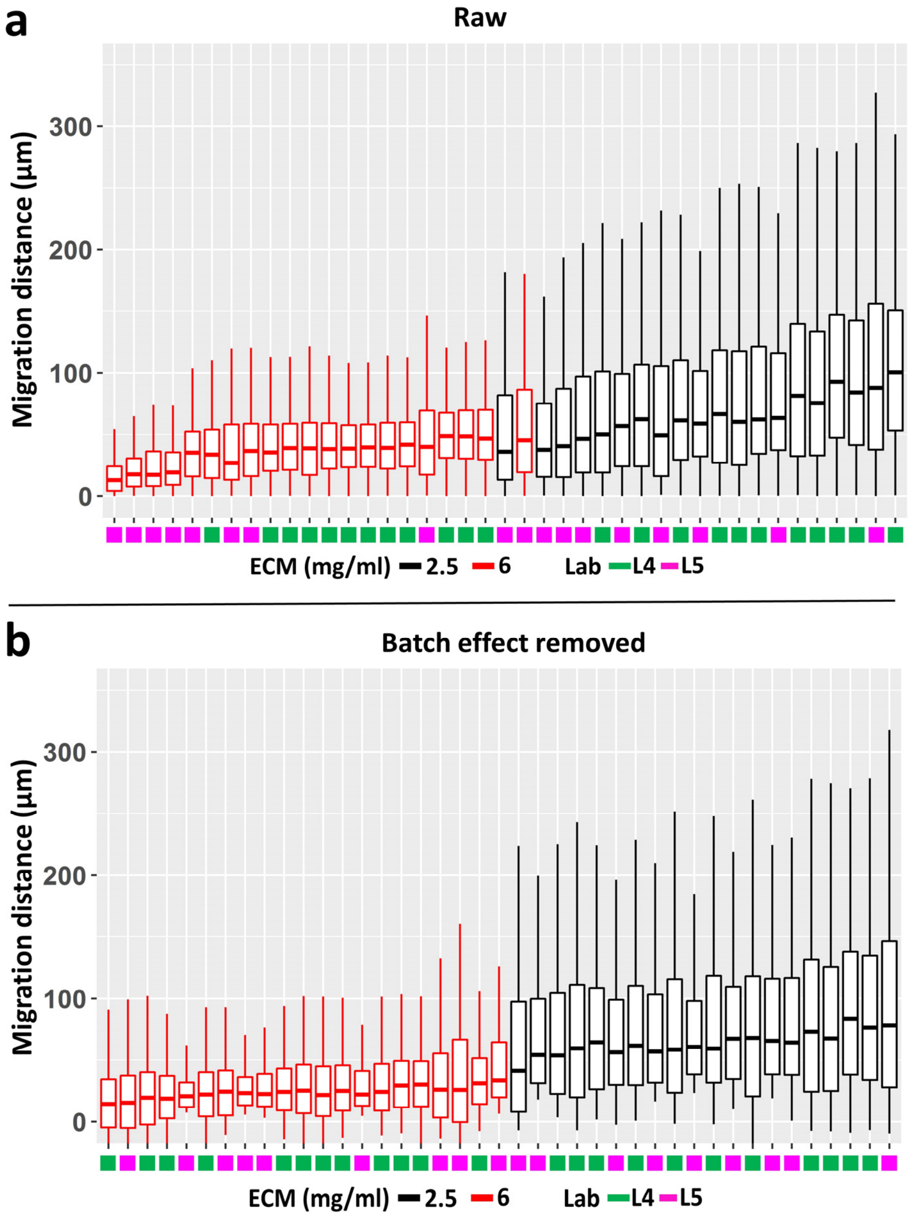

Appendix Figure S11. **3D cell migration distance before (a) and after (b) batch effect removal.** Results are sorted in each case by increasing median order. Each boxplot shows the migration distance of a technical replicate. Laboratory #4 and laboratory #5 are shown with green and orange color respectively below the boxplots. ECM 2.5 mg/mL and 6 mg/mL are shown with black and red color, respectively. For the boxplots, in each box, the central mark indicates the median, and the bottom and top edges of the box indicate the 1st quartile and 3rd quartile respectively. The whiskers extend to the most extreme data points not considered outliers. Data between the 1st quartile-1.5* interquartile range and 3rd quartile+ 1.5* interquartile range are considered not outliers.

Appendix Table S1. Cumulative variability definitions

Description	Formula	Number of possible combinations	Dataset contains all observations that belong to
2 Replicates	$L_i(P_i(E_i(R_{w_2})))$	3*3*3*3 = 81	$L_1P_1E_1R_{1\&2}$<br>(...)<br>$L_3P_3E_1R_{2\&3}$
3 Replicates	$L_i(P_i(E_i(R_{w_3})))$	3*3*3*1 = 27	$L_1P_1E_1R_{1\&2\&3}$<br>(...)<br>$L_3P_3E_3R_{1\&2\&3}$
2 Experiments	$L_i(P_i(E_{w_2}(R_{w_3})))$	3*3*3*1 = 27	$L_1P_1E_{1\&2}R_{1\&2\&3}$<br>(...)<br>$L_1P_1E_{2\&3}R_{1\&2\&3}$
3 Experiments	$L_i(P_i(E_{w_3}(R_{w_3})))$	3*3*1*1 = 9	$L_1P_1E_{1\&2\&3}R_{1\&2\&3}$<br>(...)<br>$L_3P_3E_{1\&2\&3}R_{1\&2\&3}$
2 Persons	$L_i(P_{w_2}(E_{w_3}(R_{w_3})))$	3*3*1*1 = 9	$L_1P_{1\&2}E_{1\&2\&3}R_{1\&2\&3}$<br>(...)<br>$L_3P_{2\&3}E_{1\&2\&3}R_{1\&2\&3}$
3 Persons	$L_i(P_{w_3}(E_{w_3}(R_{w_3})))$	3*1*1*1 = 3	$L_1P_{1\&2\&3}E_{1\&2\&3}R_{1\&2\&3}$<br>$L_2P_{1\&2\&3}E_{1\&2\&3}R_{1\&2\&3}$<br>$L_3P_{1\&2\&3}E_{1\&2\&3}R_{1\&2\&3}$
2 Labs	$L_{w_2}(P_{w_3}(E_{w_3}(R_{w_3})))$	3*1*1*1 = 3	$L_{1\&2}P_{1\&2\&3}E_{1\&2\&3}R_{1\&2\&3}$<br>$L_{1\&3}P_{1\&2\&3}E_{1\&2\&3}R_{1\&2\&3}$<br>$L_{2\&3}P_{1\&2\&3}E_{1\&2\&3}R_{1\&2\&3}$
3 Labs	$L_{w_3}(P_{w_3}(E_{w_3}(R_{w_3})))$	1*1*1*1 = 1	$L_{1\&2\&3}P_{1\&2\&3}E_{1\&2\&3}R_{1\&2\&3}$

Where
$i = 1,2,3$
$\underline{\mathbf{w_n}} = (w_1, w_2, \dots, w_n)$, an n-tuple of natural numbers such that
$\underline{\mathbf{w_n}} \in \binom{\mathbb{N}_3}{n}$, the set of all combination of the elements of $\mathbb{N}_3$ taken in groups of n where $\mathbb{N}_3 \equiv \{1,2,3\}$, the set of the first 3 natural numbers.
For instance, $n = 2 \Rightarrow \binom{\mathbb{N}_3}{2} = \{1\&2, 1\&3, 2\&3\}$ and $n = 3 \Rightarrow \binom{\mathbb{N}_3}{3} = \{1\&2\&3\}$

Protocol for HT1080 2-D Migration Live Cell Imaging

By Jianjiang Hu (jianjiang.hu@ki.se)

Materials:

Cell line: HT1080 labeled with Lifeact-mCherry & H2B-EGFP from Radboud (verified by CRICK). *use the cells for experiment already being passaged at least once after thawing and also before passage 20.

Name	Company	Catalogue Number
high glucose DMEM	gibco	41965-039
FBS	gibco	10270-106
sodium pyruvate	gibco	11360070
penicillin/streptomycin	gibco	15140-122
DMSO	Sigma	D2640
trypsin (10x)	Life technology	15400-054
T-75 flask	SARSTEDT	83.3911.002
T-25 flask	SARSTEDT	83.3910.002
18G syringe needle	KDM	900444
6-well plate	FALCON	353046
96- Imaging Plate CG (Cover Glass)	Mo Bi Tec	5241-20
Collagen I	Corning	354249
Heat denatured 0.5% BSA	Sigma	A2153
Y27632 (ROCK inhibitor)	BD	562822

***Green labelled materials are provided by Staffan lab.**

Cell culture medium: 500 ml high glucose DMEM + 50 ml FBS + 5 ml sodium pyruvate + 5 ml penicillin/streptomycin (10000 U/ml).

FBS free culture medium: 500 ml high glucose DMEM + 5 ml sodium pyruvate + 5 ml penicillin/streptomycin (10000 U/ml).

Trypsin (1×): 5 ml trypsin (10×) + 45 ml PBS

1mM Y27632: Add 2.956 ml ddH_2O into 1 mg Y27632 to get 1 mM solution, aliquot into 100 ul/tube and store in -20℃ (already done).

Heat denatured 0.5 % BSA: Make a 0.5% BSA/PBS solution (add 0.5 g of BSA to 100 ml of 1×PBS) and adjust pH to 7.40. Then heat solution in boiling water for 7-8 min (until it turns opaque). *If solution is over-heated it will turn into a solid gel! Therefore, after 7-8 min of heating, transfer the solution into ice and bring its temperature down to room temperature (already done).

Preparation work:

1. Thaw one tube of the HT1080-Lifeact-mCherry-H2B-EGFP cells in 37℃ water bath.
2. Transfer the cells to a 15 ml tube containing 9 ml of cell culture medium. Add slowly and dropwise.

3. Centrifuge at 240×G for 2 min.
4. Remove the supernatant and re-suspend the cell pellet with 5ml culture medium.
5. Transfer the cells and medium to a T-75 flask and add culture medium to 15 ml.
6. Culture the cells in 37℃ 5% CO_2 incubator.
3. When the cells reach 80-90% confluency, split the cells into three T-75 flasks.
4. Freeze the cells in 3 flasks into 30 cryopreservation tubes (1 ml/tube) for further use.

Freeze cells in T-75 flask:
1. Discard the culture medium and wash the cultured cells with 15 ml PBS once.
2. Add 2 ml trypsin into the flask and put in 37 ℃ incubator for 2-3 min until the cells detached from the surface.
4. Add 15 ml culture medium into the well and gently dissociate the cells with 1ml pipette.
5. Transfer the medium containing cells into a 50 ml centrifuge tube and centrifuge at 240×G for 2 min.
6. Remove the supernatant and re-suspend the cells with 5 ml culture medium.
7. Add 5ml freezing medium (20% DMSO in FBS) slowly into above cell suspension and mix uniformly.
8. Add above cell suspension into 10 cryopreservation tubes (1 ml/tube).
9. Put the tubes into the Mr. Frosty™ Freezing Container (or similar ones) and then in -80℃ fridge.
10. After 24 hs, transfer the tubes into -140℃ fridge for long term storage.

Thaw the cells from stock:
1. Thaw the cells in 37℃ water bath.
2. Transfer the cells to a 15 ml tube containing 9 ml of cell culture medium. Add slowly and dropwise.
3. Centrifuge at 240×G for 2 min.
4. Remove the supernatant and re-suspend the cell pellet with 5ml culture medium.
5. Transfer the cells and medium to a T-25 flask.
6. Culture the cells in 37℃ 5% CO_2 incubator.

Routine cell culture:

Cell passage:
1. Discard the culture medium and wash the cultured cells with 5ml PBS once.
2. Add 1 ml trypsin into the flask and put in 37 ℃ incubator for 2-3 min until the cells detached from the surface.
4. Add 5 ml culture medium into the flask and gently dissociate the cells with 1ml pipette.

5. Transfer the medium containing cells into a 15 ml centrifuge tube and centrifuge at 240×G for 2 min.
6. Remove the supernatant and re-suspend the cells with 1 ml culture medium.
7. Seed suitable amount (e.g. 1:10) of the cells into a new T25 flask containing 5 ml culture medium.
8. Culture the cells in 37℃ 5% CO_2 incubator.

Grow the cells in a T25 flask and passage when the cells reach ~80-90 % confluency. Do not let the cells grow to 100% confluency.

*Avoid everyday passage. e.g. If design the live cell imaging for 3 continuous days, split the cells in advance into 3 flasks with different initial cell number, and then use one of the flasks each day for the cell seeding in step 1.

Live cell imaging:

1. One day before the experiment:
1.0 Cells in T25 flask should reach ~70-80% confluency before experiment.
1.1 Discard the culture medium in T25 flask.
1.2 Add 1 ml trypsin into the flask and put in 37 ℃ incubator for 2-3 min until the cells detach from the surface.
1.3 Add 5 ml culture medium into the flask and gently dissociate the cells with 1ml pipette.
1.4 Transfer the medium containing cells into a 15 ml centrifuge tube and centrifuge at 240×G for 2 min.
1.5 Remove the supernatant and resuspend the cells with 1 ml **culture medium**.
1.6 Count the cell concentration with the Fuchs-Rosenthal Counting Chamber.
1.7 Seed $2x10^5$ HT1080+Lifeact-mCherry+H2B-EGFP cells in one well of the 6 well plate and add 2 ml culture medium.
1.8 Culture the cells in 6-well plate in 37℃ CO_2 incubator overnight and then use as described in 2.2.

2. On the day for live cell imaging:

2.1 Plate coating:
2.1.0 Plate drying:
2.1.0.1 If a new imaging plate is used for the experiment, skip this step.

2.1.0.2 When re-using an imaging plate already used before, take the plate out from the incubator and put it in the hood in the cell culture room for 1 h to ensure that the surface is dry.

2.1.1 Collagen I dilution:
2.1.1.1 Add 10 ul 8.94 mg/ml Collagen I into 884 ul PBS and mix well to obtain homogenous 100 ug/ml Collagen I.
2.1.1.2 Add 200 ul of the 100 ug/ml Collagen I into 800 ul PBS to obtain 20 ug/ml Collagen I.

2.1.2 Plate coating:
2.1.2.1 Add 100 ul of the 20 ug/ml collagen I solution into each of 6 wells of the " Imaging Plate CG (Cover Glass) (96 well plate)" with normal yellow tips.
2.1.2.2 Put the plate in the cell incubator at 37℃ for 2 h.

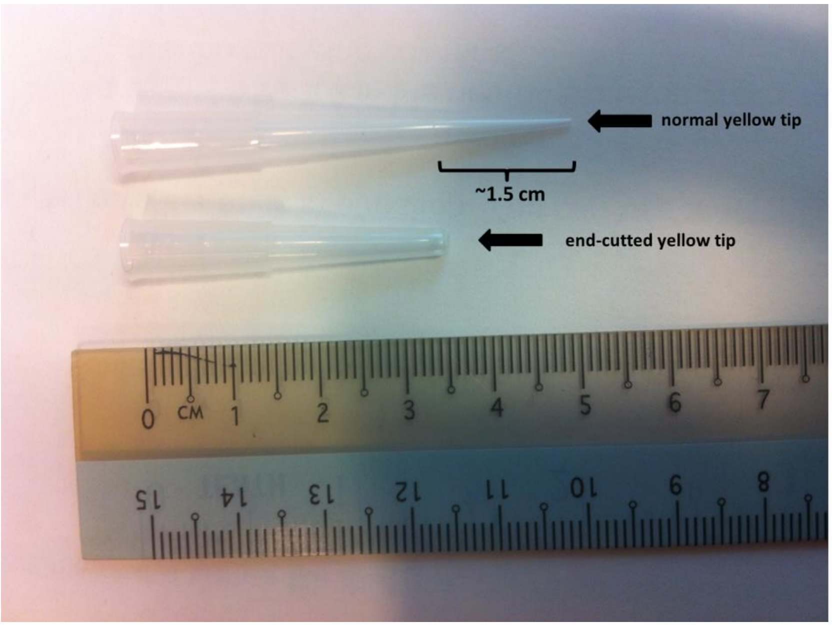

Fig.1 Picture of the normal and end-cutted yellow tip
Cut the sterilized yellow tip with sterilized scissors.
Sterilization of scissors: spray disinfectant (e.g. 70% ethanol or other disinfectant used in cell culture room) on to the scissors and then wipe with clean tissue paper.
*Alternatives: 1) Autoclave the scissors; 2) cut the yellow tips and then autoclave then in advance.

2.1.3 Blocking:
2.1.3.1 Flip the plate upside down and absorb the coating medium with tissue paper gently.
2.1.3.2 Then add 100 ul head denatured 0.5% BSA gently in each well with **end-cutted yellow tips**.

** **Burst the big bubbles remaining in the well with a heated 18G syringe needle carefully, and do not touch the bottom of the wells**

2.1.3.4 Put the plate at 37℃ minimum ~20 min (During which cell preparation (2.2) could be done).

2.1.4 Washing:

2.1.4.1 Flip the plate upside down and absorb the blocking medium with tissue paper gently.

2.1.4.2. Fill each well with 100 ul **FBS free culture medium** with **end-cutted yellow tips**.

2.1.4.3. Flip the plate upside down and absorb the washing medium with tissue paper gently.

2.1.4.4 Fill each well with 100 ul **FBS free culture medium** with **end-cutted yellow tips**.

****After each of 2.1.4 steps, burst the big bubbles remaining in the well with a heated 18G syringe needle carefully, and do not touch the bottom of the wells.**

2.2 Cell preparation:

2.2.1 Discard the culture medium in 6-well plate prepared one day before and wash the cultured cells with 2 ml PBS once.

2.2.2 Add 0.2 ml trypsin into the well and put in 37 ℃ incubator for 2-3 min until the cells detach from the surface.

2.2.3 Add 3 ml culture medium into the well and gently dissociate the cells with 1ml pipette.

2.2.4 Transfer the medium containing cells into a 15 ml centrifuge tube and centrifuge at 240×G for 2 min.

2.2.5 Remove the supernatant and resuspend the cells with 5 ml **FBS free culture medium**.

2.2.6 Centrifuge at 240x G for 2 min.

2.2.7 Remove the supernatant and resuspend the cells in 1 ml **FBS free culture medium**.

2.2.8 Count the cell concentration with the Fuchs-Rosenthal Counting Chamber.

2.2.9 Prepare 1ml cells at the concentration of $5x10^3$ cells/ml in **FBS free culture medium**.

2.3 Cell seeding:

2.3.1 Seed 100 ul cells prepared from 2.2 in each collagen coated well from 2.1.4 (which already contains 100ul FBS free culture medium in it) with **end-cutted yellow tips**.

2.3.2 Tap the culture plate in two vertical directions to make the cell seeding even (For

right handed person, the tap direction should towards the up and right while left handed person to up and left). * Making cell seeding even is important for the following selection of imaging acquisition position.
2.3.3 Put the plate in the cell culture hood for around 10 min to let cells sink and attach to the plate. DO NOT put the plate back into the 37 ℃ incubator immediately after the cell seeding in 2.3.2. This 10 min allows the cells to sink and attach to the plate surface without being disturbed by the medium turbulence caused by the temperature fluctuation.
2.3.4 Put the plate back into 37 ℃ 5% CO_2 incubator and incubate for 2.5 h.

2.4 Drug preparation:
2.4.1 Add 15 ul 1 mM Y27632 into 485 ul **FBS free culture medium** in an 1.5 ml Eppendorf tube.
2.4.2 Put the above medium, together with 500 ul **FBS free culture medium** (in another 1.5 ml Eppendorf tube), into the microscope incubation chamber half an h before imaging acquisition for pre-warming.

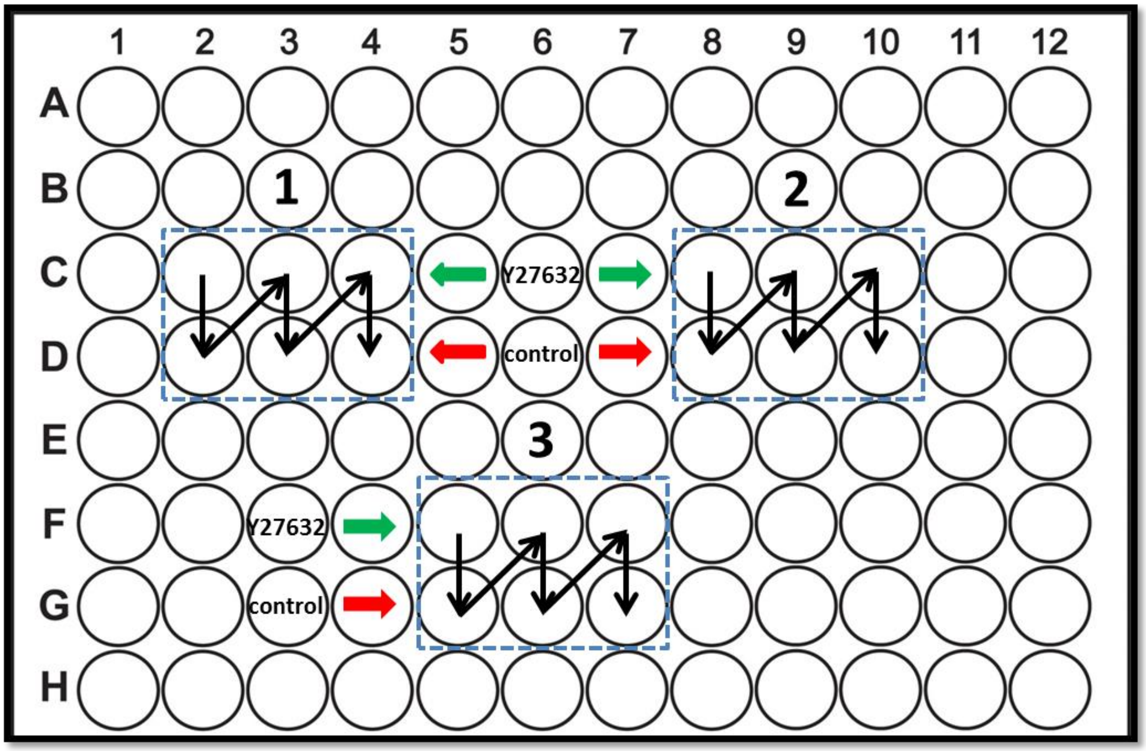

Fig.2 Positions in the plate used for the experiment.
Blue rectangles and the number above them show the 6 wells used for each of the three experiments. **Black arrows** show the acquisition sequence of live cell imaging. **Green**

arrows show three rows for Y27632 groups, while red arrows show three rows for control groups.

2.5 Live Cell Imaging Setting

2.5.1 Put the cell seeded plate from 2.3 on the microscope stage for environment stabilization, during which the microscope setting and position selection could be done (~0.5 h).

2.5.2 Microscope setting:

Principles: 1) With acceptable image quality for CellProfiler auto processing, excite the cells with as low laser power as possible to reduce the phototoxicity. 2) Open the pinhole to maximum. 3) Try to get one large image series in each well. 4) Time interval: 5 min; Total acquisition time: 6 h. 5) Use autofocus system to prevent focus drift during imaging. 6) Find suitable observation position (cell density similar with Fig. 3A) in each well (10× objective might help). Normally start from the center of each well. If too few or too much of cells in center, then look for nearby positions. But do not choose the position too close to the edge of well. 7) During image acquisition, avoid any light from the environment as much as possible (e.g. turn off the computer screen). 8) Also acquire bright field images if possible.

Following are the microscope settings applied in Staffan lab:

Confocal (Orion, Nikon A1) with 488 nm and 561 nm lasers, 20× objective (Plan Apo, NA 0.75), pinhole size: maximum (255.4 µm), 512×512 pixels (0.83 µm/pixel), 5×5 large image (2560×2560 pixels; 2125×2125 µm), zoom 1.5, no averaging

Nikon A1plus

{Scanner Selection}: Galvano
{Detector Selection}: DU4
{Optical Path Mode}: Manual
{First Dichroic Mirror}: 405/488/561/640
{First Filter Cube}:
{Second Filter Cube}: 525/50
{Third Filter Cube}: 595/50

EGFP Channel	{Laser Wavelength}: 488.0	{Laser Power}: 0.3%
	{PMT HV}: 90	{PMT Offset}: -2
mCherry Channel	{Laser Wavelength}: 561.6	{Laser Power}: 1.0%
	{PMT HV}: 100	{PMT Offset}: -2
TD	{PMT HV}: 100	{PMT Offset}: 0

{Pinhole Size (um)}: 255.43
{HV LinearCorrection}: Off
{Scan Direction}: One way
{Scanner Zoom}: 1.499
{Scan Speed}: 1
{Channel Series Mode}: None
{Line Skip}: None
{Frame Skip}: 0
{Line Average Mode}: None
{Line Average/Integrate Count}: 0
Stim1 {Type}: Nothing
{Scan Speed}: 1
Stim2 {Type}: Nothing
{Scan Speed}: 1
Stim3 {Type}: Nothing
{Scan Speed}: 1

(**561 nm laser is specially adjusted on our system, should be ~0.2% on conventional confocal microscope**)
If possible, compare the power of 488 nm and 561 nm lasers with the measurements provided in the attached table with 10x objective and then adjust the laser powers used in the experiment accordingly. **It is necessary to use as low laser power as possible for the live cell imaging. Because the high laser power would be harmful for the cells.**

Large image: find the center of each well and take 5×5 images based on the centers, stitch with no overlap to get the final large image (2560×2560 pixels). If the acquisition software does not have the function of stitch large image, please provide the information of the positions of each small images for further stitching work with other software.
Autofocus: on

Timelapse: time interval: 5 min; total time: 6 h

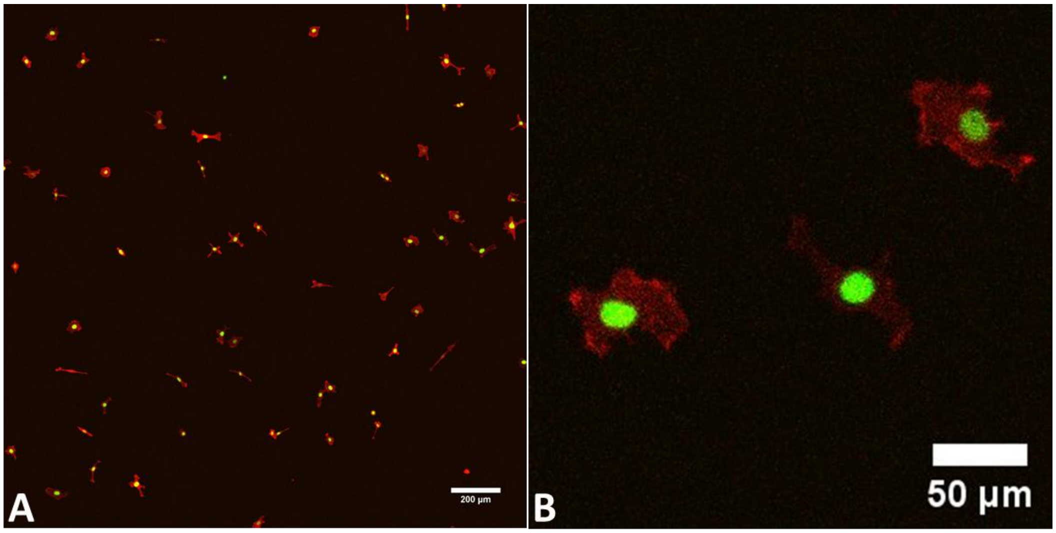

Fig.3 Sample Images

A) Full Image (2125 um ×2125 um, scale bar 200 μm). B) Zoom in.

(Brightness and contrast were adjusted for better display)

Please also see the following KI box link for sample movies:

https://ki.box.com/s/04x31gtkb359t88sjljtw3ss9i6yv1ay

2.6 Drug addition:

2.6.1 Add the drug just before imaging starts.
2.6.2 Add 100 ul medium containing (3 wells)/not containing Y27632 (3 wells) into the wells separately with an end-cutted yellow tips.

2.7 Start the acquisition

2.8 After acquisition

2.8.1 Take the imaging plate out and put it in the cell incubator for further usage.
2.8.2 Save data and turn off the microscope.

2.9 Organization of acquired data

Each person should have one folder, in which there are three folders containing three experiment repeats.

As the data from the timelapse are large, they are not suitable for online transfer. So please copy all the data from same lab into one hard drive with clear annotation and send to Staffan lab by mail. Thanks!

Mail address:

Jianjiang Hu
Clinical Molecular Biology / Lab Staffan Strömblad
Karolinska Institutet
Department of Biosciences and Nutrition
Hälsovägen 7-9
SE-141 83 Huddinge
Sweden

Laser power measurement on Orion with 10x objective, values in uW. Low output 561 10 mW																						
**Date**	**Laser %**	**0**	**5**	**10**	**15**	**20**	**25**	**30**	**35**	**40**	**45**	**50**	**55**	**60**	**65**	**70**	**75**	**80**	**85**	**90**	**95**	**100**
**12-May-2016**	**488 nm**	2,628	88,67	176,6	268,1	356,0	444,3	532,9	621,0	708,8	796,9	883,2	975,0	1062,	1149,	1234,	1319,	1403,	1493,	1587,	1664,	1741,973
**12-May-2016**	**561 nm**	1,338	125,3	251,2	372,9	500,4	626,4	745,7	874,7	999,3	1123,	1248,	1369,	1498,	1623,	1749,	1868,	1990,	2112,	2240,	2370,	2490,843

Appendix I. Protocols for standardized 3D spheroid culture

Preparation of Methylcellulose 12 mg/ml (5x):
Weigh 6 g of methyl cellulose (Sigma, Cat.: M6385) in a 500 ml glass bottle, add a magnetic stir bar and autoclave the bottle. Add 250 ml of DMEM (Dulbecco Modified Eagle Medium, High Glucose, Gibco/Thermo Fischer Scientific, 10938-025) to the autoclaved methyl cellulose and incubate for 15 min at 60°C (shake the bottle to destroy clumps of methyl cellulose). Stir on a magnetic stirrer for 20 min, then add another 240 ml of DMEM, and continue stirring for 4 hours at room temperature. Keep the bottle overnight at 4°C. Add 10 ml of Penicillin-Streptomycin stock solution (10,000 U/mL, Gibco/Thermo Fischer Scientific, 15140122) and stir thoroughly. Centrifuge for 20 min. at 4000 rpm, at 4°C and carry the supernatant into 50 ml Falcon tubes.

Cell culture:
Human wild-type HT1080 fibrosarcoma (ACC315; DSMZ Braunschweig) are cultured (37°C at 10% CO_2 humidified atmosphere) in medium (DMEM, High glucose, Gibco) supplemented with 10% of fetal calf serum (FCS; Sigma Aldrich, F7524), Penicillin-Streptomycin (100 U/mL, Gibco/Thermo Fischer Scientific, 15140122), L-glutamine (2 mM) and sodium pyruvate (1mM; both Invitrogen) in a T75 culture flask. Grow cells up to 85-90% confluency before using.

Generation of 3D spheroids (hanging-drop culture [1]):

This protocol describes the generation of multicellular spheroids of HT-1080 cells. These cells require supplemental collagen for proper aggregation.

- Check cells in T75 flask for correct confluency (85-90%);
- Remove medium (as prepared for cell culture) and wash adherent cells with 1x PBS (room temperature);
- Detach cells with 2.5 ml of 2 mM EDTA (Invitrogen/Thermo Fischer Scientific, 15575020) in PBS for 10 minutes in a 37°C incubator;
- Check if the cells detached, mix the cell suspension with 7,5 ml medium and transfer it to a 15 ml tube;
- Spin down for 5 min at 1000 rpm;
- Remove the supernatant;
- Resuspend the pellet in 1 ml of medium and count the cells;
- Create a $2*10^6$ cells/ml suspension by addition of medium;
- Add 100 µl of cell suspension to a 15 ml tube;
- Add 1 ml methylcellulose 5x (end concentration 4 mg/ml);
- Add 2.6 µl of rattail collagen type I from stock (9.59 mg/ml, Corning, 354249, lot 6102001) to obtain a final concentration of 5 µg/ml of collagen in the suspension;
- Add 3.9 ml medium to have 5 ml of cell suspension with 40.000 cells/ml;
- Create 25 µl drops with a 200 µl multichannel pipet in the (hydrophobic) lid of a 15 cm culture dish (Greiner Bio-one, 639160);
- Put the plastic dish upside-down on the lid with the drops and gently turn the dish.
- Add 5 ml PBS 1x to the dish to avoid dehydration of the droplets;

- Incubate overnight at 37°C with 10% CO_2;

Spheroid embedding in 3D collagen:

This protocol describes the embedding of HT1080 multicellular spheroids in rattail collagen I, in 96 well plates, using 1000 cells per spheroid, 1 spheroid per gel, in rattail collagen of 2.5 or 6 mg/ml. The protocol has been based on the protocol of Wolf et al., 2013 and Haeger et al. 2014, which describe spheroid embedding in low number of drop gels for different collagen concentrations [2,3].

The protocol aims at embedding approximately 18 spheroids per rattail collagen concentration. The successfully embedded spheroids will be divided into 3 groups with at least 3 technical replicates per group, containing a medium control, a vehicle control such as DMSO and a molecular interference such as a FAK inhibitor.

The embedding process consists of two steps: first, gel droplets without spheroids are deposed into the middle of the wells of a 96-well plate. Second, before polymerization of the gel, a small droplet of gel containing a spheroid is precisely pipetted into the middle of the gel in each well. The advantage of this protocol is that the position of the spheroids can be better controlled compared to direct deposition of a complete gel with a spheroid. Furthermore, all spheroids will sink down to the bottom of the gel droplet and therefore their position with respect to each other is synchronized in time. As a result, spheroid height with respect to the bottom of the plate can be tuned by applying a proper turning sequence during the polymerization process.

Use only one set of pipettes which have been properly calibrated! Deviations in the volumes can strongly influence the consistency and polymerization time of the gel, resulting in altered spheroid invasion phenotypes and bad control over spheroid positioning. Start this protocol with the 2.5 mg/ml concentration and continue with 6 mg/ml in the same plate, after polymerization of the first concentration.

- Aspirate PBS form the hanging drops dish;
- Check the spheroids in the hanging drops by putting the dish with lid side down on a microscope. Mark the good (round, compact) spheroids;
- Cool the 96-well imaging plate *(µ-Plate 96 Well, cell culture surface coating, sterile, Thermo scientific NUNC #165305)* and 5x 1.5 ml pointy Eppendorf tubes on ice;
- Harvest the spheroids: take out the wrong spheroids with P1000 pipet with blue tip and rinse 32 good spheroids from the inclined lid with 5 ml PBS. Transfer the PBS with the spheroids to a 15 ml tube;
- Let the spheroids sink to the bottom of tube (about 3-5 minutes). Aspirate carefully and take care that there is 20 µl left at the pellet of the spheroids;
- Add 3 ml PBS and gently invert the tube 5x times (a wash step);
- Let spheroids sink to the bottom again and aspirate carefully;
- Add 3 ml medium and let the spheroids collect at the base of the tube;
- Start preparing the gels:

Prior to addition of the collagen solution, a reagent mix of 10x PBS, 1N NaOH and MilliQ is prepared and divided in a part to be used for preparation of the first gel (step 1) and a part for the preparation of the second gel containing the spheroids (step 2). To improve control over the consistency of the gel, collagen solution is added to the reagent mix of each step just before deposition of the gel in the wells. The amount of NaOH added is carefully determined beforehand so that the final prepared collagen gels have the correct pH. Monitoring pH and keeping to the temperature in the protocol below is essential to obtain collagen gels with reproducible density (pore size).

- Prepare a reagent mix for one collagen gel concentration in one cooled Eppendorf tube; Use the calculated reagent mix volumes for current RT collagen stock:
  Current collagen stock: Corning Collagen Type I, ref 354249, lot 6102001, 9.59 mg/ml

	2.5 mg/ml mix 5x 385 µl (for 6 gels)	6.0 mg/ml mix 5x 385 µl (for 6 gels)
PBS 10x	154 µl	154 µl
NaOH 1N	10 µl	24.1 µl
Milli Q	874.15 µl	157.55 µl

Start with 2.5 mg/ml and then use calculations for 6mg/ml later on in the protocol

- Distribute 1/5 of the mix over each of the 4 remaining Eppendorf tubes on ice (207,63 µl for 2.5 mg/ml and 67.13 µl for 6.0 mg/ml). Discard the tube with the remaining mix;
- Prepare the first gel without the spheroids: take up collagen very slowly (100.36 µl per ep for 2.5 mg/ml and 240.88 µl for 6.0 mg/ml) and add it to 3 of the 4 Eppendorf tubes on ice. Mix it thoroughly (10x up and down). *Use a P1000 pipette;*
- Add 77 µl of medium <u>without spheroids</u> to the same 3 Eppendorf tubes with mix and collagen solution. *The liquid collagen gel should appear transparent to very light yellow. Use a P200 which has been cut to increase the opening diameter;*
- Add the 3 mixes together in one of the 3 Eppendorf tubes and throw the remaining 2 tubes away. *Keep the gel on ice!*
- Aliquot the first gel into 18 wells: while the plate is on ice, pipet 55 µl gel in the middle of the wells. Avoid air bubbles;
- Check the pH of the remaining gel with pH paper (Merck Millipore pH 5.5-9.0 Neutralit). 2.5 mg/ml should be between 7.0 and 7.5 and 6.0 mg/ml should be near 7.0. *Note: a wrong pH strongly influences polymerization time and the consistency of the gel and therefore the position of the spheroids in the gel after polymerization!*
- Prepare the second gel containing the spheroids: add rattail collagen to the last Eppendorf tube on ice (100.36 µl for 2.5 mg/ml and 240.88 µl for 6.0 mg/ml). *Use a P1000 pipette with a blue tip*;
- Add 77 µl medium <u>with spheroids</u> to tube and mix gently and thoroughly (pipet 10x up and down). *Use a P200 with a cut tip to increase the opening diameter;*
- Take out 5 µl gel containing a single spheroid and deposit the volume in the middle of the gel, close to the bottom of the well. The total gel volume will be 60 µl per well. *Keep the tube containing the spheroids on ice while adding spheroids to 96 well plate!*
- Complete the 18 wells for 2.5 mg/ml;

- Polymerize the gels in such a manner that the spheroids remain in three dimensions, not touching any surfaces, while remaining within the working distance of the imaging objective to be used:

  2.5mg/ml:

  - Wipe the base of the plate (to remove ice);
  - Start the timer. Place the plate for 5 min 15 s in the incubator the correct way up (to allow all spheroids to sink to the bottom). *Harvest the spheroids for the 6.0mg/ml condition;*
  - While in the incubator, invert the plate every 60 seconds until polymerized (The gel will appear milky, 14-15 minutes on the timer);
  - After polymerization, leave the plate in normal position in the incubator until 18 minutes have passed on the timer; *Prepare the reagent mix without collagen solution for the 6 mg/ml gel;*
  - Place the plate back on ice during further preparation of the 6mg/ml gel;

  6.0 mg/ml:

  - Start the timer. Keep plate 4 minutes on ice (all spheroids sink to bottom);
  - Wipe the base of the plate (to remove ice);
  - Place the plate upside-down in the incubator for 2.5 minutes;
  - While in the incubator, invert the plate every 60 seconds until polymerized (~9 minutes on the timer);
  - After polymerization, leave the plate in normal position in the incubator until 30 minutes have passed on the timer. *Prepare the media for the different conditions in the plate. Keep the media at 37*°C;

- After polymerization of both collagen concentrations, check embedded spheroids and divide the good spheroids into 3 groups, randomly distributed over the plate: for example, Medium, DMSO and a pharmacological inhibitor;
- Make sure every group contains at least 3 successful technical replicates (briefly inspect spheroids by microscope);
- Add 175 µl medium to each well: add medium with pharmacological inhibitor (e.g. FAK inhibitor, TOCRIS PF573228, 2 µM work concentration in medium), medium with a vehicle control (e.g. in case of FAK inhibitor, DMSO) and medium only to the wells;
- Take time = 0 snapshots of each spheroid with a brightfield microscope;
- Incubate 24 hours at 37°C to establish cancer cell invasion in three dimensions, prior to fixation.

Calculation of reagent quantities for preparation of a rattail collagen gel:

- *Variables, chosen by experimenter:* V_{final} (final volume, µl), C_{stock} (stock concentration of the collagen solution, mg/ml), C_{final} (final collagen concentration of the gel, mg/ml) and *p* (calibration factor to establish the correct pH of the collagen gel).
- *Reagent quantities to be calculated:* mQ (milliQ water, µl), Cells (medium with cells, µl), NaOH (solution of potassium hydroxide, 1N, µl), Coll (Collagen stock solution, µl) and PBS10x (10x PBS solution, µl).
- *Formulas:*
  $Coll = C_{final} * V_{final} / C_{stock}$

Cells = 0.2 * V_{final}
NaOH = *p* * Coll
PBS10x = (V_{final} - Cells) / 10
mQ = V_{final} – Cells – PBS10x – Coll – NaOH

The factor *p* was determined upon arrival of a new batch of rattail collagen solution, which has its own unique stock concentration and material properties. The factor was derived empirically by adjustment of the pH of a prepared collagen gel to 7.4, using the previously described pH measurement paper. *Starting point: p = 0.02.*

Fixation and immunofluorescent staining of spheroids in a 3D collagen matrix:

- Inspect the embedded spheroids under microscope to judge if the spheroids display a 3D invasion phenotype. Spheroids that display significant migration along 2D surfaces should be discarded;
- Remove the medium from the wells with multichannel pipette;
- Add 200 µl PBS;
- Remove the PBS and repeat this step;
- Add 200 µl 4% PFA per well and incubate for 15 minutes at room temperature;
- Remove PFA solution and wash 4x 15 minutes with 200 µl PBS at room temperature;
- Permeabilize and block with 200 µl per well labeling solution (see below) for 1 hour, at room temperature;
- Dilute primary antibodies in labeling solution;
- Remove all liquid from each well, gently!
- Add 150 µl primary antibodies in labeling solution to each well and incubate overnight on a shaker at 4°C;
- Remove almost all liquid from each well, gently!
- Wash 5 x 15 minutes with 200 µl PBS + 0.1 % Tween;
- Dilute secondary antibodies, Phalloidin 633 and DAPI in labeling solution;
- Remove PBS + 0.1% Tween from wells and add 150 µl of secondary antibodies in labeling solution to each well;
- Incubate for 4 hours on a shaker at room temperature, in the dark;
- Wash 5x 15 min with PBS + 0.1% tween 200 µl;
- Remove PBS and add 200 µl PBS with 0.05% azide to each well;
- The plate must be stored (preferably <48 hours) at 4°C prior to imaging.

Antibodies and labeling reagents:

- DAPI (Sigma, D9542): stock concentration at 1 mg/ml in water. Stain with 2 µg/ml. Dilute in steps to prevent precipitation;
- AlexaFluor633-Phalloidin (Molecular Probes, A22284): apply at a 1:200 dilution;
- Primary antibody YAP, rabbit mAb IgG (D8H1X, Cell Signaling Technology, #14074): stock concentration 5.5 µg/ml. Apply at a 1:200 dilution (dilute to 0.0275 µg/ml);
- Isotype control, rabbit mAb IgG (DA1E, Cell Signaling Technology, #3900): stock concentration 2.5 mg/ml. Dilute to 0.0275 µg/ml;

- Secondary antibody, highly cross absorbed goat-ant-rabbit IgG1 AlexaFluor488 (Thermo Fischer Scientific, A11034 2017, 1:1 in glycerol): stock concentration 2 mg/ml. Apply at a 1:200 dilution (dilute to 10 µg/ml);
- 10% BSA solution is freshly prepared from BSA powder (Fraction V, Biomol) in MilliQ.
- Labeling solution is freshly prepared from 0.3 % Triton X-100 in PBS, 10 % normal goat serum and 1 % BSA (from freshly prepared 10 % BSA solution);
- PFA 4 % solution is freshly prepared from frozen (-20°C) PFA 8 % stock solution (PFA dissolved in mQ, which was adjusted with 1N NaOH until the suspension became clear). PFA 8 % is thawed and the suspension is dissolved in a 60°C water bath. An equal volume of 0.2 M Phosphate buffer is added to reach a concentration of 4 % PFA.

Adaptations to the standardized protocols:
To apply the standardized protocols to other cell lines and organoid culture, the following adaptations and additions were made to the hands-on protocols described above:

Spheroid assay with MDA-MB-231 cell line:
- In order to generate 3D spheroids, a double concentration of methyl cellulose (4.8 mg/ml) and addition of 10 µg/ml Purecol Bovine Collagen Solution (Type I, 3 mg/ml, Cell Systems, cat. Nr. 5005-100ML) instead of rattail collagen was used;
- A Spheroids were incubated for 48h to establish a clear invasion phenotype.

Adaptation spheroid assay to CRC organoids (P19TA, #9 neon, #14):
- *Adaptation to protocol for cell culture:* human lung MRC-5 fibroblasts were cultured with the following modifications to the cell culture protocol: cells were maintained in an atmosphere with 5% CO_2. Cells were cultured up to full confluency before use, until a bundled structure of cells was observed.
- *Organoid culture:* the patient-derived CRC organoids were established as part of a living biobank of CRC10, and were kindly provided by the HUB foundation (hub4organoids.eu). Organoids were cultured in 70% Matrigel (Corning, 356231) with basal medium 2+ (BM2+) containing advanced-DMEM/F12 (Life Technologies, 12634028), supplemented with penicillin (100 U/ml) and streptomycin (100 µg/ml; PAA), HEPES (10 mM, Lonza, BE17-737E), GlutaMAX (400 µM, Life Technologies, 35050038), B27 (0.2X, Life Technologies, 17504044), N-Acetyl-L-cysteine (1 mM, Sigma-Aldrich, A9165-5G), Noggin (10%), A83-01 (500 nM, Biovision, 1725-1) and SB202190 (10 µM, Sigma-Aldrich, S7067) at 37°C and 5% CO_2.
- *Adaptations to protocol for generation of 3D spheroids:* organoids were directly harvested from culture, after an incubation period of approximately 12 days. At this timepoint the organoids approached a comparable size to the HT1080 spheroids (approximately 300 µm, for spheroids of 1000 cells).
- *Adaptations to protocol for Spheroid embedding:* in order to harvest the organoids, Matrigel was dissolved with ice cold PBS and organoids are transferred to a 15 ml falcon tube, followed by three washing steps with 10 ml ice cold PBS and centrifugation in between (5 min, 111 g, 4°C). Organoids were resuspended and kept on ice in 300 µl BM2+ medium until embedding in collagen.
  Then, organoids were embedded in a collagen matrix co-cultured with MRC-5 fibroblasts in the following manner: first, MRC5 cells from on-plastic culture (80% confluency) were

detached using Trypsin /EDTA (0.075% /2 mM, Thermo Fisher Scientific) in PBS followed by addition of medium containing 10% FCS. Second, cells were spun down, resuspended in BM2+ and counted. Then, during preparation of the collagen mixture for the first embedding step, the addition of 77 µl of medium was replaced by the addition of 40 µl of BM2+ medium and 37 µl of MRC5 cell suspension (520,000 cells/ml). For the preparation of the collagen mixture of the second embedding step, the BM2+ medium containing the resuspended harvested organoids was used. *Standardization of the organoid height with respect to the bottom of the imaging plate was not possible due to the broad distribution in organoid size. The spheroids from the P19TA, #9 neon and #14 cell lines were embedded in respectively 6, 5, and 5mg/ml collagen.* For long-term culture, BM2+ medium, supplemented with 2.5 % FCS to induce fibroblast elongation and maintain cell vitality, was added. Medium was refreshed at day 3 and every second consecutive day.

Fixation and immunofluorescent staining: At day 11 after embedding, samples were fixed following the procedures above, except for the fixation time of 30 minutes at room temperature. The #9 and #14 organoids were stained with DAPI and Phalloidin633 only.

Ontology terms, candidate for, or part of MIACME:

- Cell culture, dimension: 3D (among other terms downstream of cell culture; e.g. 2D, 2/3D;)
- Cell culture, 3D: Spheroid culture (downstream of 3D, among other terms such as interface assay, sandwich assay, single cell assay, organoid culture, …);
- Cell culture: Co-culture (downstream of cell culture. It must also be possible to note down a second cell line when culturing with different cell lines at the same time);
- Cell culture, 3D, Spheroid culture, method: hanging drop (downstream of spheroid culture);
- Cell culture, 3D, Matrix: rattail collagen I (downstream of 3D, among other terms such as Matrigel, hydrogel);
- Pharmacological inhibitor;
- Labeling type: immunofluorescent (downstream from labeling, among others such as fluorescent reagent, non-fluorescent reagent, immunostaining, immune-gold, ...)
- Labeling type, fluorescent reagent: DAPI (among others like Hoechst, phalloidinAlexa633)
- Labeling type, immunofluorescent: YAP mAb;

References:

[1] D. Del Duca, T. Werbowetski, R.F. Del Maestro, Spheroid preparation from hanging drops: characterization of a model of brain tumor invasion, J. Neuro-Oncol. 67(2004) 295–303.

[2] K. Wolf, M. Te Lindert, M. Krause, S. Alexander, J. Te Riet, A.L. Willis, et al., Physical limits of cell migration: control by ECM space and nuclear deformation and tuning by proteolysis and traction force, J. Cell Biol. 201 (2013) 1069–1084.

[3] A. Haeger, M. Krause, K. Wolf, & P. Friedl, Cell jamming: Collective invasion of mesenchymal tumor cells imposed by tissue confinement, Biochimica et Biophysica Acta - General Subjects, 1840(8), (2014) 2386–2395. https://doi.org/10.1016/j.bbagen.2014.03.020

Appendix II. Standardized microscopy

The following workflow defines the standardized acquisition of microscopy datasets from 3D spheroid cultures. The parameter sets are defined for point scanning confocal microscopy and can be adapted to other microscopy systems, in a platform- and vendor-independent manner.

General settings:

- Sample positioning in the scan field: preferably place the spheroid in the lower left corner, in such a manner that the core border is touching the image border. This can be seen best in the reflection channel.
- Set the Z-range up to 120 µm, starting from invading cells above the spheroid towards roughly the middle of the core (from z= 1/2 to z= 4/5 of spheroid dimensions).
- Detection range: 8 bit.
- Set the following for the optical path and detection configuration:
  - Sequential scanning, to record spectrally overlapping labels as well as reflection and transmission signal independently.
  - Scan 1: 488 nm (YAP) and 633 nm (F-actin; Phalloidin633).
  - Scan 2: 405 nm (DAPI) and 561 nm (reflection and transmission)
- The laser power and amplification (voltage) should be set in such a manner, that the brightest relevant cells make use of the full detection range. It is no problem if cells near the glass or cells in 2D are overexposed. But, deeper cells should be visible with sufficient signal to background, especially in the DAPI channel which is used for segmentation.
- Set laser power as high as possible to get good SNR while scanning fastest, without saturation of the dyes (2x intensity=2x emission).

Microscopy system-dependent settings

1. *Zeiss LSM880*

- Use the following microscope settings:
  - Objective: Zeiss Plan-Apochromat 20x/0.8NA
  - Scan field: 708.5 μm^2
  - Pixels: 608x608, 1.2 µm/pixel
  - Pixel dwell time: 1.3 µs (fastest) with bi-directional scanning (adjust x and y manually)
  - Multi line averaging: 3
  - Optional: increase laser intensity linearly with increasing scan depth in 3D sample to maintain mean fluorescence intensity with the weakest staining as reference. Preferably increase laser power over multiline. At these coarse resolution setting samples do not bleach easily and high laser power is tolerated.
  - Z-depth: 2 µm
- Filter sets for excitation beam path for scan2: 80/20 dichroic mirror, such that reflection can be detected clearly.
- Use the GaAsP detector for the detection of the immunolabeling of weak signals.

2. *Olympus FV1000*

- Use the following microscope settings:
    - Objective: Olympus UPLSAPO 20x air/0.75NA
    - Scan field: 621 μm^2
    - Pixels: 600x600, 0.994 µm/pixel
    - Pixel dwell time: 2 µs
    - Multi line averaging on 1-3, depending on the intensity of the weakest staining. Preferably increase laser power over multiline. At these coarse resolution setting samples do not bleach easily and high laser power is tolerated.
    - Z-depth: 2 µm
- Set the Z-range up to 120 µm, starting from invading cells above towards roughly the middle of the core (from z= 1/2 to z= 4/5 of spheroid dimensions).
- Set the following for the optical path and detection configuration:
    - Sequential 'virtual mode' scanning, to record one immuno-labelling, reflection and transmission signals: Only the laser lines are switching on detector settings are changed, no filter settings are being changed between scans.
- Filter sets detection channels: DM 405/488/559/635, DM490, DM560, Mirror; 430-470, 505-540, 575-675.
- Detectors to be used are: HV 600 (channel 1, DAPI), 600 (Channel 2, 488), 650 (channel 3, phalloidin) or 560 (channel 3, reflection), and 230 (transmission), set the gain to 1.

Ontology terms, candidate for, or part of MIACME:

- Pixel dimension X (downstream of Imaging, Image properties, among pixel dimension Y, Z);
- Type: confocal microscopy (downstream of Imaging, Imaging devices, Microscopy);
- Device name: Zeiss LSM880 (downstream of Imaging, Imaging devices);
- Objective: Zeiss Plan-Apochromat 20x/0.8NA (downstream of Imaging, Imaging devices);
- Channels: DAPI, YAP, phalloidin, reflection, transmission (downstream of Imaging, Imaging devices, Microscopy);

Appendix III. Standardized image analysis

This workflow is implemented in Fiji as the Nucleus Annotation 3D (NA) and the Cell Migration Analyser 3D (CMA) plugin sets and was distributed to 3 independent labs (RUMC, CRICK, UGENT) for standardized analysis of independent datasets from spheroid culture performed in each lab independently. The plugins were distributed among the WP6 partners and the final version plus a more in-depth manual will be deposited at GitHub.

The complete workflow consists of two distinct phases. Both are given as separate workflows below. The first workflow is used to determine the optimal input parameters which will be used during the second workflow in which the image data set is analyzed.

Optimize input parameters workflow

The optimization workflow is used to determine what the best set of input parameters is for the plugin set on the image data set. This workflow should be performed only once per collection of image data, as long as a similar experimental and imaging set up is chosen. Any changes to the setup that affect image parameters such as average cell size, the amount and type of background noise or image resolution, for example, need a re-optimization of the input parameters.

For all steps in which the software is to be used during the workflow it is indicated whether these are to be applied by the operator of the software (O) or automatically performed by the software itself (A).

1. *<u>Select an optimization set</u>*

- From the available images, select a representative set that will be annotated by hand and serve as a 'golden truth' during the optimization.
- Try and include at least one image per condition.
- Partial images are fine to use, as long as the part is rectangular in form and it is well defined which part of the image is to be used. Crop the image to size.
- Note that these images should ideally not be used in the actual data gathering after optimization. Please note that only the nucleus and actin signal is used for this purpose, so any images that may have flaws in other channels are a good choice for this set.

2. *<u>Create a 'golden truth' annotation set</u>*

The following steps are needed to create a golden truth for each image in the optimization set:

- Start the Nucleus Annotation 3D plugin. (O)
- Select an image file to annotate through the dialog. (O)
- If the file has been imported via the BioFormats dialog, confirm this when prompted. (O)
- If the image has multiple channels, select the channel number of the nucleus signal. (O)
- Duplicate the chosen channel. (A)
- Adjust the brightness and similar settings to improve visibility if needed and confirm when ready. (O)

- Annotate each nucleus in the image. Follow the dialog explanation of the steps to perform. (O)
  Steps 1, 2, and 3a are obligatory for parameter tuning on just segmentation. If migration mode analysis is to be used during measurements, either step 3b, 3c, or 3d is needed for every cell as well.
- When finished with all the nuclei in the image click 'Done'. (O)
- Save the annotation next to the image in a file with the same name but the '_corrections.txt' extension. (O)

3. *Test parameter settings against annotation*

For each annotated image in the optimization set, perform the following steps:

- Open the image. (O)
- Start the Marker Image Creator 3D plugin. (O)
- Select 'Manual points' in the dialog. (O)
- Find and select the annotated 'corrections' file for this image in the file chooser dialog. (O)
- Select the directory in which the marker files will be saved. (O)
- Create the sub-directories 'Marker_Files' and 'Marker_images' in the chosen directory if they do not exist yet. (A)
- Save the marker file and marker image. (A)
- Use the Marker Image Creator 3D plugin as described in the Image Analysis Workflow. (O)
- Use the following as initial input parameters:
  - To find the minimal and maximal nucleus size parameters, measure some nucleus sizes by hand using the straight-line ROI tool:
    - Find the slice on which the nucleus is at its largest.
    - Draw a line ROI from one side of the nucleus to the other and note the distance displayed on the FIJI main dialog. Measure across an average part of the nucleus here, so try to avoid any dents or bulges.
    - Measure both nuclei that are smaller and larger to determine a good lower and upper boundary for the nucleus size. Once again, avoid outliers and instead focus on nucleus sizes that are common but on the low or high end of the scale.
    - 3 or 4 nuclei of a smaller size should suffice to find an average minimal nucleus size and a similar number of larger nuclei will be needed for the maximum nucleus size.
  - Subtract the higher size value from the lower value and halve the difference for the step size (round up to one decimal behind the decimal point). This will result in three steps during the LoG filtering.
  - For Noise, use the value of 1.
  - As Minimum Value, use ⅓ of the 'Maximum size of nucleus' parameter.
  - The RadiusXY value should similarly start at ⅓ of the 'Minimum size of nucleus'.
- The segmentation needs a choice of either a 3D mean or 3D median filter followed by a threshold. Determine a combination that looks good by eye as your initial parameters. One combination is needed for the nucleus channel and one for the actin channel. (O)

- Use the Marker Controlled Watershed 3D plugin as described in the Image Analysis Workflow below with the initial parameter combinations from the previous step. (O)
- Use the Feature Extraction 3D plugin as described in the Image Analysis Workflow below. (O)
- Review the results and retry the plugins with different parameters until satisfied. (O)

Image Analysis Workflow

The image analysis workflow describes the order in which the plugins are to be used per image. For each plugin, it gives the name of the plugin (with the plugin set in parentheses) and the individual workflow for that plugin. For all the plugin-specific workflow steps it is indicated whether these are to be applied by the operator of the software (O) or automatically performed by the plugin (A).

1. Spheroid Annotation 3D (NA)

- Load the image. (O)
- Identify the channel to annotate on. Usually the reflection channel. (O)
- Duplicate the chosen channel. (A)
- Adjust the channel brightness if desired. (O)
- Select a slice in which the spheroid outline can clearly be identified. Preferably a slice closer to the centre of the spheroid. (O)
- Click to add an annotation point on the edge of the spheroid. Select three points spread out along the edge. (O)
- Once three points have been selected, draw a circle through the three points. (A)
- Adjust the three points (by removing and adding again) until the circle best fits the spheroid edge. (O)
- Select another slice in which the spheroid edge is clearly identifiable. This slice should be considerably higher or lower than the previous slice and still contain an open spheroid edge (i.e. not the top or bottom slices of the spheroid). (O)
- Add one point to the edge of the spheroid. (O)
- Draw a sphere through all four annotation points. Adjust the z-size of the sphere to take the X/Y versus Z resolution ratio account. (A)
- Go through the image to judge the fit of the sphere to the spheroid edge. Adjust if necessary by removing and adding the annotation points. Note that the first slice should always have three points and the latter slice always just one. The one point is best for adjusting the sphere's size, while the three points mostly determine the sphere's position. (O)
- Use the dialog buttons to save or finish the annotation. (O)
- The spheroid annotation file is automatically saved next to the original image. (A)

2. Marker Image Creator 3D (CMA)

- Load the image. (O)
- Select nucleus (DAPI) channel and parameters for the LoG filter and maximum finder. (O)
- Apply 'Median (3D)' filter plugin on DAPI channel. (A)
- For each LoG filter step configured:
    - Apply LoG filter (by means of the LoG3D plugin [1]) on the filtered DAPI channel. (A)
    - Invert the LoG image to get proper maximums. (A)

    - Multiply by the XYRadius input parameter to normalize values for nucleus size. (A)
- If multiple LoG steps were taken, combine the results by taking the maximum value for every pixel position out of all the LoG filter images. (A)
- Apply an optimized variant of the 'Find Maximum…' plugin on the single or combined LoG filter image. (A)
- Create a new, black 3D stack with the sizes of nucleus channel and for all maximum points found points set the pixel value at its coordinates to a one-step incrementing value (starting at 1, so 1, 2, 3, 4, etc.). (A)
- Save the created marker image and a text file containing the coordinates of the maximum points, the label given, and the maximum value itself. (A)

3. Marker Controlled Watershed 3D (CMA)

- Load the image and the marker image. (O)
- Identify the nucleus and actin channels. (O)
- Select the filter and threshold methods for the nucleus and for the actin channel. (O)
- Select the parameter to correct for decreasing intensity with depth. (O)
- For the nucleus first and actin channel second:
    - Filter the channel according to the option chosen. (A)
    - Find the threshold for every slice of the channel. Do not apply the thresholds. (A)
    - For every slice, find the actual threshold by averaging over a number (set by user parameter) of the neighboring slice thresholds. If slice numbers outside of the image are required, take the closest existing slice threshold instead. (A)
    - Apply the averaged threshold for every slice. (A)
    - For the actin channel only, add the nucleus channel thresholded image. This is to ensure that the entire cell is segmented, including the nucleus. (A)
    - Create a 3D distance map for the thresholded image (uses the '3D Distance Map' plugin [2]). (A)
    - Use the distance map and the marker image as input for the marker controlled watershed (uses the plugin of the same name [3]). (A)
- Save both segment images. (A)

4. Feature Extraction 3D (CMA)

- Load the original image and the two segmented images (nucleus and actin). (O)
- Identify the nucleus and actin channels and also select any other channels to measure or alternative channel measurements. (O)
- Select any post-processing steps. (O)
- Load the marker coordinates file as produced by the Marker Image Creator 3D from the default location. (A)
- By means of the label identity in the marker coordinates file and the nucleus and actin segment images, create a list of nucleus and cell segment pairs. (A)

- Measure a configured list of features on the nucleus and cell segments. (This uses the standard measurements of Fiji as well as the methods provided by the plugins of the 3D ImageJ Suite [2] and MorphoLibJ [3]). (A)
- For every nucleus marker, if its coordinates are to close the any image edge (X, Y and Z) as configured by the user, flag the nucleus/cell segment pair as excluded by post-processing. (A)
- Load the spheroid annotation file and for each nucleus marker calculate the distance to the edge and the centre of the annotated spheroid. (A)
- For every additional channel or alternative measurement method measure the intensity of the following according to the measurement method:
    - Nuclear: Measure the intensity at all the coordinates of the nucleus segment. (A)
    - Nuclear centre: Measure the intensity within a radius of 3 around the nucleus marker. (A)
    - Cell: Measure the intensity at all the coordinates of the cell segment. (A)
    - Cell without the nucleus: Measure the intensity at all the coordinates of the cell segment excluding the coordinates of the matching nucleus segment. (A)
    - Nucleus surrounding:
        - Duplicate the cell segmentation image as a binary mask. (A)
        - Erode the masked segments. (A)
        - Measure the average intensity in a band of 2 pixels wide around the border of the nucleus segment. The coordinates measured should fall within the eroded mask, but not within any (other or same) nucleus segment. (A)
- For every cell segment that has not been excluded by post-processing, determine the migration mode:
    - Determine if any other cell segments are adjacent to this segment at any point; i.e. the minimum distance between any of the pixels of this segment and any pixel of another segment is 1. (A)
    - If no adjacency is found, the cell segment is considered a single cell. (A)
    - If any adjacency is found, determine the number of cell segments grouped via adjacency links to this cell segment. (A)
    - If the number is two, this cell is part of a paired migration group. (A)
    - If the number is greater than two it is considered a multi-cell migration group. (A)
    - The largest multi-cell migration group is determined to be the spheroid itself. (A)
- Create an image identifying the cell migration groups and an image identifying the migration mode per cell segment. Also create a duplicate image of the nucleus channel and draw the nucleus markers and the outlines of the nucleus segments on it (the latter in a separate color for excluded nuclei). (A)
- Create data files containing the measured features per nucleus and per cell and a file co
- containing a summary of the values over the entire image. (A)

References:

[1] D. Sage, F.R. Neumann, F. Hediger, S.M. Gasser, M. Unser, "Automatic Tracking of Individual Fluorescence Particles: Application to the Study of Chromosome Dynamics," IEEE Transactions on Image Processing, vol. 14, no. 9, pp. 1372-1383, September 2005

[2] J. Ollion, J. Cochennec, F. Loll, C. Escudé, T. Boudier. TANGO: A Generic Tool for High-throughput 3D Image Analysis for Studying Nuclear Organization. *Bioinformatics* 2013 Jul 15;29(14):1840-1
[3] Legland, D.; Arganda-Carreras, I. & Andrey, P. "MorphoLibJ: integrated library and plugins for mathematical morphology with ImageJ", Bioinformatics (Oxford Univ Press) 32(22): 3532-3534, 2016
