# Supplementary material for: Multisite assessment of reproducibility in high‐content cell migration imaging data
Source: Mol Syst Biol. 2023 Apr 17;19(6):e11490. doi: 10.15252/msb.202211490 (PMC10258559; doi:10.15252/msb.202211490)
Supplement: Supplementary file 5 [file MSB-19-e11490-s003.pdf]

molecular systems biology

Multi-site assessment of reproducibility in high-content cell migration imaging data

Jianjiang Hu, Xavier Serra-Picamal, Gert-Jan Bakker, Marleen Van Troys, Sabina Winograd-Katz, Nil Ege, Xiaowei Gong, Yuliia Didan, Inna Grosheva, Omer Polansky, Karima Bakkali, Evelien Van Hamme, Merijnvan Erp, Manon Vullings, Felix Weiss, Jarama Clucas, Anna Dowbaj, Erik Sahai, Christophe Ampe, Benjamin Geiger, Peter Friedl, Matteo Bottai, and Staffan Strömblad **DOI: 10.15252/msb.202211490**

Corresponding author(s): Staffan Strömblad (staffan.stromblad@ki.se)

Review Timeline:

Submission Date:	2nd Dec 22
Editorial Decision:	16th Jan 23
Revision Received:	2nd Mar 23
Editorial Decision:	23rd Mar 23
Revision Received:	27th Mar 23
Accepted:	30th Mar 23

Editor: Maria Polychronidou

Transaction Report:

(Note: With the exception of the correction of typographical or spelling errors that could be a source of ambiguity, letters and reports are not edited. Depending on transfer agreements, referee reports obtained elsewhere may or may not be included in this compilation. Referee reports are anonymous unless the Referee chooses to sign their reports.)

16th Jan 2023

Manuscript Number: MSB-2022-11490
Title: Multi-site assessment of reproducibility in high-content live cell imaging data

Dear Staffan,

Thank you again for submitting your work to Molecular Systems Biology. We have now heard back from the three reviewers who agreed to evaluate your study. As you will see below, the reviewers appreciate that the addressed topic is relevant for the cell biology field. However, they raise a series of concerns, which we would ask you to address in a revision.

I think that the reviewers' recommendations are rather clear and I therefore see no need to repeat all of the comments listed below. Of note is the point referring to the need to provide clear recommendations for future analyses. Moreover, further methodological details need to be provided and the study should be better contextualized and presented in a way that makes it more accessible to a broad audience. All issues raised by the referees would need to be satisfactorily addressed. Please let me know in case you would like to discuss in further detail any of the issues raised, I would be happy to schedule a call.

On a more editorial level, we would ask you to address the following points:

- Please provide a .doc version of the manuscript text (including legends for the main figures) and individual production quality figure files for the main Figures (one file per figure).

- Please provide 5 keywords.

- We have replaced Supplementary Information by the Expanded View (EV format). In this case, all additional figures can be included in a PDF called Appendix. Appendix Figures should be labeled and called out as: "Appendix Figure S1, Appendix Figure S2, ... etc.". Each Appendix Figure legend should be provided below the corresponding Figure in the Appendix. Please include a Table of Contents in the beginning of the Appendix. For detailed instructions regarding expanded view please refer to our Author Guidelines: .

- All protocols (currently Supplemental files) should be provided in the Appendix and referenced in the main text.

- The two movies should be provided as Movie EV1 and Movie EV2. Please provide each move in a .zip folder containing a README.txt file with the description of the movie.

- Supplemental material 5 should be provided as Dataset EV1. Please include a description in a separate sheet in the .xls file.

- Please provide a "standfirst text" summarizing the study in one or two sentences (approximately 250 characters), three to four "bullet points" highlighting the main findings and a "synopsis image" (550px width and max 400px height, jpeg format) to highlight the paper on our homepage.

- All Materials and Methods need to be described in the main text. We would encourage you to use 'Structured Methods', our new Materials and Methods format. According to this format, the Material and Methods section should include a Reagents and Tools Table (listing key reagents, experimental models, software and relevant equipment and including their sources and relevant identifiers) followed by a Methods and Protocols section in which we encourage the authors to describe their methods using a step-by-step protocol format with bullet points, to facilitate the adoption of the methodologies across labs. More information on how to adhere to this format as well as downloadable templates (.doc or .xls) for the Reagents and Tools Table can be found in our author guidelines: . An example of a Method paper with Structured Methods can be found here:

- Please include a "Disclosure Statement & Competing Interests" in the main text.

- Please include a Data availability section describing how the data, code etc. have been made available. This section needs to be formatted according to the example below:
The datasets and computer code produced in this study are available in the following databases:
- Chip-Seq data: Gene Expression Omnibus GSE46748 (https://www.ncbi.nlm.nih.gov/geo/query/acc.cgi?acc=GSE46748)
- Modeling computer scripts: GitHub (https://github.com/SysBioChalmers/GECKO/releases/tag/v1.0)
- [data type]: [full name of the resource] [accession number/identifier] ([doi or URL or identifiers.org/DATABASE:ACCESSION])

- The References should be formatted according to the Molecular Systems Biology reference style.

- For data quantification: please specify the name of the statistical test used to generate error bars and P values, the number (n)

of independent experiments (specify technical or biological replicates) underlying each data point and the test used to calculate p-values in each figure legend. The figure legends should contain a basic description of n, P and the test applied. Graphs must include a description of the bars and the error bars (s.d., s.e.m.).

- When you resubmit your manuscript, please download our CHECKLIST (https://bit.ly/EMBOPressAuthorChecklist) and include the completed form in your submission.
Please note that the Author Checklist will be published alongside the paper as part of the transparent process (https://www.embopress.org/page/journal/17444292/authorguide#transparentprocess).

If you feel you can satisfactorily deal with these points and those listed by the referees, you may wish to submit a revised version of your manuscript. Please attach a covering letter giving details of the way in which you have handled each of the points raised by the referees. A revised manuscript will be once again subject to review and you probably understand that we can give you no guarantee at this stage that the eventual outcome will be favorable.

Kind regards,

Maria

Maria Polychronidou, PhD
Senior Editor
Molecular Systems Biology

We realize that it is difficult to revise to a specific deadline. In the interest of protecting the conceptual advance provided by the work, we recommend a revision within 3 months (16th Apr 2023). Please discuss the revision progress ahead of this time with the editor if you require more time to complete the revisions. Use the link below to submit your revision:

https://msb.msubmit.net/cgi-bin/main.plex

IMPORTANT: When you send your revision, we will require the following items:
1. the manuscript text in LaTeX, RTF or MS Word format
2. a letter with a detailed description of the changes made in response to the referees. Please specify clearly the exact places in the text (pages and paragraphs) where each change has been made in response to each specific comment given
3. three to four 'bullet points' highlighting the main findings of your study
4. a short 'blurb' text summarizing in two sentences the study (max. 250 characters)
5. a 'thumbnail image' (550px width and max 400px height, Illustrator, PowerPoint or jpeg format), which can be used as 'visual title' for the synopsis section of your paper.
6. Please include an author contributions statement after the Acknowledgements section (see https://www.embopress.org/page/journal/17444292/authorguide)
7. Please complete the CHECKLIST available at (https://bit.ly/EMBOPressAuthorChecklist).
Please note that the Author Checklist will be published alongside the paper as part of the transparent process (https://www.embopress.org/page/journal/17444292/authorguide#transparentprocess).
8. When assembling figures, please refer to our figure preparation guideline in order to ensure proper formatting and readability in print as well as on screen:
https://bit.ly/EMBOPressFigurePreparationGuideline
See also figure legend guidelines: https://www.embopress.org/page/journal/17444292/authorguide#figureformat
9. Please note that corresponding authors are required to supply an ORCID ID for their name upon submission of a revised manuscript (EMBO Press signed a joint statement to encourage ORCID adoption).
(https://www.embopress.org/page/journal/17444292/authorguide#editorialprocess)
Currently, our records indicate that the ORCID for your account is 0000-0002-1236-6339.

Please click the link below to modify this ORCID:
Link Not Available

10. At EMBO Press we ask authors to provide source data for the main manuscript figures. Our source data coordinator will contact you to discuss which figure panels we would need source data for and will also provide you with helpful tips on how to upload and organize the files.

The system will prompt you to fill in your funding and payment information. This will allow Wiley to send you a quote for the article processing charge (APC) in case of acceptance. This quote takes into account any reduction or fee waivers that you may be eligible for. Authors do not need to pay any fees before their manuscript is accepted and transferred to the publisher.

EMBO Press participates in many Publish and Read agreements that allow authors to publish Open Access with reduced/no

publication charges. Check your eligibility: https://authorservices.wiley.com/author-resources/Journal-Authors/open-access/affiliation-policies-payments/index.html

As a matter of course, please make sure that you have correctly followed the instructions for authors as given on the submission website.

*** PLEASE NOTE *** As part of the EMBO Press transparent editorial process initiative (see our Editorial at https://dx.doi.org/10.1038/msb.2010.72), Molecular Systems Biology publishes online a Review Process File with each accepted manuscripts. This file will be published in conjunction with your paper and will include the anonymous referee reports, your point-by-point response and all pertinent correspondence relating to the manuscript. If you do NOT want this File to be published, please inform the editorial office at msb@embo.org within 14 days upon receipt of the present letter.

Reviewer #1:

Summary

The study by Hu et al. describes a multi-laboratory study addressing the reproducibility crisis in the time-lapse image profiling community. Among 5 laboratories, they distributed crucial regents, protocol, and cell lines and had a simple but state-of-the-art experiment to measure cell migration after ROCK inhibition. Though the cell-line was the same and the experimenters well-trained on the protocols, the authors report significant variance between sites and people performing the experiments that hinder direct meta-analysis.

Although I find this study of general interest and the methods used compelling, I am not sure that using data derived from a perturbation of a specific cell line within a specific protocol provides a sufficiently solid basis to draw broadly generalized conclusions.

This is also my only major remark:

A more elaborate study protocol that includes a second cell line and at least 2 more compound treatments would strongly aid the general applicability of the study. In its current state, the study leaves the reader rather speculating what the next steps would be and if their own study would suffer from the same problems.

Minor points:

1. In the Methods and online repos, there is 3D-spheroid data mentioned that is not included in the results nor discussed in the discussion or figures.

2. I am missing a SOP (standard-operating-procedure) that suggests further necessary steps for standardization, as is accepted in the RNA-Seq community.

Reviewer #2:

Summary
The aim of this paper was to identify and quantify sources of variation in cell migration experiments and attempt to correct for the most confounding effects in order to facilitate meta-analyses of these types of studies. The same live cell migration experiments were performed multiple times by multiple researchers in multiple labs using the same cells and reagents (media, ECM, drugs, etc) but different equipment (microscopes, cameras, climate control). Cell segmentation and tracking were performed using automated analysis tools in one lab. Variability between labs, people, biological and technical replicates, cells, and time-points were quantified using Principal Component Analysis (PCA) and Linear Mixed Effects (LME) models. The greatest source of variation overall was between cells, but technical variation was greatest between labs, followed by between researchers in the same lab. Random and fixed effects were used to compute and remove batch effects to compare the effect of drug perturbation in 2D (ROCK inhibition) and ECM composition in 3D. Removing batch effects dramatically decreased inter-lab variation and improved direct comparison of data between labs.

Key conclusions
Lab-to-lab technical variance is a major limitation to making comparisons of cell migration data and performing meta-analyses of cell studies between different groups. However, removing batch effects can improve reproducibility in perturbation studies.

Methodology/model system

Nine researchers from three different institutions performed live cell migration assays three times each, in triplicate, using the same cells, reagents, and protocol. Time-course data about cell morphology and instantaneous velocity were analysed using CellProfiler and Matlab by one researcher. These data were used to quantify variance and reproducibility in drug perturbation and mathematical models were used to determine batch effects.

General remarks:

This is a well thought out and well executed study that addresses an important topic in cell biology, namely, reproducibility in bioimaging experiments. The lack of reproducibility between groups and researchers is a serious concern, especially for cell migration studies where many 'unknown unknowns' can affect experimental outcomes. Identifying the key sources of variation and, importantly, developing methods to correct for variance or normalise data will help make sense of seemingly contradictory results. This will help streamline and optimise drug development and fundamental research. The methodology is described in detail and the conclusions are soundly supported by the data presented in the figures and extensive supplemental data. The raw data and analysis pipelines are also made available. Although this study specifically addresses live cell migration experiments, the concepts addressed would be of interest to a wider audience, as the same types of issues plague all aspects of cell biology.

Major points:

The manuscript would benefit from a bit more depth about the LME and of the batch effect removal methods. A few sentences that explain the methods without jargon would make the paper more conceptually accessible to a wider audience (e.g. biologists who are not highly conversant in statistics or math modelling).

More information could be provided in the Methods or supplemental material about thresholding across different labs' datasets. This is a critical step and could account for some (or even much) of the technical variation seen in the wild, particularly for cell segmentation.

Does removing batch effects between people in the same lab improve reproducibility? For example, would BER for Lab 2 yield the same or similar trend as seen in L1 and L3? This is an important consideration for many groups, where multiple students or post-docs perform the same experiment and want to combine biological replicates, but find that statistical significance suffers from inter-experimenter variation.

Would the authors suggest that, in future, cell migration experiments include some sort of standard perturbation or set of calibration controls in order to determine batch effects more reliably?

Minor points:

Figure 1: Hard to see the images in panel 1b. White on black (or black on white) and increased contrast would improve visibility. The error bars in panel c and the equivalent charts in the supplemental figures are also hard to make out.

Figure 1 and S1: Is C1 one of the experiments (E1, E2, E3), where E1/2/3 are the means of the technical replicates? It is not clear what the error bars refer to in these panels.

Figure S2: Hard to read the text in panel A.

Figure S3 In the PCA plots, it is not clear what the black dots are and why they are different in this figure but not in the others.

Figure 3 and S4: Font size could be increased to make panels easier to read.

Capitalise Supplemental figure 7 on page 4.

Figure S7: Refers to L1 and L2 but Fig. 3 refers to L4 and L5.

The section on 3D experiments could have its own subheading.

Some references to RNA-seq batch effect removal could be included on page 4. How are these methods similar/different to what was done here?

Reviewer #3:

Hu and Serra-Picamal et al. present a systematic assessment of reproducibility in cell migration experiments. They measured the contribution of each source of potential experimental (biological + technical) variability. This was achieved by replicating live

imaged cell migration experiments, under two experimental conditions, in three independent labs, where three different experimentalists performed multiple technical replicates of each experiment. The authors found that the lab in which the experiment was performed constitutes the main source of variability, and that batch correction (comparison to control experiment in the same lab) reduced this variability. Thus, the authors suggest batch correction as a necessary step toward enabling data integration / meta analyses of perturbation experiments.

This technical topic of reproducibility in cell imaging data is very important, and I am not aware of previous studies aiming at systematic assessments (although I would check in the cell profiling community, see later). The fact that the laboratory that the experiment was performed at is a main source of variability was not surprising. However, the authors' standardization efforts, precisely following the same detailed experimental protocols and pipelines independently in multiple labs by multiple people, and their quantitative analyses, formally established this point for the first time. Moreover, the analyses for sources of variation provided quantitative assessment for all sources of variability. This is the main advance and strength of this technical manuscript that will be of interest to cell biologists and computational cell biologists performing/analyzing high-content image-based phenotyping experiments.

Major comments, concerns and suggestions:
• The study domain of focus is cell migration. Why was this domain selected? In my opinion, "cell migration" should be stated explicitly in the manuscript's title.
• Linear Mixed Effect (page #3). This is a key measurement in this manuscript. Please provide some description and reference in the Results. This is not even described in the Methods (that reference an R package). I was not familiar with this model (and so could other readers), and it was hard to me to follow and assess its suitability without clear justification and the proper reference.
• The topic of reproducibility, or at least batch effect removal, is addressed, at least partially, in high-content image-based phenotypic screens (e.g., methodology in work from Anne Carpenter's lab or her alumni, batch effect removal in practically any high-content screening paper). This literature is not explicitly referenced and discussed here. Please survey the literature and add the relevant context in the Introduction and in the Discussion.
• Batch effect removal (page #4). Batch effect removal is a standard step in high-content image-based phenotypic screens. Usually, the magnitude of the deviation of a perturbation is assessed in respect to controls from the same experiment. The authors highlight RNA-seq experiments, wasn't similar work performed in the context of phenotypic screens? I could not follow the procedure of batch effect removal, also after reading the Methods. Is it a new technique in the field of image-based screens? Isn't it obvious that such a control is critical in screens? This is not well explained in the Results nor in the Discussion. I probably missed here some thing along the way, please clarify (or place in context).
• The quality of some of the figures is very low and it is hard to read information within the figure (mostly image quality/size and x/y-labels). For example, Fig. 1b-c, Fig. 1g, Fig. 3b, e.

Other comments and suggestions:

• Introduction:
o The Cell Image Library - is it still active? Human Cell Atlas - are you sure they host microscopy images? Maybe the reference was supposed to be the Human Protein Atlas? Note that the Human Protein Atlas store imaging data generated internally (it is not a repository for external deposition), and that there are other such resources such as OpenCell, OpenOrganelle, The Allen Institute Cell Explorer, the JUMP Cell Painting consortium.
o Multimot consortium - is it still active? If not, perhaps mentioning it (beyond the funding) is not contributing to the manuscript? In any case, the current way it is presented does not provide any information about this consortium to the reader.
o Five laboratories - this is confusing because most of the results are reported for three labs (and then two additional labs on 3D migration). This could be clarified (3 labs for 2D and 2 labs for 3D) to avoid confusion.

• Results:
o "All microscope-derived image collection, ... was conducted by the Strömblad laboratory" (page #3) - what do the authors mean? The experiments were performed in independent labs.
o It was not clear to me what are the differences between Fig. 2a-b and Supp. Fig. 4a-b. Is it inclusion of both experimental conditions versus just control? In any case, please clarify this in the text and in the figure legend.
o In my opinion, Supp. Fig. 4c-d could be moved to the main figure (Fig. 2). I think that

it conveys the information regarding sources of variability in the clearest sense.
o Missing y-axis label for Fig. 2c-d

• Methods:
o "Statistical analysis was performed using R" - what statistical test were performed and where did the authors report these statistical tests?
o Data availability - I think it would be more sustainable to post the data in a repository such as IDR.

• Discussion:
o Personal opinion. The IDR paper presents a simple example of data integration / meta-analysis. I would recommend referring to this, or another example in the discussion

We thank the editor and the reviewers for their constructive comments that have helped us to significantly improve our manuscript. Also, as requested by the editorial office, we have split up and renamed some of the figures in order to fit to the journal format.
Please find below our responses to each point raised by the reviewers.

Reviewer #1:

Summary

The study by Hu et al. describes a multi-laboratory study addressing the reproducibility crisis in the time-lapse image profiling community. Among 5 laboratories, they distributed crucial regents, protocol, and cell lines and had a simple but state-of-the-art experiment to measure cell migration after ROCK inhibition. Though the cell-line was the same and the experimenters well-trained on the protocols, the authors report significant variance between sites and people performing the experiments that hinder direct meta-analysis.

Although I find this study of general interest and the methods used compelling, I am not sure that using data derived from a perturbation of a specific cell line within a specific protocol provides a sufficiently solid basis to draw broadly generalized conclusions.

This is also my only major remark:

A more elaborate study protocol that includes a second cell line and at least 2 more compound treatments would strongly aid the general applicability of the study. In its current state, the study leaves the reader rather speculating what the next steps would be and if their own study would suffer from the same problems.
In this study, although only one cell line was used, we have performed cell migration experiments in both 2D and 3D environments and with different perturbations. The use of 2D and 3D data broadens the scope and relevance towards different types of applications and user groups.

While we agree that broadening the study to include more examples of biological variability could be of value, it should be noted that the prime focus of this study was on the technical variability (including intra- and inter- lab variability). As such, all key points can be sufficiently addressed by the experimental design in this study using one standardized cell model. It should also be noted that the present study represents a large effort of multiple laboratories that for practical and financial reasons could not be remade with a different design.

It should also be noted that the experimental design and execution in this study is idealized, since the different labs used identical protocol and key reagents. The idea is to keep the experimental procedure as much the same as possible, to highlight the variability caused by less controllable factors and how to deal with this variability. The magnitude of the biological variation (cell population heterogeneity and same cell temporal variability) might be different for distinct cell types, perturbations or degree thereof (e.g., varied compound concentrations), but the main conclusions should remain regarding the technical variance. We have added to the Discussion section that tests of different cell models and perturbations in real-world data (that is less standardized) would constitute a desired follow-

up of this study.

Minor points:

1. In the Methods and online repos, there is 3D-spheroid data mentioned that is not included in the results nor discussed in the discussion or figures.

The 3D-spheroid data is identical with the 3D migration data mentioned in the main text, Figure 5 c, and Appendix Figure S10-11. We apologize if this was not sufficiently clear. We have now clarified in the main text (subtitle of the 3D cell migration experiment added in the Results section), Figure legends, and Methods sections of the paper.

2. I am missing a SOP (standard-operating-procedure) that suggests further necessary steps for standardization, as is accepted in the RNA-Seq community.

We have provided detailed protocols that could be used as SOPs. However, for cell migration, the precise implementation of experiments will necessarily vary depending on the cell model, migration mode, the substrate and purpose of the experiment. We also propose in the Discussion the use of batch effect removal as an SOP for perturbation experiments with quantitative multi-variate microscopy data as a read-out. We have also provided a method and code that is applicable for this purpose.

Reviewer #2:

Summary

The aim of this paper was to identify and quantify sources of variation in cell migration experiments and attempt to correct for the most confounding effects in order to facilitate meta-analyses of these types of studies. The same live cell migration experiments were performed multiple times by multiple researchers in multiple labs using the same cells and reagents (media, ECM, drugs, etc) but different equipment (microscopes, cameras, climate control). Cell segmentation and tracking were performed using automated analysis tools in one lab. Variability between labs, people, biological and technical replicates, cells, and time-points were quantified using Principal Component Analysis (PCA) and Linear Mixed Effects (LME) models. The greatest source of variation overall was between cells, but technical variation was greatest between labs, followed by between researchers in the same lab. Random and fixed effects were used to compute and remove batch effects to compare the effect of drug perturbation in 2D (ROCK inhibition) and ECM composition in 3D. Removing batch effects dramatically decreased inter-lab variation and improved direct comparison of data between labs.

Key conclusions

Lab-to-lab technical variance is a major limitation to making comparisons of cell migration data and performing meta-analyses of cell studies between different groups. However, removing batch effects can improve reproducibility in perturbation studies.

Methodology/model system

Nine researchers from three different institutions performed live cell migration assays three times each, in triplicate, using the same cells, reagents, and protocol. Time-course data about cell morphology and instantaneous velocity were analysed using CellProfiler and Matlab by one researcher. These data were used to quantify variance and reproducibility in

drug perturbation and mathematical models were used to determine batch effects.

General remarks:

This is a well thought out and well executed study that addresses an important topic in cell biology, namely, reproducibility in bioimaging experiments. The lack of reproducibility between groups and researchers is a serious concern, especially for cell migration studies where many 'unknown unknowns' can affect experimental outcomes. Identifying the key sources of variation and, importantly, developing methods to correct for variance or normalise data will help make sense of seemingly contradictory results. This will help streamline and optimise drug development and fundamental research. The methodology is described in detail and the conclusions are soundly supported by the data presented in the figures and extensive supplemental data. The raw data and analysis pipelines are also made available. Although this study specifically addresses live cell migration experiments, the concepts addressed would be of interest to a wider audience, as the same types of issues plague all aspects of cell biology.

Major points:

The manuscript would benefit from a bit more depth about the LME and of the batch effect removal methods. A few sentences that explain the methods without jargon would make the paper more conceptually accessible to a wider audience (e.g. biologists who are not highly conversant in statistics or math modelling).
We thank the reviewer for pointing this out and have expanded the description of the LME model in Results and Methods parts. We have also included additional references related to the LME model.

More information could be provided in the Methods or supplemental material about thresholding across different labs' datasets. This is a critical step and could account for some (or even much) of the technical variation seen in the wild, particularly for cell segmentation.
The segmentation detail was optimized for each microscopy technique (e.g., confocal, widefield) in order to minimize as much as possible any differences in segmentation between the different techniques that would otherwise have created higher variance between laboratories. Gaussian noise with a mean of zero and a standard deviation of 0.00001 was added to the wide field images from Laboratory 2 before the cytoplasm segmentation to reduce the variability between laboratories using different microscopy techniques. All the other CellProfiler pipelines and parameters are the same across all the three laboratories. The resulting cell segmentation shows no significant bias between different laboratories as quantified in Appendix Figure S1. We have expanded the description of this in Methods.

Does removing batch effects between people in the same lab improve reproducibility? For example, would BER for Lab 2 yield the same or similar trend as seen in L1 and L3? This is an important consideration for many groups, where multiple students or post-docs perform the same experiment and want to combine biological replicates, but find that statistical significance suffers from inter-experimenter variation.

We have applied the LME based batch effect removal strategy into the data generated by each individual lab and analyzed the effect on the variance between people in the same laboratory. The results from all the three labs showed significant variance reduction and improved control vs perturbation differences (Appendix Figure S7-9).

Would the authors suggest that, in future, cell migration experiments include some sort of standard perturbation or set of calibration controls in order to determine batch effects more reliably?
Given that the effect of a particular perturbation can be context dependent (e.g., mode of cell migration, ECM coating, cell model), we would not be confident in specifying a generic standard perturbation or calibration control.

Minor points:

Figure 1: Hard to see the images in panel 1b. White on black (or black on white) and increased contrast would improve visibility. The error bars in panel c and the equivalent charts in the supplemental figures are also hard to make out.
We have converted Figure 1b into white on black, increased the contrast, and resized. We have also modified the color of the error bars in Figure 1c and Appendix Figure S1.

Figure 1 and S1: Is C1 one of the experiments (E1, E2, E3), where E1/2/3 are the means of the technical replicates? It is not clear what the error bars refer to in these panels.
We apologize for the unclarity in the figure legend. In Figure 1c and Appendix Figure S1, each line represents one technical replicate. The color of the line indicates which experiment this technical replicate belongs to, and the style of the line shows different technical replicates within the same experiment in the control condition. We have improved the figure legends of Figure 1c and Appendix Figure S1.

Figure S2: Hard to read the text in panel A.
We have resized the font and the whole image. Please see the new Appendix Figure S2.

Figure S3 In the PCA plots, it is not clear what the black dots are and why they are different in this figure but not in the others.
In Figure S3 (now Appendix Figure S3), the black dots in the PCA plots shows the position of the first and second principal components for all the observations from the one lab depicted in each plot. Each observation is the status of one cell at one time point. We have improved the figure legends to clarify this. They are different in this figure because for each subfigure a, b, and c, only the observations from one lab are plotted. The other figures contain observations from all the three labs.

Figure 3 and S4: Font size could be increased to make panels easier to read.
We have increased the font size and the figure size for easier reading.

Capitalise Supplemental figure 7 on page 4.
We have modified this (now Appendix Figure S10).

Figure S7: Refers to L1 and L2 but Fig. 3 refers to L4 and L5.
We apologize for the mis-labeling. We have changed them to L4 and L5 in Appendix Figure S10.

The section on 3D experiments could have its own subheading.
We have added the subheading “Validation of batch effect removal in 3D cell migration” to the 3D experiment part in the Results section. And we have also put the 3D cell migration data in a separate figure (Figure 6).

Some references to RNA-seq batch effect removal could be included on page 4. How are these methods similar/different to what was done here?
We have added references of the RNA-seq and image-based screening (('t Hoen et al., 2013; Chandrasekaran et al., 2021; Giraldez et al., 2018) to the batch effect removal part and the Discussion section. And we have also discussed the similarity as well as the potential future work for the batch effect removal strategy in Discussion.

Reviewer #3:

Hu and Serra-Picamal et al. present a systematic assessment of reproducibility in cell migration experiments. They measured the contribution of each source of potential experimental (biological + technical) variability. This was achieved by replicating live imaged cell migration experiments, under two experimental conditions, in three independent labs, where three different experimentalists performed multiple technical replicates of each experiment. The authors found that the lab in which the experiment was performed constitutes the main source of variability, and that batch correction (comparison to control experiment in the same lab) reduced this variability. Thus, the authors suggest batch correction as a necessary step toward enabling data integration / meta analyses of perturbation experiments.

This technical topic of reproducibility in cell imaging data is very important, and I am not aware of previous studies aiming at systematic assessments (although I would check in the cell profiling community, see later). The fact that the laboratory that the experiment was performed at is a main source of variability was not surprising. However, the authors' standardization efforts, precisely following the same detailed experimental protocols and pipelines independently in multiple labs by multiple people, and their quantitative analyses, formally established this point for the first time. Moreover, the analyses for sources of variation provided quantitative assessment for all sources of variability. This is the main advance and strength of this technical manuscript that will be of interest to cell biologists and computational cell biologists performing/analyzing high-content image-based phenotyping experiments.

Major comments, concerns and suggestions:
• The study domain of focus is cell migration. Why was this domain selected? In my opinion, "cell migration" should be stated explicitly in the manuscript's title.
We selected the cell migration domain because it includes critical dynamic features that change over time, which provides more complex data than fixed image features. We have noted this in the Introduction. We have also added “cell migration” in the title.

• Linear Mixed Effect (page #3). This is a key measurement in this manuscript. Please

provide some description and reference in the Results. This is not even described in the Methods (that reference an R package). I was not familiar with this model (and so could other readers), and it was hard to me to follow and assess its suitability without clear justification and the proper reference.
We have expanded the description of the LME model in Results and Methods parts. We have also included additional references related to the LME model.

• The topic of reproducibility, or at least batch effect removal, is addressed, at least partially, in high-content image-based phenotypic screens (e.g., methodology in work from Anne Carpenter's lab or her alumni, batch effect removal in practically any high-content screening paper). This literature is not explicitly referenced and discussed here. Please survey the literature and add the relevant context in the Introduction and in the Discussion.
We thank the reviewer for the suggestion. We have added and discussed the literature on batch effect removal in image data (Chandrasekaran et al, 2021) in the Introduction, Results, and Discussion sections.

• Batch effect removal (page #4). Batch effect removal is a standard step in high-content image-based phenotypic screens. Usually, the magnitude of the deviation of a perturbation is assessed in respect to controls from the same experiment. The authors highlight RNA-seq experiments, wasn't similar work performed in the context of phenotypic screens? I could not follow the procedure of batch effect removal, also after reading the Methods. Is it a new technique in the field of image-based screens? Isn't it obvious that such a control is critical in screens? This is not well explained in the Results nor in the Discussion. I probably missed here some thing along the way, please clarify (or place in context).
Batch effect removal has started to be applied in image-based screenings (Chandrasekaran et al., 2021; Walton et al, 2022). However, no standard batch effect method has yet been widely established. Instead of assessing the magnitude of the deviation of a perturbation in respect to controls from the same experiment, in this study, we fitted the data with the LME model and then curated each observation by discriminating both the computed random effects and fixed effects derived from the LME model. This batch effect removal performed well in this study, but was not compared to alternative statistical methods. Further studies would be useful that focus on the applicability and results of different batch effect removal methods on different types of real-world imaging-based data. We have discussed this in the Discussion section.

• The quality of some of the figures is very low and it is hard to read information within the figure (mostly image quality/size and x/y-labels). For example, Fig. 1b-c, Fig. 1g, Fig. 3b, e.
We have improved the font size, figure size, labels, and image quality of all these figures.

Other comments and suggestions:

• Introduction:
o The Cell Image Library - is it still active? Human Cell Atlas - are you sure they host microscopy images? Maybe the reference was supposed to be the Human Protein Atlas? Note that the Human Protein Atlas store imaging data generated internally (it is not a repository for external deposition), and that there are other such resources such as OpenCell, OpenOrganelle, The Allen Institute Cell Explorer, the JUMP Cell

Painting consortium.
We thank the reviewer for pointing this out. We have updated the image data archive resources in the Introduction section.

o Multimot consortium - is it still active? If not, perhaps mentioning it (beyond the funding) is not contributing to the manuscript? In any case, the current way it is presented does not provide any information about this consortium to the reader.
As suggested, we have removed the Multimot consortium from the introduction part and just included published work from its members.

o Five laboratories - this is confusing because most of the results are reported for three labs (and then two additional labs on 3D migration). This could be clarified (3 labs for 2D and 2 labs for 3D) to avoid confusion.
As suggested, we have modified this in the Introduction.

• Results:
o "All microscope-derived image collection, ... was conducted by the Strömblad laboratory" (page #3) - what do the authors mean? The experiments were performed in independent labs.
Here the image “collection” meant gathering and assembling the data from multiple labs. We apologize for the confusion and have modified the text in the Results.

o It was not clear to me what are the differences between Fig. 2a-b and Supp. Fig. 4a-b. Is it inclusion of both experimental conditions versus just control? In any case, please clarify this in the text and in the figure legend.
The Supp. Fig. 4a-b (Appendix Figure S4a-b in the revised manuscript) includes the variance from both biological (cell and temporal) and technical (replicate, experiment, person, and lab) sources. We have clarified this in the text and figure legend.

o In my opinion, Supp. Fig. 4c-d could be moved to the main figure (Fig. 2). I think that it conveys the information regarding sources of variability in the clearest sense.
In line with the suggestion, we have added the box plot of the technical variance to the main figure (Fig. 3c in the revised manuscript).

o Missing y-axis label for Fig. 2c-d
We apologize for this overlook. We have added the y-axis label in the revised figure (Figure 3d-e in the revised manuscript).

• Methods:
o "Statistical analysis was performed using R" - what statistical test were performed and where did the authors report these statistical tests?
We only used statistical modeling in this study. Therefore, we have merged this with the data processing and modelling part in Methods with the new subtitle “Data processing and statistical modelling of the 2D cell migration data”. And we have also expanded the linear mixed effect modelling description within this part.

o Data availability - I think it would be more sustainable to post the data in a repository such as IDR.

We have coherently shared all the data, quantified results, and scripts in the SciLifeLab Data Repository, driven by an organization that is long-term government-funded in broad political agreement, and with the outspoken intent to keep these data records permanently. This appears to us to be a sustainable data repository.

• Discussion:

o Personal opinion. The IDR paper presents a simple example of data integration / meta-analysis. I would recommend referring to this, or another example in the discussion

As suggested, we have cited and discussed the IDR paper in the Discussion section.

23rd Mar 2023

Manuscript Number: MSB-2022-11490R
Title: Multi-site assessment of reproducibility in high-content cell migration imaging data

Dear Staffan,

Thank you for sending us your revised manuscript. We have now heard back from the two reviewers who were asked to evaluate your revised study. As you will see below, both reviewers are satisfied with the performed revisions and support publication. Reviewer #3 lists a couple of minor concerns, which we would ask you to address in a revision.

We would ask you to address some remaining editorial issues listed below.

- Our data editors and I have made some minor changes in the text (mainly in the figure legends), please see the attached .doc file. Please make all requested text changes using the attached file and *keeping the "track changes" mode* so that we can easily access the edits made.

- The data and code should be deposited in one of the databases/repositories recommended in our Author Guidelines https://www.embopress.org/page/journal/17444292/authorguide#datadeposition e.g. IDR or Biostudies for imaging data and GitHub for code.

- Please include callouts to Figures 4A, 4B, 5A, 5B and all Appendix figure panels.

- Please reupload the Author Checklist with all fields completed (currently the "Reporting" section has not been completed).

Please resubmit your revised manuscript online, with a covering letter listing amendments and responses to each point raised by the referees. Please resubmit the paper **within one month** and ideally as soon as possible. If we do not receive the revised manuscript within this time period, the file might be closed and any subsequent resubmission would be treated as a new manuscript. Please use the Manuscript Number (above) in all correspondence.

Click on the link below to submit your revised paper.

https://msb.msubmit.net/cgi-bin/main.plex

As a matter of course, please make sure that you have correctly followed the instructions for authors as given on the submission website.

Thank you for submitting this paper to Molecular Systems Biology.

Kind regards,

Maria

Maria Polychronidou, PhD
Senior Editor
Molecular Systems Biology

If you do choose to resubmit, please click on the link below to submit the revision online before 22nd Apr 2023.

https://msb.msubmit.net/cgi-bin/main.plex

IMPORTANT: Please note that corresponding authors are required to supply an ORCID ID for their name upon submission of a revised manuscript (EMBO Press signed a joint statement to encourage ORCID adoption).
(https://www.embopress.org/page/journal/17444292/authorguide#editorialprocess)
Currently, our records indicate that the ORCID for your account is 0000-0002-1236-6339.

Please click the link below to modify this ORCID:
Link Not Available

The system will prompt you to fill in your funding and payment information. This will allow Wiley to send you a quote for the article processing charge (APC) in case of acceptance. This quote takes into account any reduction or fee waivers that you may be eligible for. Authors do not need to pay any fees before their manuscript is accepted and transferred to the publisher.

*** PLEASE NOTE *** As part of the EMBO Press transparent editorial process initiative (see our Editorial at https://dx.doi.org/10.1038/msb.2010.72 , Molecular Systems Biology will publish online a Review Process File to accompany accepted manuscripts. When preparing your letter of response, please be aware that in the event of acceptance, your cover letter/point-by-point document will be included as part of this File, which will be available to the scientific community. More information about this initiative is available in our Instructions to Authors. If you have any questions about this initiative, please contact the editorial office (msb@embo.org).

Reviewer #2:

I am satisfied that the authors have addressed my and the other reviewers' concerns and consider the manuscript acceptable for publication.

Reviewer #3:

The reviewers have addressed my concerns, I have no objection for publication.

Two minor suggestions: (1) Linear Mixed Effects (LME). In my opinion, it is still not clear in the Results. I would recommend including a more intuitive explanation on what is it measuring and how for non-mathematical oriented readers. (2) Page #11 in red "distance, et al.)", I assume "et al" is a typo?

Regarding Reviewer #1 request for more cell lines and experiments. I agree with the authors that their claims regarding technical variability are supported by the data. For obvious reasons, more coordinated experiments between three labs are not practical, and in my opinion, not necessary.

We thank the editor and the reviewers for their constructive comments that have helped us to significantly improve our manuscript.

Please find below our responses to each point raised by the editor and reviewers.

Editor:

- Our data editors and I have made some minor changes in the text (mainly in the figure legends), please see the attached .doc file. Please make all requested text changes using the attached file and *keeping the "track changes" mode* so that we can easily access the edits made.

We modified texts according to the editor and reviewers' suggestions with the "track change" mode on.

- The data and code should be deposited in one of the databases/repositories recommended in our Author Guidelines https://www.embopress.org/page/journal/17444292/authorguide#datadeposition e.g. IDR or Biostudies for imaging data and GitHub for code.

We have deposited our image data and related meta data to BioImage Archive. And we have deposited all the scripts and codes used in this study to GitHub. Links are added in the "Data availability" section.

- Please include callouts to Figures 4A, 4B, 5A, 5B and all Appendix figure panels.

We have mentioned all the above sub figures and all Appendix figure panels in the manuscript. In addition, we added lines between sub figures to make them clearer.

- Please reupload the Author Checklist with all fields completed (currently the "Reporting" section has not been completed).

We apologize for the mistake. We have updated the Author Checklist with all fields completed.

Reviewer #2:

I am satisfied that the authors have addressed my and the other reviewers' concerns and consider the manuscript acceptable for publication.

We thank the reviewer for their work on our manuscript.

Reviewer #3:

The reviewers have addressed my concerns, I have no objection for publication.

We thank the reviewer for their work on our manuscript.

Two minor suggestions: (1) Linear Mixed Effects (LME). In my opinion, it is still not clear in the Results. I would recommend including a more intuitive explanation on what is it measuring and how for non-mathematical oriented readers. (2) Page #11 in red "distance, et al.)", I assume "et al" is a typo?

(1) We have further clarified the description of the LME model in the "Variability sources"

section in Results.
(2) The “et al” indicated the additional 16 parameters. We have modified the text to make this clearer.

Regarding Reviewer #1 request for more cell lines and experiments. I agree with the authors that their claims regarding technical variability are supported by the data. For obvious reasons, more coordinated experiments between three labs are not practical, and in my opinion, not necessary.
We thank the reviewer for their support.

30th Mar 2023

Manuscript number: MSB-2022-11490RR
Title: Multi-site assessment of reproducibility in high-content cell migration imaging data

Dear Staffan,

Thank you again for sending us your revised manuscript. We are now satisfied with the modifications made and I am pleased to inform you that your paper has been accepted for publication.

*** PLEASE NOTE *** As part of the EMBO Publications transparent editorial process initiative (see our Editorial at https://dx.doi.org/10.103/msb.2010.72), Molecular Systems Biology publishes online a Review Process File with each accepted manuscripts. This file will be published in conjunction with your paper and will include the anonymous referee reports, your point- by-point response and all pertinent correspondence relating to the manuscript. If you do NOT want this File to be published, please inform the editorial office at msb@embo.org within 14 days upon receipt of the present letter.

LICENSE AND PAYMENT:
All articles published in Molecular Systems Biology are fully open access: immediately and freely available to read, download and share.

Molecular Systems Biology charges an article processing charge (APC) to cover the publication costs. You, as the corresponding author for this manuscript, should have already received a quote with the article processing fee separately. Please let us know in case this quote has not been received.

Once your article is at Wiley for editorial production, you will receive an email from Wiley's Author Services system, which will ask you to log in and will present you with the publication license form for completion. Within the same system the publication fee can be paid by credit card, an invoice or pro forma can be requested.

Payment of the publication charge and the signed Open Access Agreement form must be received before the article can be published online.

Upon acceptance it is mandatory for you to return the completed payment form. Failure to send back the form may result in a delay in the publication of your paper.

Molecular Systems Biology articles are published under the Creative Commons licence CC BY, which facilitates the sharing of scientific information by reducing legal barriers, while mandating attribution of the source in accordance to standard scholarly practice.

Proofs will be forwarded to you within the next 2-3 weeks.

Thank you very much for submitting your work to Molecular Systems Biology.

Kind regards,

Maria

Maria Polychronidou, PhD
Senior Editor
Molecular Systems Biology

EMBO Press Author Checklist

Corresponding Author Name: Staffan Strömblad	
Journal Submitted to: Molecular Systems Biology	
Manuscript Number: MSB-2022-11490	

USEFUL LINKS FOR COMPLETING THIS FORM
The EMBO Journal - Author Guidelines
EMBO Reports - Author Guidelines
Molecular Systems Biology - Author Guidelines
EMBO Molecular Medicine - Author Guidelines

Reporting Checklist for Life Science Articles (updated January 2022)
This checklist is adapted from Materials Design Analysis Reporting (MDAR) Checklist for Authors. MDAR establishes a minimum set of requirements in transparent reporting in the life sciences (see Statement of Task: 10.31222/osf.io/9sm4x). Please follow the journal's guidelines in preparing your manuscript.
Please note that a copy of this checklist will be published alongside your article.

Abridged guidelines for figures
1. Data
The data shown in figures should satisfy the following conditions:
- the data were obtained and processed according to the field's best practice and are presented to reflect the results of the experiments in an accurate and unbiased manner.
- ideally, figure panels should include only measurements that are directly comparable to each other and obtained with the same assay.
- plots include clearly labeled error bars for independent experiments and sample sizes. Unless justified, error bars should not be shown for technical replicates.
- if n<5, the individual data points from each experiment should be plotted. Any statistical test employed should be justified.
- Source Data should be included to report the data underlying figures according to the guidelines set out in the authorship guidelines on Data Presentation.

2. Captions
Each figure caption should contain the following information, for each panel where they are relevant:
- a specification of the experimental system investigated (eg cell line, species name).
- the assay(s) and method(s) used to carry out the reported observations and measurements.
- an explicit mention of the biological and chemical entity(ies) that are being measured.
- an explicit mention of the biological and chemical entity(ies) that are altered/varied/perturbed in a controlled manner.
- the exact sample size (n) for each experimental group/condition, given as a number, not a range;
- a description of the sample collection allowing the reader to understand whether the samples represent technical or biological replicates (including how many animals, litters, cultures, etc.).
- a statement of how many times the experiment shown was independently replicated in the laboratory.
- definitions of statistical methods and measures:
  - common tests, such as t-test (please specify whether paired vs. unpaired), simple χ2 tests, Wilcoxon and Mann-Whitney tests, can be unambiguously identified by name only, but more complex techniques should be described in the methods section;
  - are tests one-sided or two-sided?
  - are there adjustments for multiple comparisons?
  - exact statistical test results, e.g., P values = x but not P values < x;
  - definition of 'center values' as median or average;
  - definition of error bars as s.d. or s.e.m.

Please complete ALL of the questions below.
Select "Not Applicable" only when the requested information is not relevant for your study.

Materials

Newly Created Materials	Information included in the manuscript?	In which section is the information available? (Reagents and Tools Table, Materials and Methods, Figures, Data Availability Section)
New materials and reagents need to be available; do any restrictions apply?	Not Applicable	

Antibodies	Information included in the manuscript?	In which section is the information available? (Reagents and Tools Table, Materials and Methods, Figures, Data Availability Section)
For **antibodies** provide the following information:<br>- Commercial antibodies: RRID (if possible) or supplier name, catalogue number and or/clone number<br>- Non-commercial: RRID or citation	Yes	Reagents and Tools Table

DNA and RNA sequences	Information included in the manuscript?	In which section is the information available? (Reagents and Tools Table, Materials and Methods, Figures, Data Availability Section)
**Short novel DNA or RNA including primers, probes**: provide the sequences.	Not Applicable	

Cell materials	Information included in the manuscript?	In which section is the information available? (Reagents and Tools Table, Materials and Methods, Figures, Data Availability Section)
**Cell lines:** Provide species information, strain. Provide accession number in repository **OR** supplier name, catalog number, clone number, and/**OR** RRID.	Yes	Reagents and Tools Table
**Primary cultures:** Provide species, strain, sex of origin, genetic modification status.	Not Applicable	
Report if the cell lines were recently **authenticated** (e.g., by STR profiling) and tested for mycoplasma contamination.	Yes	Materials and Methods

Experimental animals	Information included in the manuscript?	In which section is the information available? (Reagents and Tools Table, Materials and Methods, Figures, Data Availability Section)
**Laboratory animals or Model organisms:** Provide species, strain, sex, age, genetic modification status. Provide accession number in repository **OR** supplier name, catalog number, clone number, **OR** RRID.	Not Applicable	
**Animal observed in or captured from the field:** Provide species, sex, and age where possible.	Not Applicable	
Please detail **housing and husbandry conditions**.	Not Applicable	

Plants and microbes	Information included in the manuscript?	In which section is the information available? (Reagents and Tools Table, Materials and Methods, Figures, Data Availability Section)
**Plants:** provide species and strain, ecotype and cultivar where relevant, unique accession number if available, and source (including location for collected wild specimens).	Not Applicable	
**Microbes:** provide species and strain, unique accession number if available, and source.	Not Applicable	

Human research participants	Information included in the manuscript?	In which section is the information available? (Reagents and Tools Table, Materials and Methods, Figures, Data Availability Section)
If collected and within the bounds of privacy constraints report on age, sex and gender or ethnicity for all study participants.	Not Applicable	

Core facilities	Information included in the manuscript?	In which section is the information available? (Reagents and Tools Table, Materials and Methods, Figures, Data Availability Section)
If your work benefited from core facilities, was their service mentioned in the acknowledgments section?	Yes	Acknowledgements

Design

Study protocol	Information included in the manuscript?	In which section is the information available? (Reagents and Tools Table, Materials and Methods, Figures, Data Availability Section)
If study protocol has been **pre-registered, provide DOI in the manuscript**. For clinical trials, provide the trial registration number **OR** cite DOI.	Not Applicable	
Report the **clinical trial registration number** (at ClinicalTrials.gov or equivalent), where applicable.	Not Applicable	

Laboratory protocol	Information included in the manuscript?	In which section is the information available? (Reagents and Tools Table, Materials and Methods, Figures, Data Availability Section)
Provide DOI OR other citation details if **external detailed step-by-step protocols** are available.	Not Applicable	

Experimental study design and statistics	Information included in the manuscript?	In which section is the information available? (Reagents and Tools Table, Materials and Methods, Figures, Data Availability Section)
Include a statement about **sample size** estimate even if no statistical methods were used.	Yes	Materials and Methods
Were any steps taken to minimize the effects of subjective bias when allocating animals/samples to treatment (e.g. **randomization procedure**)? If yes, have they been described?	Not Applicable	
Include a statement about **blinding** even if no blinding was done.	Not Applicable	
Describe **inclusion/exclusion criteria** if samples or animals were excluded from the analysis. Were the criteria pre-established?<br><br>If sample or data points were omitted from analysis, report if this was due to attrition or intentional exclusion and provide justification.	Yes	Materials and Methods
For every figure, are **statistical tests** justified as appropriate? Do the data meet the assumptions of the tests (e.g., normal distribution)? Describe any methods used to assess it. Is there an estimate of variation within each group of data? Is the variance similar between the groups that are being statistically compared?	Yes	Materials and Methods

Sample definition and in-laboratory replication	Information included in the manuscript?	In which section is the information available? (Reagents and Tools Table, Materials and Methods, Figures, Data Availability Section)
In the figure legends: state number of times the experiment was **replicated** in laboratory.	Yes	Figure legends
In the figure legends: define whether data describe **technical or biological replicates**.	Yes	Figure legends

Ethics

Ethics	Information included in the manuscript?	In which section is the information available? (Reagents and Tools Table, Materials and Methods, Figures, Data Availability Section)
Studies involving **human participants**: State details of **authority granting ethics approval** (IRB or equivalent committee(s), provide reference number for approval.	Not Applicable	
Studies involving **human participants**: Include a statement confirming that **informed consent** was obtained from all subjects and that the experiments conformed to the principles set out in the WMA Declaration of Helsinki and the Department of Health and Human Services Belmont Report.	Not Applicable	
Studies involving **human participants:** For publication of **patient photos**, include a statement confirming that consent to publish was obtained.	Not Applicable	
Studies involving experimental **animals**: State details of **authority granting ethics approva**l (IRB or equivalent committee(s), provide reference number for approval. Include a statement of compliance with ethical regulations.	Not Applicable	
Studies involving **specimen and field samples:** State if relevant **permits** obtained, provide details of authority approving study; if none were required, explain why.	Not Applicable	

Dual Use Research of Concern (DURC)	Information included in the manuscript?	In which section is the information available? (Reagents and Tools Table, Materials and Methods, Figures, Data Availability Section)
Could your study fall under dual use research restrictions? Please check biosecurity documents and list of **select agents and toxins** (CDC): https://www.selectagents.gov/sat/list.htm	Not Applicable	
If you used a select agent, is the security level of the lab appropriate and reported in the manuscript?	Not Applicable	
If a study is subject to dual use research of concern regulations, is the name of the **authority granting approval and reference number** for the regulatory approval provided in the manuscript?	Not Applicable	

Reporting

The MDAR framework recommends adoption of discipline-specific guidelines, established and endorsed through community initiatives. Journals have their own policy about requiring specific guidelines and recommendations to complement MDAR.

Adherence to community standards	Information included in the manuscript?	In which section is the information available? (Reagents and Tools Table, Materials and Methods, Figures, Data Availability Section)
State if relevant guidelines or checklists (e.g., **ICMJE, MIBBI, ARRIVE, PRISMA**) have been followed or provided.	Not Applicable	
For **tumor marker prognostic studies,** we recommend that you follow the **REMARK** reporting guidelines (see link list at top right). See author guidelines, under 'Reporting Guidelines'. Please confirm you have followed these guidelines.	Not Applicable	
For **phase II and III randomized controlled trials**, please refer to the **CONSORT** flow diagram (see link list at top right) and submit the CONSORT checklist (see link list at top right) with your submission. See author guidelines, under 'Reporting Guidelines'. Please confirm you have submitted this list.	Not Applicable	

Data Availability

Data availability	Information included in the manuscript?	In which section is the information available? (Reagents and Tools Table, Materials and Methods, Figures, Data Availability Section)
Have **primary datasets** been deposited according to the journal's guidelines (see 'Data Deposition' section) and the respective accession numbers provided in the Data Availability Section?	Yes	Data availability
Were **human clinical and genomic datasets** deposited in a public access-controlled repository in accordance to ethical obligations to the patients and to the applicable consent agreement?	Not Applicable	
Are **computational models** that are central and integral to a study available without restrictions in a machine-readable form? Were the relevant accession numbers or links provided?	Yes	Data availability
If publicly available data were reused, provide the respective **data citations in the reference list**.	Not Applicable	
